# Pharmacological induction of MHC-I expression in tumor cells revitalizes T cell antitumor immunity

**DOI:** 10.1172/jci.insight.177788

**Published:** 2024-08-06

**Authors:** Qian Yu, Yu Dong, Xiaobo Wang, Chenxuan Su, Runkai Zhang, Wei Xu, Shuai Jiang, Yongjun Dang, Wei Jiang

**Affiliations:** 1 Key Laboratory of Metabolism and Molecular Medicine, Ministry of Education, Department of Biochemistry and Molecular Biology, School of Basic Medical Sciences, and; 2 Department of Urology, Zhongshan Hospital, Fudan University, Shanghai, China.; 3 Institute of Immunological Innovation and Translation and; 4 Basic Medicine Research and Innovation Center for Novel Target and Therapeutic Intervention, Ministry of Education, College of Pharmacy, Chongqing Medical University, Chongqing Medical University, Chongqing, China.

**Keywords:** Oncology, Cancer immunotherapy, Drug screens, MHC class 1

## Abstract

Antigen presentation by major histocompatibility complex class I (MHC-I) is crucial for T cell–mediated killing, and aberrant surface MHC-I expression is tightly associated with immune evasion. To address MHC-I downregulation, we conducted a high-throughput flow cytometry screen, identifying bleomycin (BLM) as a potent inducer of cell surface MHC-I expression. BLM-induced MHC-I augmentation rendered tumor cells more susceptible to T cells in coculture assays and enhanced antitumor responses in an adoptive cellular transfer mouse model. Mechanistically, BLM remodeled the tumor immune microenvironment, inducing MHC-I expression in a manner dependent on ataxia-telangiectasia mutated/ataxia telangiectasia and Rad3-related–NF-κB. Furthermore, BLM improved T cell–dependent immunotherapeutic approaches, including bispecific antibody therapy, immune checkpoint therapy, and autologous tumor-infiltrating lymphocyte therapy. Importantly, low-dose BLM treatment in mouse models amplified the antitumor effect of immunotherapy without detectable pulmonary toxicity. In summary, our findings repurpose BLM as a potential inducer of MHC-I, enhancing its expression to improve the efficacy of T cell–based immunotherapy.

## Abstract 2

Bleomycin improves anti-tumor immunity by boosting MHC-I expression

## Graphical abstract

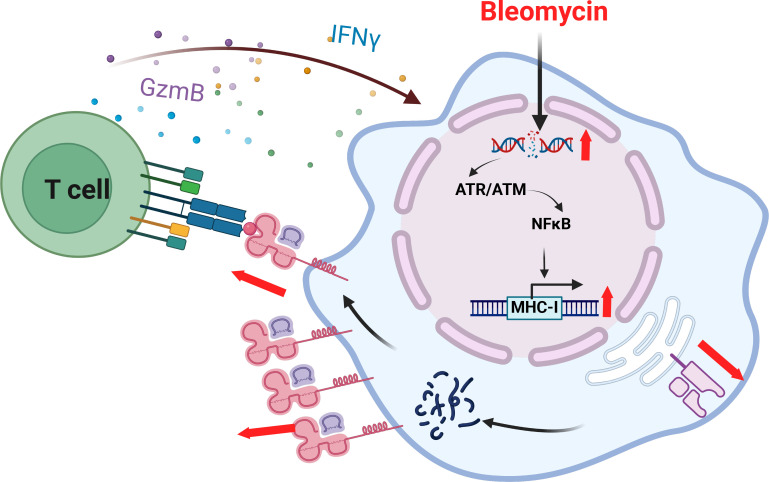

## Introduction

Immunotherapy has revolutionized cancer treatment, demonstrating remarkable clinical efficacy across diverse tumor types (1). Despite these achievements, challenges persist, including variable response rates and the evasion of immune surveillance by malignant cells (2, 3). A pivotal mechanism in immune evasion is the downregulation of cell surface major histocompatibility complex class I (MHC-I), compromising T cell–mediated killing (2, 4, 5).

Human MHC-I molecules, commonly known as HLA, play a crucial role in antigen presentation to T cells and tumor immune escape (6). Previous studies underscore a positive correlation between MHC-I expression and patient prognosis across various cancer types (7). Conversely, downregulation of MHC-I has been associated with disease progression and unfavorable prognosis in diverse cancers, such as breast carcinoma (8), colon carcinoma (9), Hodgkin lymphoma (10), non–small cell lung cancer (11), and bladder carcinomas (12). Importantly, reduced MHC-I expression has been linked to resistance to immune checkpoint therapy (ICT) (13), where the therapeutic efficacy relies on cytotoxic T cells recognizing cytosolic antigens presented by MHC-I on the tumor cell surface (14, 15).

Diverse strategies malignant cells use to subvert immune surveillance underscore the crucial need for identifying effective small molecules capable of overcoming these evasion mechanisms. While immune checkpoint blockage has proven to be a pivotal therapeutic strategy to thwart immunosurveillance, the focus has predominantly centered on antibodies. Small molecules targeting programmed cell death 1/ programmed cell death ligand 1 (PD-1/PD-L1) function have also emerged and entered clinical trials. However, investigations into molecules specifically regulating MHC-I expression have been comparatively limited. Notably, previous observations have demonstrated increased MHC-I expression following treatment with cyclin-dependent kinase 4 and 6 inhibitors or the enhancer of zeste homologue 2 inhibitor GSK126 for 7 days (16, 17), indicating the potential of small molecules in regulating MHC-I expression. However, there is a need to explore effective strategies for improving MHC-I expression and function.

The expression of MHC-I is regulated by multiple regulators and pathways, including interferon regulatory factor 1 (IRF1), STAT1, and NLRC5 (18). Among them, the transcription factor NF-κB plays a crucial role in the regulation of MHC-I (19). Activation of NF-κB was shown to promote increased expression of MHC-I and counter the immune evasion exploited by cancer cells (20, 21).

In this context, the present study aims to address the challenge of low MHC-I expression on tumor cells. Leveraging a high-throughput flow cytometry system, we systematically screened for small molecules capable of rapidly enhancing surface MHC-I expression. Our investigations led to the identification and validation of bleomycin (BLM) as a potent inducer of MHC-I expression in tumor cells. Renowned as an antibiotic chemotherapeutic agent with established applications in various cancers, BLM has been constrained in clinical use by its side effect of lung injury. This investigation delves into the multifaceted effects of BLM. Our study unveils BLM’s ability to promote CD8^+^ T cell activation through antigen-dependent mechanisms, a phenomenon that is substantiated both ex vivo and in vivo. Moreover, BLM emerges as a regulator of the tumor immune microenvironment, inducing MHC-I expression in a manner dependent on ataxia-telangiectasia mutated/ataxia telangiectasia and Rad3-related–NF-κB (ATM/ATR–NF-κB) signaling. Importantly, our findings indicate the synergistic potential of low-dose BLM treatment when combined with immunotherapy and DNA methyltransferase inhibitors (DNMTi), all while avoiding detected toxicity. To validate the translational impact of our study, we extended our findings to a clinically relevant experimental setting. Here, BLM demonstrates its ability to heighten the susceptibility of patient-derived tumor cells to cytotoxicity mediated by autologous tumor-infiltrating lymphocyte (TILs). This not only underscores the potential clinical relevance of BLM but also repurposes it as a key player in enhancing the efficacy of immunotherapeutic interventions.

## Results

### BLM upregulates MHC-I expression.

To identify drugs with the potential to notably upregulate MHC-I expression, we conducted a screening of 2,112 FDA-approved drugs utilizing a high-throughput flow cytometry system based on tumor cell surface MHC-I expression. This screening identified several compounds, including BLM sulfate, etoposide, cabazitaxel, entinostat, and CI994, as robust enhancers of MHC-I expression (Supplemental Figure 1 and Supplemental Table 1; supplemental material available online with this article; https://doi.org/10.1172/jci.insight.177788DS1). Notably, the ability of paclitaxel to promote MHC-I expression in tumor cells has been previously reported (7), explaining the similar capability observed in cabazitaxel, an analog of paclitaxel. Etoposide (7) and histone deacetylase inhibitors such as entinostat and CI994 have also demonstrated their ability to modulate MHC-I expression in cancer cells (22–24). Therefore, we focused our further investigations on BLM, an antibiotic chemotherapeutic drug.

**Figure 1:**
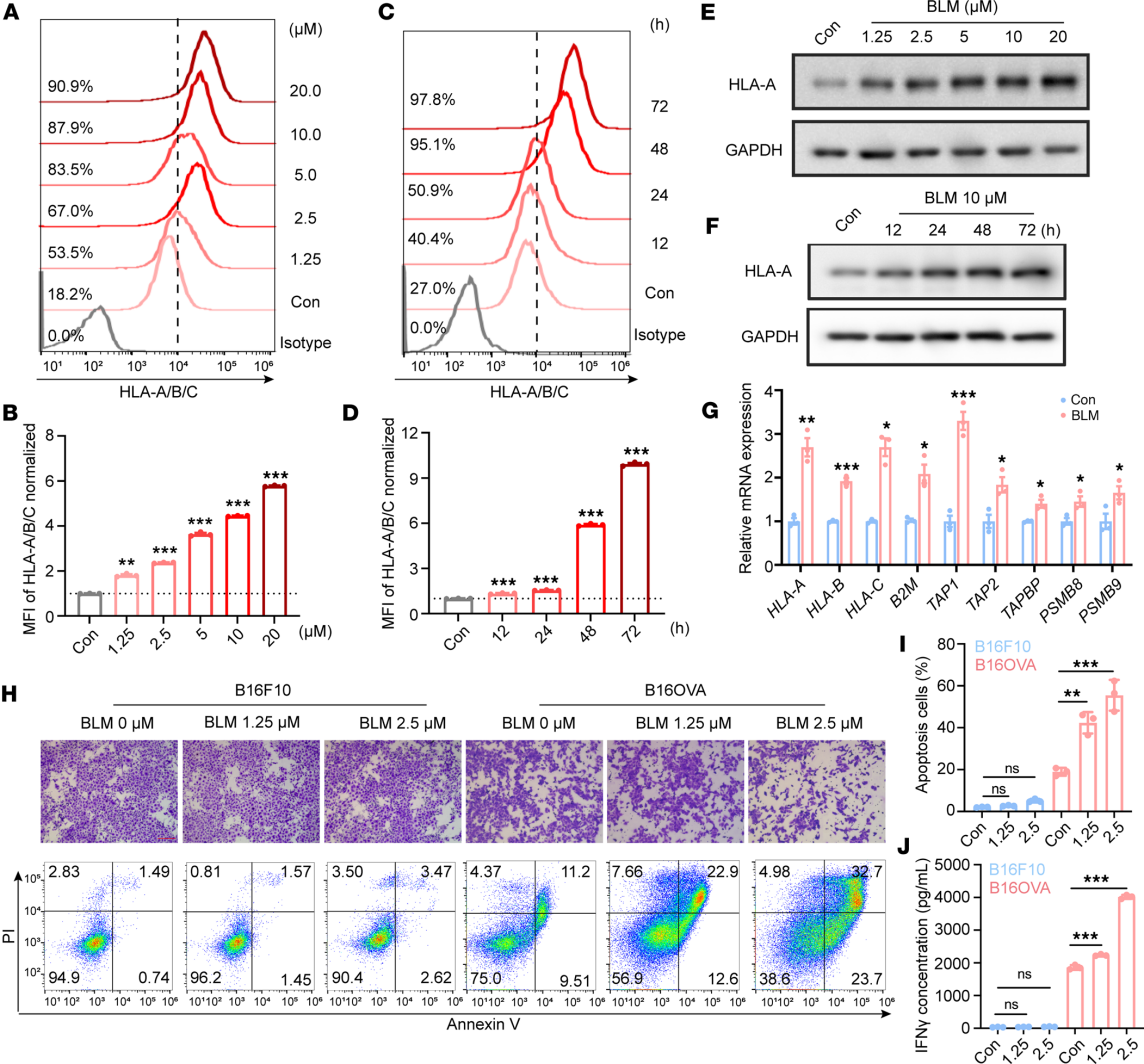
BLM-mediated increasing of MHC-I expression primes CD8^+^ T cell activation. (**A**) Cell surface HLA-A/B/C in SU-DHL-4 cells after incubation with the indicated concentrations of BLM for 48 hours. (**B**) Quantification of mean fluorescence intensities (MFIs) of HLA-A/B/C from **A**; *n* = 3 per group. (**C**) Cell surface HLA-A/B/C in SU-DHL-4 cells following incubation with 10 μM BLM for 12, 24, 48, and 72 hours. (**D**) Quantification of MFI of HLA-A/B/C from **C**; *n* = 3 per group. (**E** and **F**) Western blot analysis of the HLA-A expression in SU-DHL-4 cells after the indicated BLM concentrations (**E**) or BLM treatment times (**F**). (**G**) Quantitative real-time PCR (qRT-PCR) analysis of the antigen presentation gene expression in SU-DHL-4 cells after BLM treatment for 48 hours. (**H**) Coculture of murine cancer cells and OT-I T cells for T cell cytotoxicity assay. B16F10 or B16OVA cells were pretreated with indicated concentrations of BLM for 24 hours prior to coculture with OT-I T cells. The first lane displays the crystal violet staining images of remaining cancer cells (scale bars, 400 μm). The second lane presents the representative images of cancer cells’ apoptosis after coculture with OT-I T cells. PI, propidium iodide. (**I**) Quantification of the percentages of early and late apoptotic cells among cancer cells from **H**; *n* = 3 per group. (**J**) The concentration of IFN-γ in the coculture supernatant as detected by ELISA; *n* = 3 per group. Data indicate the mean ± SD. *P < 0.05, **P < 0.01, ***P < 0.001 compared with the vehicle group by 1-way ANOVA (**B**, **D**, **I**, and **J**) and unpaired *t* test (**G**).

Flow cytometry analysis unequivocally demonstrated a dose- and time-dependent increase in MHC-I cell surface expression in SU-DHL-4 cells following BLM treatment (Figure 1, A–D). In addition, HLA-A protein levels showed a parallel increase in a time- and dose-dependent manner in both SU-DHL-4 (Figure 1, E and F) and SK-BR-3 (Supplemental Figure 2A) cells. Specifically, genes related to MHC-I molecules (*HLA-A*, *HLA-B*, *HLA-C*, and *B2M*), peptide transport (*TAP1* and *TAP2*), transporter-MHC interactions (*TAPBP*), and peptide degradation (*PSMB8* and *PSMB9*) were upregulated in BLM-treated SU-DHL-4 (Figure 1G) and SK-BR-3 (Supplemental Figure 2A) cells. Moreover, in vitro BLM treatment induced increased MHC-I expression in various human tumor cell lines, including T-47D, MDA-MB-231, and BT549 (Supplemental Figure 2, B–D). Also, besides HLA-A, the expression level of HLA-B and HLA-C was also increased following BLM treatment in SK-BR-3 cells, indicating that BLM acted in an allele-independent manner (Supplemental Figure 2E). Importantly, BLM exhibited a lasting effect on the induction of MHC-I expression, persisting even after drug retrieval (Supplemental Figure 2F). Analysis of The Cancer Genome Atlas (TCGA) data verified that the BLM-treated signature correlated with MHC-I expression in human cancers, showing a positive correlation across numerous cancer types with gene signatures of the MHC-I pathway (Supplemental Figure 2F). Notably, in certain cancer types, such as lung squamous cell carcinoma and sarcoma, the BLM-treated signatures exhibited a negative correlation with the pathway (Supplemental Figure 2F), which was possibly due to the heterogeneity between different tumor types.

The BLM-induced increase in cell surface MHC-I expression was also evident in murine tumor cell lines (B16F10, MC38, and MB49) (Supplemental Figure 3, A–C). Western blot analysis further verified a dose- and time-dependent increase in murine B2M protein levels on B16F10 cells following BLM treatment (Supplemental Figure 3D). Similarly, genes encoding murine MHC-I molecules (*H2d1*, *H2k1*, and *B2m*), those governing peptide transport (*Tap1* and *Tap2*), and those involved in peptide degradation (*Psmb9*) were upregulated in BLM-treated murine cells (Supplemental Figure 3E). Collectively, these findings underscore the effective role of BLM in enhancing MHC-I expression in tumor cells.

### BLM-mediated increase in peptide–MHC-I complexes primes CD8^+^ T cell activation by an antigen-dependent mechanism.

Given the observed upregulation of antigen presentation gene expression by BLM, we hypothesized that it might enhance the function of MHC-I in antigen presentation. To investigate this hypothesis, we used a peptide pulsing assay to evaluate cell surface expression of peptide–MHC-I complexes. Following peptide pulsing, B16F10 cells exhibited inadequate presentation of MHC-I–bound SIINFEKL complex, an 8–amino acid peptide derived from OVA. However, pretreatment with BLM dramatically enhanced the expression of these complexes (Supplemental Figure 4, A–D). Similarly, the murine cell line B16OVA, expressing OVA, exhibited an increase in MHC-I–bound SIINFEKL complexes upon BLM treatment, indicating that BLM enhances antigen presentation (Supplemental Figure 4, E and F).

Solid tumors often evade antitumor immunity by downregulating MHC-I surface expression, resulting in reduced recognition and responses by CD8^+^ T cells (7). To investigate whether the increase in peptide–MHC-I complexes induced by BLM enhanced CD8^+^ T cell activation and tumor-killing capability, we conducted a coculture assay of B16OVA tumor cells and OT-I T cells. Importantly, BLM-pretreated B16OVA tumor cells exhibited increased susceptibility to the cytotoxicity of MHC-I–restricted OVA-specific CD8^+^ T cells (preactivated OT-I T cells) compared with the control group. This was evidenced by a lower number of viable tumor cells, a higher apoptosis rate, and increased interferon-γ (IFN-γ) production (Figure 1, H–J). In contrast, B16F10 cells, owing to the absence of the cognate antigen OVA, remained resistant to OT-I T cell killing and failed to induce T cell cytokine production, even when exposed to relatively low concentrations of BLM (Figure 1, H–J). In line with this, TCGA analysis revealed a positive correlation between the BLM-treated signature and CD8^+^ T cell activation in several human cancers (Supplemental Figure 4G). These results clearly demonstrate that BLM treatment sensitizes tumor cells to CD8^+^ T cell–mediated killing.

Additionally, we used human CD8^+^ T cells that were engineered with a recombinant T cell receptor (TCR) targeting the NY-ESO-1 antigen (specifically the NY-ESO-1:157–165 epitope) in an HLA-A*02–restricted fashion (referred to as ESO T cells) (25). Then we performed the coculture assay of NY-ESO-1^+^ SK-BR-3 cells and ESO T cells to examine the effect of BLM in the scenario of human cancer (Supplemental Figure 5, A and B). Consistent with the results from the coculture assay of B16OVA cells and OT-I T cells, NY-ESO-1^+^ SK-BR-3 cells pretreated with BLM showed significantly increased apoptosis rates compared with the control group (Supplemental Figure 5, C and D). This indicates that BLM treatment sensitizes human cancer cells to CD8^+^ T cell–mediated killing.

To examine whether BLM treatment affects antigen-independent activation of T cells, an ex vivo splenocyte culture assay was used (26). Splenocytes treated with BLM or Concanavalin A (Con A, an antigen-independent mitogen) for 24 hours were analyzed using flow cytometry to assess the frequency of CD44^+^ and CD69^+^ markers, which are indicative of T cell activation among CD8^+^ or CD4^+^ T cells (Supplemental Figure 6A). As expected, Con A induced the expression of CD44^+^ and CD69^+^ markers in CD8^+^ cells or CD4^+^ T cells, while BLM treatment had no such effect (Supplemental Figure 6, B–E). This firmly rules out the possibility of antigen-independent activation of T cells following BLM treatment.

In summary, these results verify that BLM treatment promotes CD8^+^ T cell activation through antigen-dependent mechanisms.

### BLM potentiates the antitumor responses of T cells in vivo.

To investigate the antitumor effect of BLM in vivo, we used a combination of BLM and adoptive T cell transfer. We noticed in a previous study that a low dose (5 mg/kg) of BLM moderately reduced tumor volume without significant changes in mouse weight or evident lung toxicity (27). Therefore, we selected 3 mg/kg BLM as an effective antitumor concentration for subsequent experiments.

**Figure 2:**
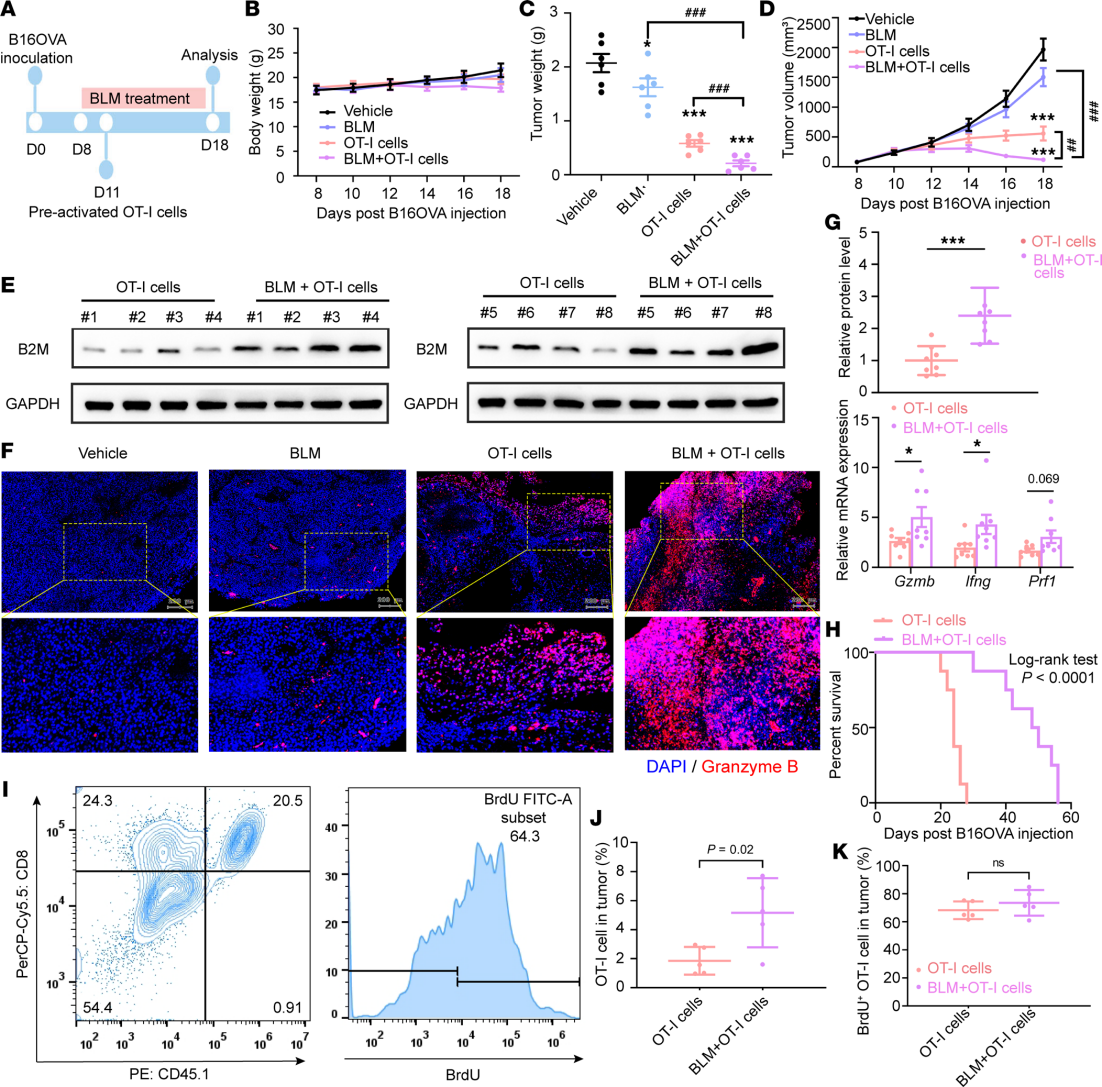
Potentiated antitumor response of T cells by BLM treatment. (**A**) Experimental procedure for the adoptive T cell transfer. (**B**–**D**) Mouse body weight (**B**), tumor weight (**C**), and tumor volume (**D**); *n* = 8 per group. (**E**) Western blot analysis of B2M level in tumor tissues as indicated. (**F**) Mouse melanoma tissues were stained for granzyme B (red) together with DAPI (blue) (scale bars, 200 μm). (**G**) qRT-PCR analysis of gene expression of antitumor effector molecules including granzyme B (*Gzmb*), IFN-γ (*Ifng*), and perforin (*Prf1*) in tumor tissues. (**H**) Kaplan-Meier curves for B16OVA tumor–bearing mice treated with OT-I cells or with the combination treatment of OT-I cells and BLM. (**I**) Representative flow cytometry plot of transferred OT-I T cells (CD45.1^+^CD8^+^) and BrdU staining. (**J** and **K**) Three days after transfer, the frequencies of transferred OT-I cells (CD45.1^+^CD8^+^) (**J**) and BrdU^+^ OT-I cells (**K**) were quantified in tumors from B16OVA tumor–bearing mice pretreated by BLM or not; *n* = 5 per group. Data are shown as mean ± SD. *P < 0.05, and ***P < 0.001 compared with the vehicle group by 1-way ANOVA (**C** and **D**) and unpaired *t* test (**E**, **G**, **J**, and **K**); ^##^*P* < 0.01, and ^###^*P* < 0.001 between the indicated groups by unpaired *t* test (**C** and **D**).

We investigated whether the combination of BLM and the infusion of OT-I cytotoxic T lymphocytes could enhance the killing efficacy of OT-I cells in an adoptive cellular transfer mouse model (Figure 2A). All treated groups exhibited body weights similar to those of the vehicle group (Figure 2B). To assess potential lung toxicity, a primary adverse effect of clinical BLM use in cancer treatment (27), we conducted H&E staining, revealing no discernible lung damage in either the BLM treatment group or the combination treatment group (Supplemental Figure 7).

Mice treated with a relatively low dose of BLM alone did not exhibit a remarkable antitumor response. Infusion of OT-I cells alone led to a slowdown in tumor growth, whereas the combination treatment resulted in further reductions in tumor weight (Figure 2C) and tumor volume (Figure 2D) compared with mono–OT-I cell therapy. Additionally, Western blot analysis demonstrated that BLM elevated B2M expression in tumor samples (Figure 2E). Immunofluorescence staining of tumor tissues revealed slightly increased infiltration of granzyme B^+^ cells in the BLM-treated group compared with that in the vehicle group. Importantly, the combination treatment group exhibited a higher percentage of granzyme B^+^ cells than the mono–OT-I cell therapy group (Figure 2F). Further analysis indicated that combination treatment significantly upregulated the gene expression of granzyme B, IFN-γ, and perforin, secreted by cytotoxic CD8^+^ T cells (Figure 2G). BLM also sensitized B16OVA melanomas to OT-I cell transfer therapy, resulting in a substantial survival benefit (Figure 2H).

To assess whether BLM influenced T cell homing to tumors, we employed BrdU analysis to track T cells’ division in tumors after intravenous transfer of preactivated CD45.1^+^ OT-I cells into B16OVA tumor–bearing mice for 3 days. Notably, the combination treatment group exhibited a higher density of OT-I cells in tumors than the monotherapy group with OT-I cell infusion (Figure 2, I and J). However, there was no difference in the percentage of proliferating OT-I cells among tumors in tumor-bearing mice following treatment with OT-I cells alone or in combination with BLM and OT-I cells, as measured by BrdU incorporation (Figure 2K), indicating that the increased T cell homing, instead of T cell proliferation, was responsible for the effect of BLM.

In summary, these findings suggest that BLM treatment enhances T cell homing to tumors, thereby amplifying the antitumor responses of OT-I cells in adoptive T cell therapy in a mouse model.

### MHC-I is indispensable for the effect of BLM on promoting T cell antitumor immunity.

**Figure 3:**
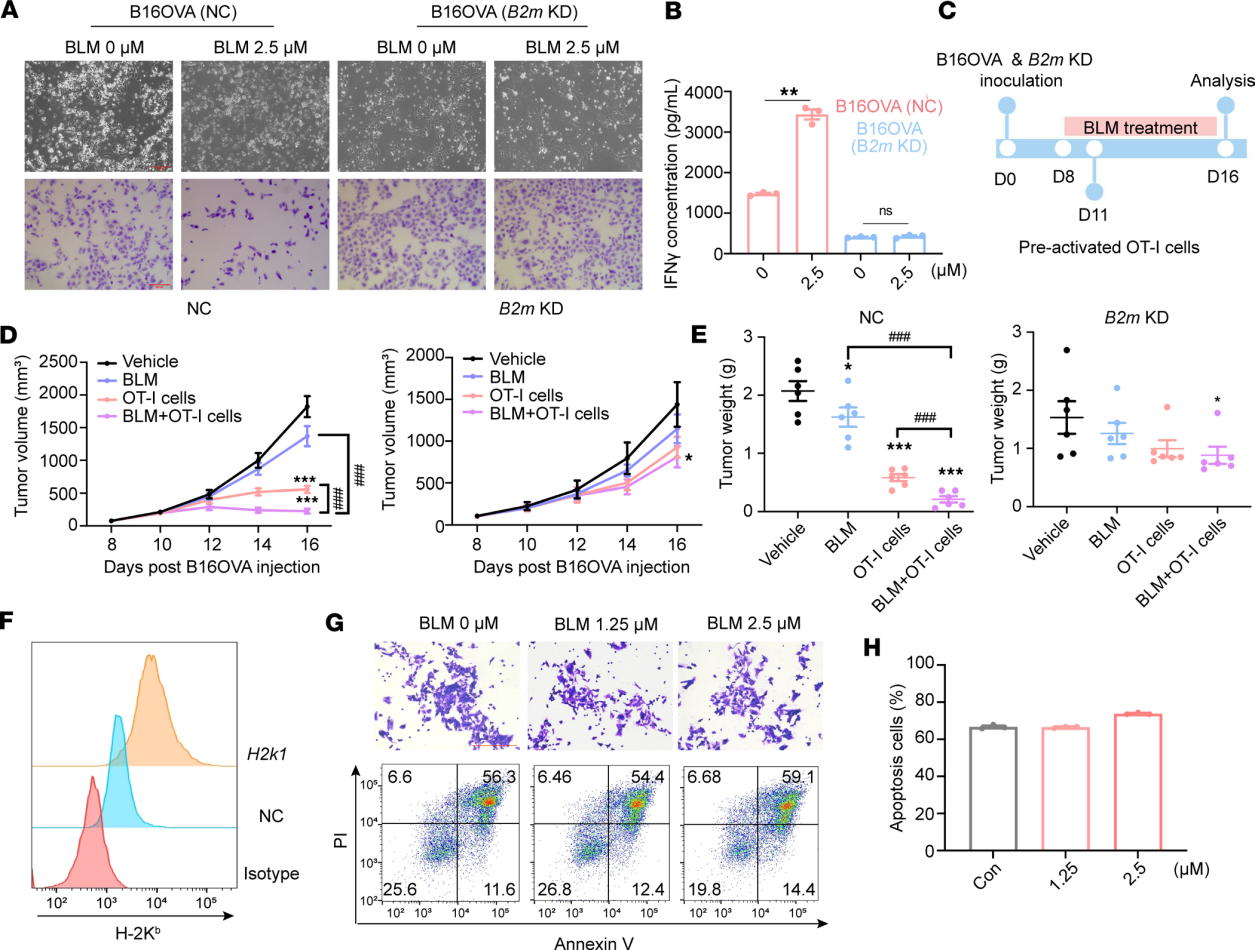
MHC-I in cancer cells is indispensable for the antitumor effect of BLM. (**A**) Coculture of nontargeting control (NC) or *B2m*-knockdown B16OVA cells and OT-I T cells for T cell cytotoxicity assay. Cells were pretreated with indicated concentrations of BLM for 24 hours prior to coculture with OT-I T cells. The first lane shows the microscopy images, and the second lane displays the crystal violet staining images (scale bars, 400 μm). (**B**) Concentration of IFN-γ in the coculture supernatant as detected by ELISA; *n* = 3 per group. (**C**–**E**) The experimental procedure (**C**), tumor volumes (**D**), and tumor weights (**E**) on day 16, *n* = 6 per group. (**F**) Flow cytometry detected cell surface H-2K^b^ expression in NC or *H2k1*-overexpressing B16OVA cells. (**G**) Coculture of B16OVA cells overexpressing *H2k1* and OT-I T cells for T cell cytotoxicity assay. B16OVA cells overexpressing *H2k1* were pretreated with indicated concentrations of BLM for 24 hours prior to coculture with OT-I T cells. The first lane displays the crystal violet staining images of remaining cancer cells (scale bars, 400 μm). The second lane presents the representative images of cancer cells’ apoptosis after coculture with OT-I T cells. (**H**) Quantification of the percentages of early and late apoptotic cells among cancer cells from **G**; *n* = 3 per group. Data are shown as mean ± SD. *P < 0.05, **P < 0.01, ***P < 0.001 compared with the vehicle group by unpaired *t* test (**B**) and 1-way ANOVA (**D** and **E**); ^###^*P* < 0.001 between the indicated groups by unpaired *t* test (**D** and **E**).

To ascertain the role of antigen recognition by cancer cells in BLM’s antitumor effects, we conducted knockdown (KD) experiments targeting *B2m*, a pivotal component of MHC-I molecules, in B16OVA cells (Supplemental Figure 8A). When cocultured with OT-I T cells, BLM enhanced the susceptibility of B16OVA cells to CD8^+^ T cell killing, whereas *B2m*-KD B16OVA cells remained resistant to OT-I T cells (Figure 3A). T cell activation, as indicated by IFN-γ release, returned to the control levels when *B2m* was disrupted in B16OVA cells (Figure 3B). Moreover, we observed that the levels of STAT1 phosphorylation and PD-L1 expression after IFN-γ treatment were unaffected by MHC-I inhibition (Supplemental Figure 8, B–D).

Subsequently, we implanted *B2m*-KD B16OVA clones into mice and assessed the antitumor effect of BLM in combination with OT-I cytotoxic T lymphocyte infusion in adoptive T cell transfer therapy (Figure 3C). While BLM treatment alone led to a modest decrease in tumor growth in the negative control clone, this effect was significantly amplified when combined with OT-I T cells (Figure 3, D and E). In contrast, the disruption of *B2m* in B16OVA cells attenuated the antitumor effect mediated by OT-I T cells, rendering the combination treatment with BLM ineffective in slowing tumor growth (Figure 3, D and E).

To further verify that the increased T cell–killing effect of BLM was dependent on the upregulation of MHC-I expression, we overexpressed the *H2k1* gene in B16OVA cells to increase the surface expression of H-2K^b^ (Figure 3F). In B16OVA cells overexpressing the *H2k1* gene pretreated with BLM, no significant increase in T cell–mediated cytotoxicity was observed when cocultured with OT-I T cells compared to the control group (Figure 3, G and H). This suggests that the effect of BLM is dependent on the upregulation of MHC-I.

In summary, these findings underscore the substantial impact of MHC-I on the antitumor effect of BLM.

### BLM treatment remodels the tumor microenvironment.

**Figure 4:**
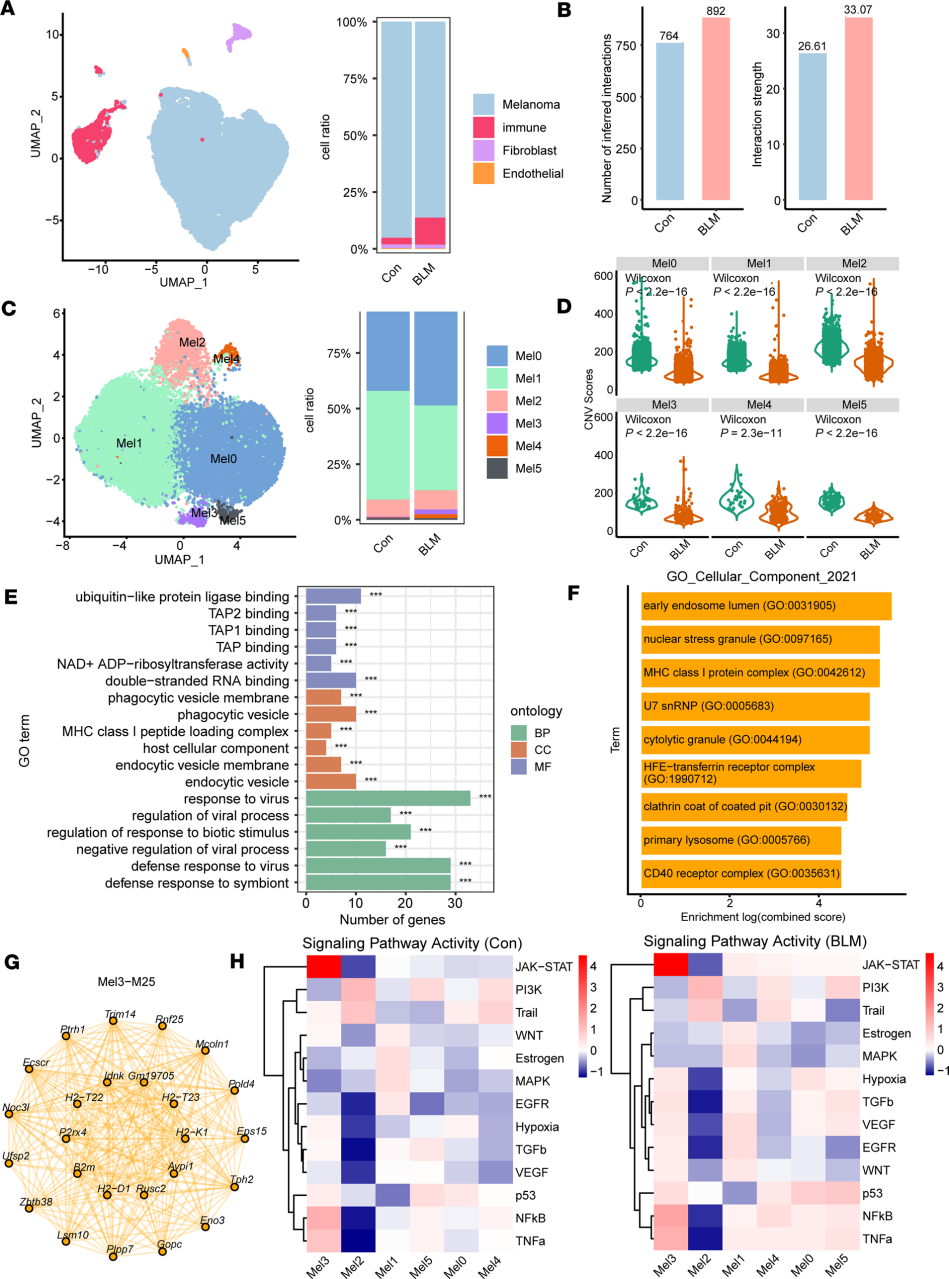
BLM treatment remodels the tumor microenvironment. (**A**) Uniform manifold approximation and projection (UMAP) based on the top 20 principal components of all single-cell transcriptomes color-coded by main cell type and proportion of main cell type per tumor sample. (**B**) The number of inferred interactions and interaction strength were computed by CellChat package among control (Con) and BLM tumor samples. (**C**) UMAP based on the top 20 principal components of all single-cell transcriptomes color-coded by melanoma subclusters and proportion of melanoma subclusters per tumor sample. (**D**) CNV scores among melanoma subclusters (Mel0–Mel5) in different groups were computed by infercnv package. (**E**) GO function enrichment analysis for melanoma subcluster 3 (Mel3) was determined by clusterProfiler package. 44. BP, Biological Process; CC, Cellular Component; MF, Molecular Function. (**F**) The gene set functional analyses of module25 were conducted with enrichR package. (**G**) Network plot visualized the network underlying the top 25 hub genes for module25. (**H**) Mean pathway activity scores of melanoma tumor cells among different subclusters in Con and BLM groups. Data are shown as the exact number of genes associated with each GO term. ***P < 0.001 by Fisher’s exact test.

To investigate the potential molecular mechanism of antitumor responses of BLM treatment in B16-F10 melanoma, we performed unsorted single-cell RNA sequencing, yielding 26,954 high-quality transcriptomes after quality control and filtering (Supplemental Figure 9A). To determine which cellular compartments account for the highest BLM efficacy, we analyzed single-cell transcriptomes for the expression of melanoma, immune, fibroblast, and stromal marker genes. Consistent with the above results, the BLM treatment group exhibited an overrepresentation of immune cell transcriptomes compared with the control group (Figure 4A). Next, we subset and reclustered immune cells into macrophages, monocytes, dendritic cells, T cells, and neutrophils (Supplemental Figure 9B). We used the CellChat package to compute the total number of interactions and interaction strength of the inferred cell-cell communication networks, which were both markedly increased after BLM treatment (Figure 4B). We also observed significantly increased cell-cell interaction strength and interaction numbers among different cell types, especially signals sent from melanoma to other cell types, in the BLM group compared with those in the control groups (Supplemental Figure 9, C and D). To further dissect the influence of BLM within the melanoma cell compartment, melanoma cell transcriptomes were subset and reclustered into 6 subclusters named Mel0 to Mel5 by UMAP analysis (Figure 4C). The copy number variant (CNV) scores in the BLM group among the 6 melanoma subclusters (Mel0 to Mel5) were significantly lower than those in the control group (Figure 4D). Using CytoTRACE, we observed that the Mel3 had the highest CytoTRACE score and was regarded as the starting point of the trajectory by monocle3. We suggested that the ends of the pseudotime trajectories of the other melanoma subclusters were the different end states of the cancer cells (Supplemental Figure 9G). Additionally, we found that the BLM group was more differentiated, which indicated a less malignant phenotype (28) (Supplemental Figure 9H).

Subsequently, we performed Gene Ontology (GO) enrichment analysis to investigate the various biological processes of melanoma subclusters (Supplemental Figure 10, A-C). The Mel1 subcluster enriched DNA proliferative pathways such as DNA replication, nuclear division, and chromosome segregation, suggesting that it was at a relatively high proliferative status (Supplemental Figure 10, A–C). The Mel3 subcluster was enriched for MHC-I–related pathways such as TAP2 binding, TAP1 binding, TAP binding, and MHC class I peptide loading complex (Figure 4E). Therefore, we regarded the Mel3 subcluster as the MHC-I–active subcluster. Additionally, compared with the control group, the BLM group showed an increase in Mel3 subcluster transcriptomes and a decrease in Mel1 subcluster transcriptomes (Figure 4C). Subsequently, we used high-dimensional weighted gene coexpression network analysis to determine the main molecular characteristics of Mel3. We identified 30 gene modules, and the functions of the M25 module were associated with the MHC class I protein complex pathway (Figure 4F). Moreover, the hub genes (*H2-D1*, *H2-K1*, *B2m*, *H2-T22*, and *H2-T23*) of M25 were also closely related to the MHC-I pathway (Figure 4G).

We quantified oncogenic signal strengths using pathway target gene signature expression and discovered that the BLM group exhibited elevated activity in many signaling pathways, including JAK/STAT, NF-κB, TNF-α, p53, VEGF, EGFR, TGF-β, and WNT, as well as in hypoxia-induced pathways. In contrast, the control group exhibited low activity for these pathways (Supplemental Figure 10D). The activities of TGF-β, VEGF, EGFR, WNT, p53, NF-κB, and TNF-α signaling and hypoxia-induced pathways increased to varying degrees after BLM treatment in the Mel3 subcluster, suggesting that these pathways might be involved in the regulation of MHC-I expression (Figure 4H).

Consistent with our previous experimental results, single-cell RNA sequencing analysis further verified that BLM treatment enhanced immune cell infiltration, reduced the degree of malignancy and differentiation potential of melanoma cells, and promoted MHC-I–active subcluster expression, which reveals the antitumor efficacy of BLM.

### MHC-I upregulation caused by BLM depends on ATM/ATR–NF-κB activation.

**Figure 5:**
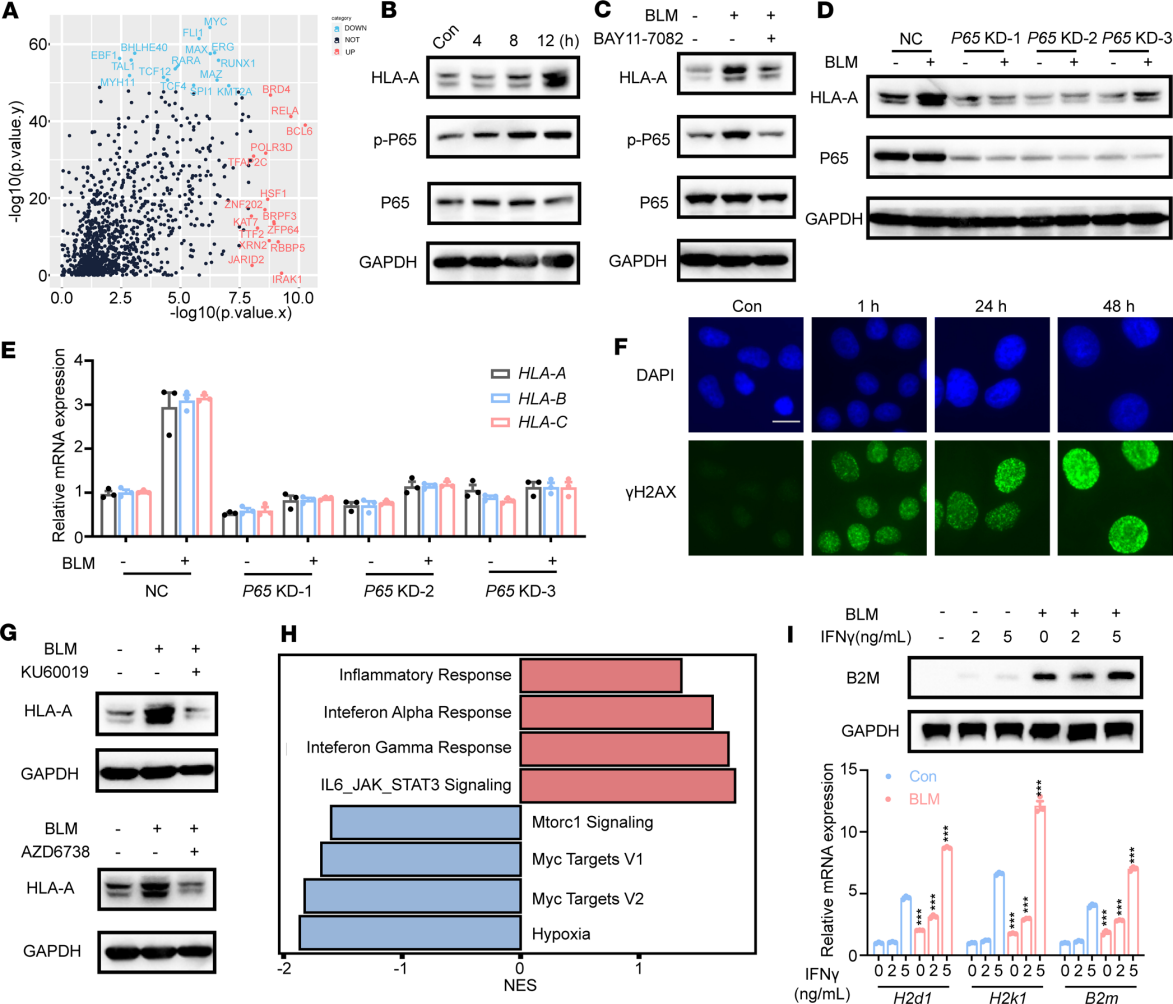
MHC-I upregulation caused by BLM depends on ATM/ATR–NF-κB activation. (**A**) Differentially expressed genes (DEGs) identified in comparisons of BLM-treated cells relative to control were subjected to Lisa (29). The top 30 enriched regulators of upregulated (red) and downregulated (blue) DEGs were noted. (**B**) Western blot analysis of indicated proteins in SK-BR-3 cells treated with 10 μM BLM for the indicated times. (**C**) Western blot analysis of HLA-A, phosphorylatedP65, and P65 expressions. SK-BR-3 cells were pretreated with 5 μM BAY11-7082 for 6 hours, followed by 10 μM BLM for 12 hours. (**D**) HLA-A protein levels examined in *P65*-depleted SK-BR-3 cells 48 hours after BLM treatment. (**E**) qRT-PCR analysis of gene expressions of HLA-A, HLA-B, and HLA-C in *P65*-depleted SK-BR-3 cells 48 hours after BLM treatment. (**F**) Immunofluorescence analysis of dsDNA damage by γH2AX S139 antibody staining (green foci; nuclei labeled with DAPI) in SK-BR-3 cells after the indicated times of BLM treatment (scale bars, 25 μm). (**G**) Western blot analysis of the HLA-A expression in SK-BR-3 cells. Cells were pretreated with 10 μM KU60019 or 10 μM AZD6738, for 6 hours, followed by 10 μM BLM for 12 hours. (**H**) Gene set enrichment analysis of significantly upregulated/downregulated pathways in BLM treatment versus control SK-BR-3 cells. (**I**) Western blot and qRT-PCR analysis of MHC-I expression levels in B16F10 cells after BLM treatment in the presence of 2 or 5 ng/mL IFN-γ for 48 hours. Data are shown as mean ± SD. ***P < 0.001 compared with the vehicle group by 1-way ANOVA.

We used Lisa (29) to identify key transcription factors that drove changes in gene expression caused by BLM treatment, highlighting specific transcription factors linked to BLM. This analysis predicted that RELA proto-oncogene most likely influenced the upregulated differentially expressed genes (Figure 5A). Additionally, MHC-I expression is regulated by various transcription factors that bind to the MHC-I promoter (30). Among these, IRF1 (31), NF-κB (7), and NLRC5 (32) are crucial for the transcriptional upregulation of MHC-I genes following cytokine stimulation. Intriguingly, NF-κB was significantly induced following BLM treatment (Supplemental Figure 11A). Moreover, the levels of phosphorylated p65 markedly increased after BLM treatment (Figure 5B). To investigate whether NF-κB plays a role in BLM-induced MHC-I upregulation, we pretreated SK-BR-3 cells with or without the NF-κB inhibitor BAY11-7082. BAY11-7082 reversed the BLM-induced upregulation of HLA-A and phosphorylated p65 caused by BLM treatment (Figure 5C). Notably, HLA-A expression showed no significant change upon BLM treatment in the *P65*-KD groups (Figure 5D). qRT-PCR analysis verified that the increased mRNA expression of *HLA-A*, *HLA-B*, and *HLA-C* after BLM treatment was also blocked by *P65* knockdown (Figure 5E). Furthermore, BLM treatment led to dose- and time-dependent reduction in IκB protein levels (Supplemental Figure 11, B and C). Collectively, these data indicate that NF-κB activation plays a critical role in BLM-induced MHC-I upregulation.

BLM possesses radiomimetic properties, induces DNA double-strand breaks, and is widely used in clinical chemotherapy for various cancers (33). Consistent with previous studies, we observed dose- and time-dependent increases in the expression of DNA damage response markers, including γH2AX (S139), phosphorylated ATM (S1981), and p53 (S15), following BLM treatment (Supplemental Figure 11, D and E). Furthermore, immunofluorescence revealed an elevated number of γH2AX foci in SK-BR-3 cells after BLM treatment (Figure 5F). To assess whether MHC-I upregulation due to BLM was influenced by the DNA damage response pathway, we pretreated cells with an ATM inhibitor (Ku60019), ATR inhibitor (AZD6738), or DNA-dependent protein kinase, catalytic subunit (DNA-PKcs), inhibitor (NU7441) before subjecting them to BLM treatment for 48 hours. BLM-induced MHC-I upregulation was diminished in ATM or ATR inhibitor–pretreated cells (Figure 5G), not in DNA-PKcs inhibitor–pretreated cells (Supplemental Figure 11F). In summary, these findings suggest that MHC-I upregulation following BLM treatment is contingent on ATM/ATR–NF-κB activation.

The activated cyclic GMP–AMP synthase (cGAS)/stimulator of interferon genes (STING) pathway also promotes MHC-I mRNA expression by increasing the expression of type I interferon (IFN-α/β) (34, 35). Therefore, we explored whether the cGAS/STING pathway is activated by BLM treatment. Our results, following *STING* knockdown, indicated that the cytosolic DNA-sensing pathway was not necessary for MHC-I induction after BLM treatment in SK-BR-3 cells (Supplemental Figure 11G). Similarly, MHC-I expression remained unchanged after BLM treatment in the suppression of *TRAF6* (Supplemental Figure 11H). Additionally, the tumor suppressor p53, a crucial effector of the DNA damage response, is phosphorylated and activated by various DNA damage-inducible kinases, including ATM (36). Previous studies have shown that p53 expression increases following BLM treatment (37). We speculated that p53 played a role in BLM-induced MHC-I upregulation. However, MHC-I expression showed no significant difference after BLM treatment in *TP53*-KD cells (Supplemental Figure 11I). Consequently, p53 was deemed unnecessary for MHC-I induction following BLM treatment.

IFN-γ secreted by activated T cells plays a pivotal role in the activation of cellular immunity and, consequently, the stimulation of antitumor immune responses (38). We investigated whether BLM treatment enhances the IFN-γ–induced transcriptional response in tumor cells. Gene set enrichment analysis showed that IFN-α/γ and inflammatory pathways were transcriptionally activated in BLM-treated tumor cells (Figure 5H). Furthermore, we observed that BLM induced additional MHC-I upregulation in the presence of a relatively low dose of IFN-γ, suggesting that BLM enhances HLA presentation through an IFN-independent mechanism (Figure 5I and Supplemental Figure 11, J and K).

In summary, our data demonstrate that BLM-induced MHC-I upregulation in tumor cells relies on ATM/ATR–NF-κB activation.

### DNA methyltransferase inhibition synergizes with BLM to induce antitumor immune responses.

**Figure 6:**
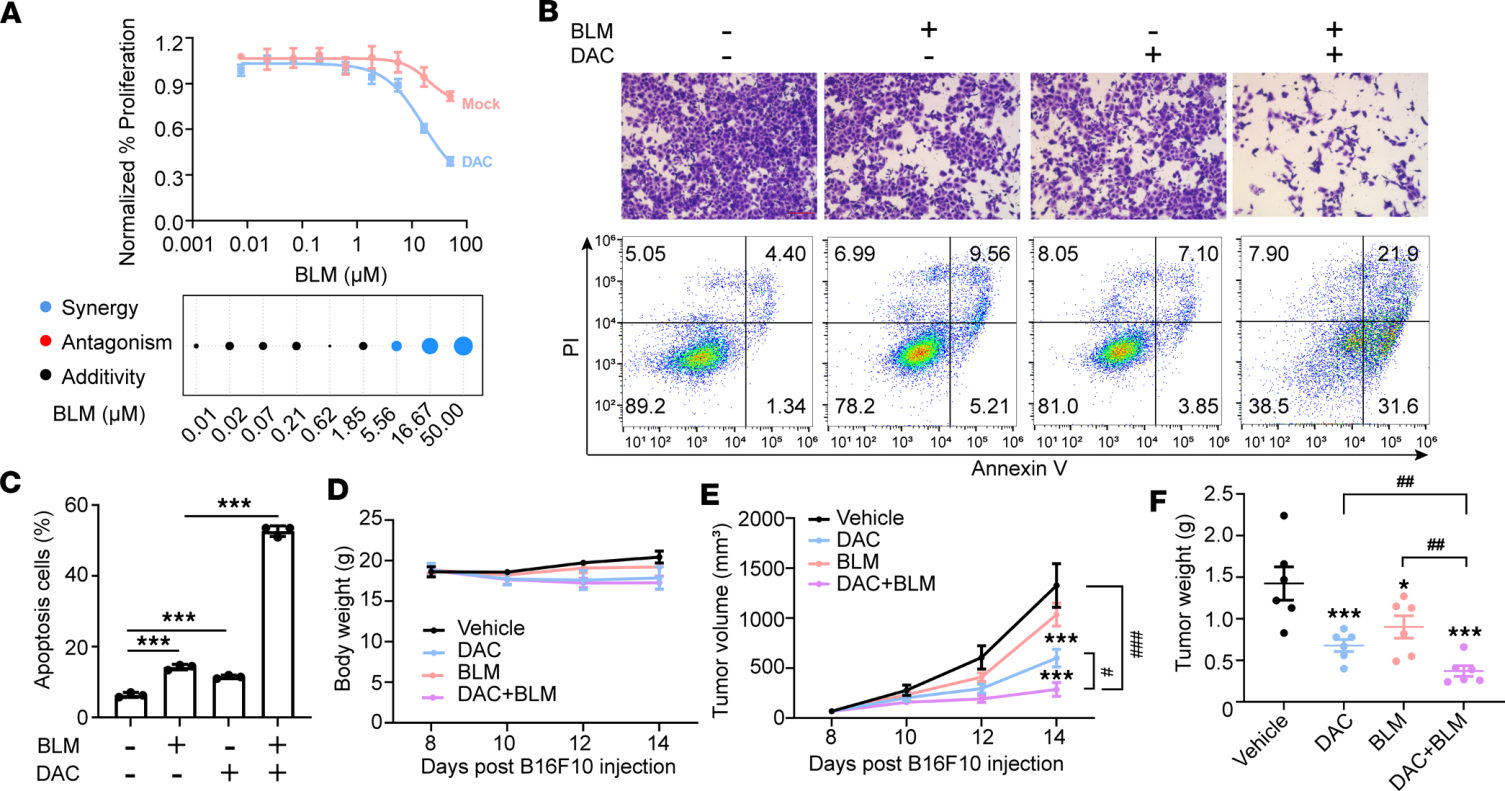
DNA methyltransferase inhibition promotes BLM-induced antitumor immune responses. (**A**) Top panel: Growth inhibition as detected by cell viability of SK-BR-3 cells treated with mock/100 nM decitabine (DAC) for 5 days and BLM for 2 days. Bottom panel: Combination index (CI) plots. (**B**) B16OVA cells pretreated with 100 nM DAC for 5 days were trypsinized and plated in 12-well plates with equal numbers of viable cells. Cells were then treated with BLM (0.5 μM) for 1 day prior to coculture with OT-I T cells. The first lane displays the crystal violet staining images (scale bars, 400 μm). The second lane presents the representative images of cancer cells’ apoptosis after coculture with OT-I T cells. (**C**) Quantification of the percentages of early and late apoptotic cells among cancer cells from **B**; *n* = 3 per group. (**D**–**F**) Treatment of B16F10 tumors with DNA methyltransferase inhibitor (DAC) or vehicle control in combination with BLM or vehicle control; *n* = 6 per group. Mouse weight (**D**), tumor volume (**E**) and tumor weight (**F**); *n* = 6 per group. Data are shown as mean ± SD. *P < 0.05, ***P < 0.001, compared with the vehicle group by 1-way ANOVA (**E** and **F**) and unpaired *t* test (**C**); ^#^*P* < 0.05, ^##^*P* < 0.01, and ^###^*P* < 0.001 between the indicated groups by unpaired *t* test (**E** and **F**).

Cytidine methylation reduces the efficiency and alters the pattern of BLM-mediated cleavage of double-stranded DNA (39). Thus, we posited that DNMTi would promote the antitumor immune effect of BLM. Sequential treatment with DNMTi and BLM demonstrated a synergistic and robust potentiation of BLM by DNMTi in SK-BR-3 cells (Figure 6A and Supplemental Figure 12A). Among the DNMTi tested, DAC in combination with BLM exhibited the most potent antiproliferative effect, compared with azacitidine, and was therefore chosen for further investigation. MHC-I expression was increased with the combined treatment of BLM and DNMTi (Supplemental Figure 12B). Consistent with previous data, both BLM and DAC (39, 40) induced a DNA damage response, as evidenced by γH2AX foci accumulation in SK-BR-3 cells (Supplemental Figure 12, C and D). Furthermore, DNMTi potentiated BLM-mediated DNA damage, as indicated by higher expression of γH2AX S139 and increased γH2AX foci accumulation (Supplemental Figure 12, C and D).

We also observed a potent augmentation of the BLM antiproliferative effect in B16F10 tumor cells when used in combination with DAC (Supplemental Figure 12E). Additionally, we explored the potential of DAC treatment to enhance BLM-induced T cell activation. Neither BLM nor DAC treatment alone resulted in fewer viable cancer cells than the control group after coculture with OT-I T cells. However, there was a significant potentiation of the BLM-induced T cell activation when used in combination with DAC (Figure 6, B and C). We further examined the impact of DAC on tumor response to BLM treatment in vivo by treating established B16F10 melanoma tumors with DAC and/or BLM. There were no significant differences in mouse body weight among the different treatment groups (Figure 6D). Consistent with the in vitro coculture results, DAC treatment alone moderately slowed tumor growth in vivo, and the combination treatment of DAC and BLM further reduced tumor progression (Figure 6, E and F).

In summary, DNMTi enhance the upregulation of MHC-I in tumor cells induced by BLM and facilitate cancer cell killing by T cells, thereby complementing the therapeutic effects of BLM in B16F10 xenografts.

### BLM-mediated potentiation of antitumor responses for T cell–based immunotherapy.

We further investigated the potential of combination therapies with BLM in immunotherapy approaches, such as bispecific antibody therapy, immune checkpoint therapy (ICT), and TIL therapy in solid tumors, whose efficacy relied on T cells and was restricted by MHC-I expression levels.

**Figure 7:**
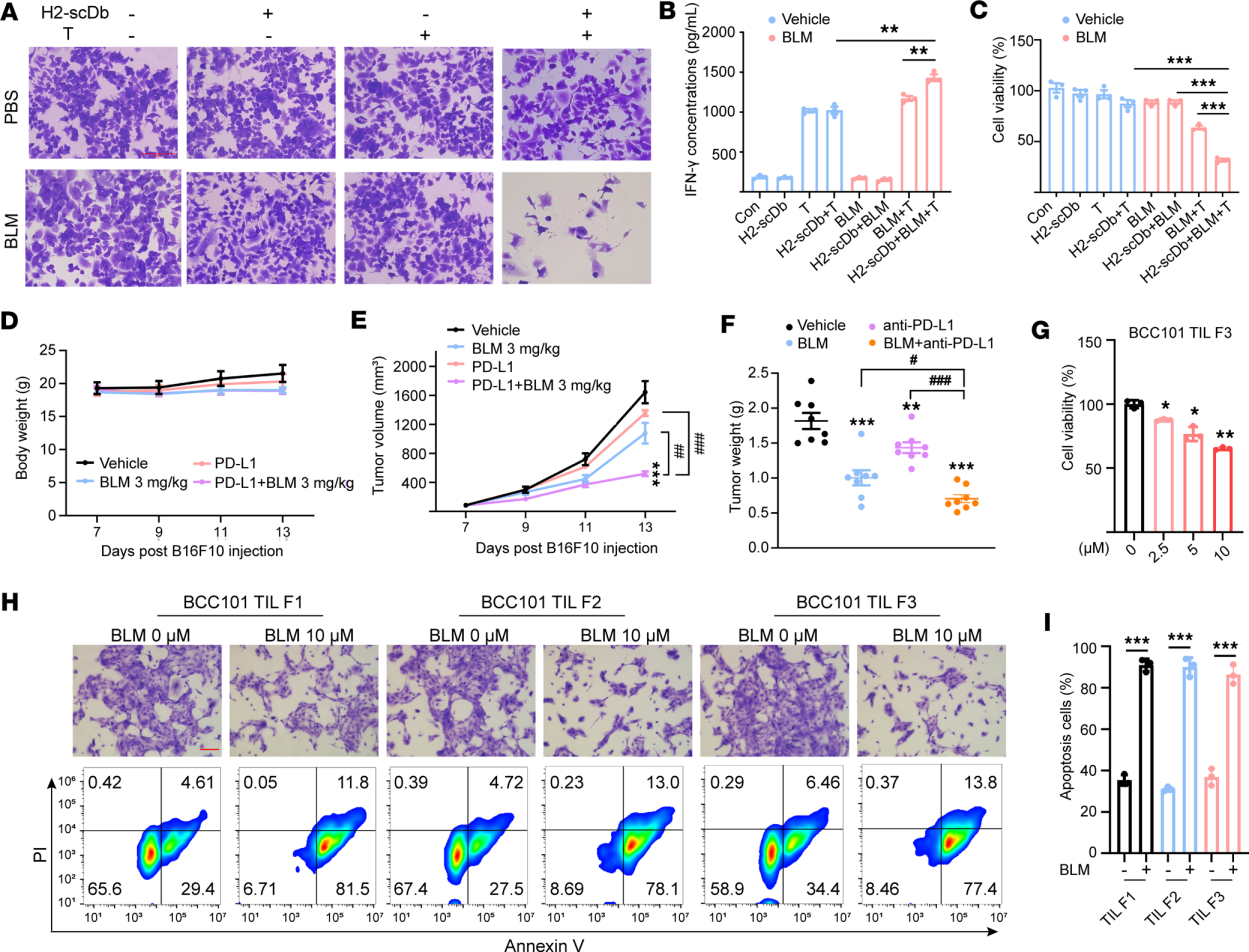
BLM-mediated potentiation of antitumor responses for immunotherapy. (**A**) Coculture of SK-BR-3 cells and human CD8^+^ T cells for T cell cytotoxicity assay. The crystal violet staining images of remaining cancer cells are displayed (scale bars, 400 μm). (**B**) The concentration of IFN-γ in the coculture supernatant as detected by ELISA; *n* = 3 per group. (**C**) T cell activation mediated by BLM and H2-scDb in response to SK-BR-3 cells at effector/target ratio of 2:1 as measured by the CellTiter-Glo reagent. (**D**–**F**) Treatment of B16F10 tumors with BLM or vehicle control in combination with PD-L1 or isotype control antibodies; *n* = 8 per group. (**D**) Mouse weight, (**E**) tumor volumes, and (**F**) tumor weights. (**G**) Coculture of primary bladder cancer cells and TILs for T cell cytotoxicity assay. Primary bladder cancer cells were pretreated with indicated concentrations of BLM for 24 hours. Percentage remaining live cancer cells following 24 hours’ incubation with autologous TILs at a 1:5 ratio. (**H**) Coculture of patient-derived BCC101 tumor cells and TILs (F1, F2, and F3 fragments) for T cell cytotoxicity assay. BCC101 tumor cells were pretreated with BLM (10 μM) for 24 hours prior to coculture with TILs. The first lane displays the crystal violet staining images (scale bars, 400 μm). The second lane presents the representative images of cancer cells’ apoptosis after coculture with TILs. (**I**) Quantification of the percentages of early and late apoptotic cells among cancer cells from **H**; *n* = 3 per group. Data are shown as mean ± SD. *P < 0.05, **P < 0.01, and ***P < 0.001, compared with the vehicle group by unpaired *t* test (**B**, **C**, and **I**) and 1-way ANOVA (**E**–**G**); ^#^*P* < 0.05, ^##^*P* < 0.01, and ^###^*P* < 0.001 between the indicated groups by unpaired *t* test (**E** and **F**).

A recent study introduced a bispecific antibody (H2-scDb) designed to specifically target the most common p53 mutation (R175H) along with a common HLA-A allele (HLA-A*02:01) on the cell surface. The efficacy of H2-scDb is highly correlated with the levels of HLA-A allele (HLA-A*02:01) expressed in tumor cells (41). We demonstrated a dose- and time-dependent increase in surface HLA-A2 levels in SU-DHL-4 (p53^WT^ and HLA-A*02:01) and SK-BR-3 (p53^R175H^ mutation and HLA-A*02:01) cells after BLM treatment (Supplemental Figure 13, A and B). We then examined whether BLM treatment could enhance the ability of H2-scDb to activate T cells. Consistent with previous studies, H2-scDb had no impact on SK-BR-3 cells, which harbored the p53^R175H^ mutation and exhibited relatively low expression of HLA-A*02:01. However, pretreatment with BLM enhanced T cell activation mediated by H2-scDb, resulting in a higher number of tumor cells killed and higher production of IFN-γ (Figure 7, A–C).

Many solid tumors resistant to checkpoint blockade are characterized by a lack of cytotoxic T cell recognition and infiltration (42). Therefore, we hypothesized that BLM might enhance the efficacy of checkpoint blockade by increasing MHC-I expression and promoting T cell infiltration. To investigate whether the antitumor efficacy of PD-L1 blockade could be improved by combination with BLM treatment, we treated B16F10 melanoma–bearing mice with BLM, anti-mouse PD-L1 antibody, or a combination of both. The body weight remained stable across the different treatment groups (Figure 7D). Furthermore, mice treated with BLM or PD-L1 antibody alone showed partial tumor growth inhibition, while BLM sensitized B16F10 melanomas to checkpoint blockade with a PD-L1 antibody, resulting in substantially reduced tumor growth and tumor weight (Figure 7, E and F). Additionally, the effect of BLM was examined in an MC38 mouse model and similar results were obtained (Supplemental Figure 14, A–D).

We next expanded our study to a clinically relevant experimental setting. Tumors with high levels of somatic mutations, such as melanoma and bladder cancer, respond well to immunotherapy with checkpoint blockade therapy or the adoptive transfer of antitumor lymphocytes (43, 44). Therefore, we investigated whether BLM treatment improves the activation of autologous TILs in primary patient-derived bladder cancer cells. First, we successfully established bladder cancer cells from patient tumor tissues and urine samples using a conditional reprogramming technique (45–47). In this study, 8 patients diagnosed with bladder cancer were enrolled (Supplemental Table 7). Among these, 4 primary patient-derived cancer cell lines were established from the patient urine samples (BCC3, BCC16, BCC38, and BCC49), whereas the others were established from the tumor samples (BCC1, BCC15, BCC101, and BCC102). MHC-I expression could be induced by BLM in most of the primary patient-derived cancer cell lines with very few toxic effects (Supplemental Figure 15, A and B).

Among the 4 tumor samples, we selected BCC101 (high response) and BCC102 (low response) based on their level of response to BLM-induced MHC-I expression. Subsequently, TILs from different fragments of the 2 tumor samples (labeled F1, F2, F3…) were expanded ex vivo, and the phenotypes of the expanded TILs were assessed by flow cytometry (Supplemental Figure 16, A–C). As expected, pretreatment with BLM rendered BCC101 cells more susceptible to autologous TIL-mediated cytotoxicity than untreated cells, whereas BCC102 showed less effect (Figure 7G and Supplemental Figure 16D). We verified that BLM had no toxic effects to primary cancer cells within the range of experimental concentrations (Supplemental Figure 16E). Additionally, BLM-pretreated BCC101 cancer cells exhibited increased susceptibility to the cytotoxicity of autologous reactive TIL fractions, which was evidenced by a lower number of viable tumor cells and a higher apoptosis rate (Figure 7, H and I). Collectively, these results suggest that the potential of combination therapy with BLM relies on T cells as key effector cells, such as bispecific antibody therapy, ICT, and TIL therapy for solid-tumor indications.

## Discussion

Reduced surface expression of MHC-I stands as a formidable barrier to the success of immunotherapy. In this study, we present BLM as a promising agent, repurposed for its ability to rapidly induce surface MHC-I expression in tumor cells. This highlights MHC-I as an ideal pharmacological target for enhancing tumor immunity.

Utilizing a high-throughput flow cytometry system, we not only identified BLM but also demonstrated the versatility of our screening strategy to uncover potential immunotherapy-enhancing drugs. Moreover, the induction of downregulated MHC-I expression through BLM treatment presents a therapeutically valuable avenue to improve T cell antitumor immunity, overcoming obstacles posed by MHC-I expression limitations and opening possibilities for combination therapies.

BLM, traditionally an antibiotic chemotherapeutic agent with established use in various cancers, possesses antitumor activity attributed to its induction of specific double-strand DNA breaks (27, 39). Antigen processing and presentation in the MHC-I context is a complex, multistep process subject to regulation at multiple levels (48). Previous studies have highlighted the involvement of the NF-κB and cGAS/STING pathways in cancer-related MHC-I expression (7, 49). Our results reveal that BLM treatment induces substantial DNA damage and promotes CD8^+^ T cell activation through the specific upregulation of MHC-I expression in an ATM/ATR–NF-κB–dependent manner. The dominant role of NF-κB in BLM-induced MHC-I upregulation reinforces the significance of the ATM/ATR–NF-κB pathway in regulating MHC-I expression in tumors. However, the specific molecular target of BLM remained unclear, and its identification is crucial for a comprehensive understanding of its efficacy in MHC-I upregulation.

Building on BLM’s induction of double-strand DNA breaks, the exploration of its synergy with DNA methyltransferase inhibitors in antitumor immunity emerges as a pivotal avenue. The experimental data presented in this study conclusively demonstrate that the combination therapy involving BLM and DNMTi broadens the application potentials of both agents.

The importance of BLM in combination therapy becomes apparent as it showcases the potential to enhance various immunotherapy approaches that rely on T cells as primary effectors. This encompasses checkpoint blockade therapy, bispecific antibody therapy, and TIL therapy for solid-tumor indications. The correlation established between high MHC-I expression and improved antigen presentation serves to validate the heightened efficacy of immunotherapies. Our study further establishes that BLM plays a crucial role in augmenting antitumor responses facilitated by these immunotherapy modalities.

Additionally, alternative pathways beyond MHC-I expression may contribute to the immune sensitivity of BLM-treated tumors, such as activated IFN-α/γ and inflammatory pathways, which may facilitate T cell infiltration. Further research should delve into these alternative pathways to discern their impact on the immunological responsiveness of BLM-treated tumors.

Addressing pulmonary toxicity associated with BLM is imperative for its clinical application. While high doses (15 to 20 mg/kg) result in lung inflammation and pulmonary fibrosis (27, 50), our study indicates that a lower dosage of BLM (3 mg/kg) increases lymphocyte infiltration in tumor samples and remodels the tumor immune microenvironment without causing notable lung injury. This suggests the potential of repurposing BLM at lower doses, making it a more viable and economical option for cancer treatment.

TIL therapy represents an intricately personalized approach to cancer treatment, influenced by a multitude of variables, and often yields unpredictable outcomes. Consequently, the inherent limitations of TIL therapy have spurred researchers to explore novel avenues for enhancing its efficacy. In this study, we reveal that BLM renders patient-derived bladder cancer cells more susceptible to cytotoxicity mediated by autologous TILs. This finding suggests a potential combination therapy to enhance the efficacy of TIL therapy.

This suppression of MHC-I is a critical viral strategy to avoid immune surveillance (51). The downregulation of MHC-I expression is induced by influenza A and B viruses, which hinder viral clearance by CD8^+^ T cells (52). SARS-CoV-2 can inhibit the induction of the MHC class I pathway by targeting the STAT1/IRF1/NLRC5 axis (53). Restoring antigen presentation by IFN-γ can enhance the immune system’s ability to combat viral escape (54). BLM’s ability to induce MHC-I expression could, therefore, provide a dual therapeutic strategy, augmenting the immune response against both cancer cells and virally infected cells, making it a promising candidate for further antiviral research. Interestingly, BLM has been reported to inhibit multiple viruses, including HIV, picornavirus, herpesvirus, and poxvirus (55, 56). This highlights the potential of BLM as an antiviral agent by combating immune evasion. In summary, our data underscore the ability of BLM to augment cytotoxic T cell recognition and responses by inducing surface MHC-I expression in tumor cells. Furthermore, the potential of combination therapy with BLM extends beyond adoptive T cell transfer therapy, encompassing other immunotherapy modalities dependent on T cells as primary effectors. The demonstrated correlation between MHC-I expression and improved immunotherapy efficacy repurposes BLM as a key player in advancing the field of cancer immunotherapy.

## Methods

Additional details for methods are provided in the Supplemental Methods.

### Sex as a biological variable

The mechanisms and pathways investigated are not sex specific in our study. Female mice (C57BL/6) were exclusively used in the animal experiment because of their more docile nature, which leads to more consistent experimental conditions. Our bladder cancer patient cohort included both men and women.

### High-throughput flow cytometry screening system

To facilitate high-throughput screening, SU-DHL-4 cells were seeded in round-bottom, 96-well plates, and FDA-approved drugs were introduced into the cell plates. Additionally, each plate received 500 U/mL IFN-γ (315-05 20 μg, PeproTech) and DMSO for the manual addition of positive and negative controls. After 48 hours, the cells were labeled with the anti–HLA-A/B/C antibody W6/32-APC (311410, BioLegend) at 4°C for 30 minutes. Subsequently, the cell samples were analyzed using the IntelliCyt iQue Screener PLUS (Sartorius).

### Peptide pulsing assay

For the peptide pulsing assay, B16F10 cells were pretreated with BLM at the indicated concentrations or with PBS as a control for specified durations. Following this treatment, the cells were pulsed with 1 ng/mL of SIINFEKL (OVA peptide) at 37°C for 2 hours. The cell surface expression of H-2K^b^-SIINFEKL was assessed using flow cytometry. In the coculture experiments, the culture media were removed, and the cells were washed with PBS to eliminate residual BLM and OVA peptide after pulsing. Subsequently, OT-I CD8^+^ T cells were added at a 2:1 effector/target ratio. After approximately 20 hours of coculture, all cells were harvested and analyzed using flow cytometry. Tumor cells were identified as the CD45-negative population, and the cell apoptosis rate was determined using the Annexin V/633 Apoptosis Detection Kit (AD11, DOJINDO) and analyzed with FlowJo v10.7 Software (BD Biosciences).

### Coculture of cancer cells and T cells for T cell cytotoxicity assay

#### Coculture of mouse tumor cells and OT-I CD8^+^ T cells.

C57BL/6-Tg (TcraTcrb)1100Mjb/J (OT-I) mice were shared by Jianhua Li’s lab from the Department of Pathogen Biology at School of Basic Medical Sciences, Fudan University, Shanghai. B16F10 and B16OVA cells were pretreated with BLM (1.25 μM or 2.5 μM, respectively) or PBS for 24 hours. CD8^+^ T cells were isolated from the spleens and lymph nodes of OT-I mice, stimulated with SIINFEKL (OVA peptide) (HY-P1489A, InvivoGen), and cultured in RPMI 1640 medium containing 10% FBS, 1% penicillin-streptomycin, 2 mM l-glutamine (25030081, Gibco, Thermo Fisher Scientific), 10 mM HEPES (15630080, Gibco, Thermo Fisher Scientific), MEM nonessential amino acids (11140050, Gibco, Thermo Fisher Scientific), 50 μM β-mercaptoethanol (444203, MilliporeSigma), and 10 ng/mL recombinant mouse IL-2 (78081, STEMCELL Technologies). These mouse CD8^+^ T cells were then cocultured with BLM- or PBS-pretreated tumor cells at a ratio of 1:2 (tumor cells/T cells) for approximately 20 hours. At the conclusion of the experiment, the concentration of IFN-γ in the coculture supernatant was determined by ELISA, and the percentage of apoptotic tumor cells was assessed by flow cytometry.

#### Coculture of human tumor cells and human CD8^+^ T cells (bispecific antibody treatment).

SK-BR-3 cells were pretreated with BLM (1.25 μM) or PBS for 24 hours. Peripheral blood mononuclear cells (PBMCs) were collected from healthy donors with informed consent and isolated via density gradient centrifugation using Ficoll Paque Plus (GE Healthcare, now Cytiva, 17144003). CD8^+^ T cells were subsequently purified from PBMCs using negative selection with the EasySep Human CD8^+^ T Cell Enrichment Kit (19053, STEMCELL Technologies) following the manufacturer’s protocol. Human CD8^+^ T cells were stimulated in 12-well culture plates coated with 1 μg/mL anti-human CD3 antibody (300402, BioLegend) and 2 μg/mL soluble anti-human CD28 antibody (302902, BioLegend) along with 10 ng/mL recombinant human IL-2 (200-02-1MG, PeproTech, Thermo Fisher Scientific). Human CD8^+^ T cells were cultured in RPMI 1640 medium containing 10% FBS, 1% penicillin-streptomycin, 2 mM l-glutamine, 10 mM HEPES, MEM nonessential amino acids, and 50 μM β-mercaptoethanol. Coculture experiments involving human CD8^+^ T cells and BLM- or PBS-pretreated tumor cells at a ratio of 1:2 (tumor cells/T cells) in the presence of the appropriate bispecific antibody (0.3 nM H2-scDb) for approximately 20 hours. At the conclusion of the experiment, the concentration of IFN-γ in the coculture supernatant was measured using ELISA.

#### Coculture of NY-ESO-1^+^ human tumor cells and human CD8^+^ T cells transduced with the NY-ESO-1 TCR.

Human CD8^+^ T cells were engineered to express a recombinant TCR targeting the NY-ESO-1 antigen (specifically the NY-ESO-1:157–165 epitope) in an HLA-A*02-restricted fashion (referred to as ESO T cells). NY-ESO-1^+^ SK-BR-3 cells were constructed by overexpressing NY-ESO-1 gene. NY-ESO-1^+^ SK-BR-3 cells were pretreated with BLM (2.5 μM, 5 μM, 10 μM, and 20 μM) or PBS for 24 hours. ESO T cells were then cocultured with BLM- or PBS-pretreated NY-ESO-1^+^ SK-BR-3 cells at a ratio of 1:2 (tumor cells/T cells) for approximately 20 hours. At the conclusion of the experiment, the percentage of apoptotic tumor cells was assessed by flow cytometry.

### Bispecific antibody production

Bispecific antibody production was performed as previously described (41). Briefly, 1 L FreeStyle 293-F cells (Gibco, Thermo Fisher Scientific) were transfected with 1.2 mg of plasmid DNA using polyethylenimine. After 3 days, the culture medium was collected and filtered through a 0.45 μm filter. Ni-NTA Agarose (30210, QIAGEN) was pre-equilibrated with PBS and incubated overnight with the supernatant. Following this, nonspecifically bound proteins were removed from the agarose by washing with PBS, 10 mM, and 20 mM imidazole before being eluted with 50 mM and 100 mM imidazole. The protein was concentrated and desalted in PBS using Amicon Ultra-15 Centrifugal Filters (UFC901096, MilliporeSigma). The protein concentration was determined using Coomassie blue staining or the Pierce BCA Protein Assay Kit (23225, Thermo Fisher Scientific).

### Adoptive T cell transfer therapy and BrdU labeling

B16OVA cells (2 × 10^5^/mouse) were subcutaneously injected into the right flank of C57BL/6 mice (purchased from Shanghai JieSiJie Laboratory Animal Company Limited, 6–8 weeks old). After 8 days, when the tumor volume reached approximately 100 mm^3^, the mice were randomly assigned to different treatment groups and received either PBS or BLM (3 mg/kg, dissolved in PBS). Tumor size was monitored every 2 days. On the 11th day, preactivated OT-I CD45.1^+^ cells (1 × 10^6^/mouse) were intravenously injected into tumor-bearing mice. For in vivo BrdU labeling of transferred OT-I cells, mice were injected intraperitoneally with 1 mg (0.1 mg/mL) of BrdU (BD Biosciences) in 1× PBS 24 and 48 hours after OT-I transfer. Tumors were harvested and BrdU staining kit was used for the flow cytometry analysis 72 hours after OT-I transfer. For tumor weight and lung toxicity evaluation, all tumor-bearing mice were humanely euthanized, and their tumors and lungs were collected on the 18th day. For survival studies, endpoints included death, mouse weight loss exceeding 20%, significant tumor ulceration, and tumor volume exceeding 2,000 mm^3^. Animal survival rates were recorded daily.

### Expansion of TILs and tumor cell lines from patient-derived bladder tumor samples

Fresh tumor samples were sectioned into small fragments measuring approximately 1–3 mm^3^, which were then placed into a culture medium containing 60,000 IU/mL of IL-2. After 4 weeks of expansion, the resulting TILs were either cryopreserved or subjected to a rapid expansion protocol (REP) involving irradiated human PBMCs as feeder cells. Bladder tumor cell lines were established according to previously established protocols (47).

The established bladder tumor cells were pretreated with BLM or were exposed to PBS for 24 hours. Simultaneously, expanded TILs were harvested after REP and cocultured with BLM-pretreated or PBS-exposed tumor cells at a ratio of 1:4 (tumor cells/TILs) for approximately 20 hours. Cytotoxicity was assessed using CellTiter-Glo reagent (Promega), and the concentration of IFN-γ in the coculture supernatant was determined by ELISA.

### Statistics

Data are represented as mean ± SEM. Differences between 2 groups and among multiple groups were evaluated using a 2-tailed *t* test and 1-way ANOVA, respectively. Each experiment was repeated 3 times independently. Data analyses were conducted using SPSS software. A *P* value less than 0.05 was considered significant.

### Study approval

This study enrolled 8 patients diagnosed with bladder cancer, with 4 having high-grade and 4 having low-grade forms of the disease. The detailed clinical information of the patients is listed in Supplemental Table 7. All experimental procedures were approved by the Zhongshan Hospital Ethics Committee (project numbers: B2016-148 and B2017-129R) and Medical Ethics Committee of the School of Basic Medical Sciences, Fudan University (project number: 2023-C005). Written informed consent was obtained from all the patients. All animal experiments were performed based on the guidelines published by the Association for Assessment and Accreditation of Laboratory Animal Care, and the animal studies were approved by the Department of Laboratory Animal Science, Fudan University.

### Data availability

The single-cell RNA-sequencing data and the bulk RNA-sequencing data in this study have been deposited in the Genome Sequence Archive (57), in National Genomics Data Center (58), and in China National Center for Bioinformation/Beijing Institute of Genomics, Chinese Academy of Sciences and are publicly accessible. The accession numbers are CRA017507 (https://ngdc.cncb.ac.cn/gsa/search?searchTerm=CRA017507) and HRA007881 (https://ngdc.cncb.ac.cn/gsa-human/browse/HRA007881). Values for all data points found in graphs are in the Supporting Data Values file.

## Author contributions

Y Dang, WJ, and WX conceived and supervised the project; QY, Y Dong, XW, and CS conducted the experiments; SJ, Y Dong, CS, and RZ provided biopsy samples and cultured primary cells and TILs; QY analyzed the data; QY wrote the manuscript; and Y Dang and WJ modified the manuscript. All the authors have read and approved the final manuscript.

## Supplementary Material

Supplemental data

Unedited blot and gel images

Supplemental table 1

Supporting data values

## Version 1 08/06/2024

In-Press Preview

## Version 2 09/10/2024

Electronic publication
