# Supplementary material for: Pharmacological induction of MHC-I expression in tumor cells revitalizes T cell antitumor immunity
Source: JCI Insight. 2024 Aug 6;9(17):e177788. doi: 10.1172/jci.insight.177788 (PMC11385079; doi:10.1172/jci.insight.177788)
Supplement: Supplemental data [file jciinsight-9-177788-s237.pdf]

Supplemental text

1. Supplemental Methods

Western blotting

For Western blot analysis, cells were harvested, lysed using radioimmunoprecipitation assay (RIPA) lysis buffer, and boiled at 100°C for 10 min. Cell lysates were separated via SDS-PAGE and subsequently transferred onto nitrocellulose membranes (#66485, Pall) following established protocols. After blocking with 5% non-fat milk in PBS + 0.1% Tween-20 for 1 h, membranes were incubated overnight with primary antibodies. Protein bands were visualized through Odyssey infrared imaging system. (Fluorescence Chemiluminescence Imaging System, Clinx Science, Shanghai, China) after incubation with horseradish peroxidase-conjugated secondary antibodies for 1 h at room temperature.

RNA extraction and quantitative Real Time PCR

Total RNA was extracted using a GeneJET RNA purification KIT (#K0732, ThermoFisher) following the manufacturer's protocol. Subsequently, the extracted RNA (1 μg) was transcribed into cDNA using HiScript II Q RT SuperMix, according to the manufacturer's instructions (Vazyme). Real-time qPCR (RT-qPCR) was performed using AceQ qPCR SYBR Green Master Mix (Vazyme) and gene-specific primers (sequences listed in Supplemental Table 2). Data were normalized to GAPDH and quantified via the $2^{-\Delta\Delta Ct}$ method.

Cell viability and cytotoxicity assay

Primary bladder cancer cell lines were trypsinized, counted, and plated in 96-well plates in triplicate. The cells were then incubated overnight. Adherent cells were treated with BLM or the vehicle control (PBS) for three days. Cell viability and cytotoxicity were determined using CellTiter-Glo reagent (Promega) according to the manufacturer's instructions. The absorbance was measured using an EnSpire Multilabel Reader (PerkinElmer).

ELISAs

We employed the Mouse IFN-γ ELISA Set (#555138, BD Pharmingen) and Human IFN-γ ELISA Set (#555142, BD Pharmingen) to assess IFN-γ concentrations in cell culture supernatants following the manufacturer's instructions. The absorbance was measured using an EnSpire Multilabel Reader (PerkinElmer).

Cell viability and cytotoxicity assay

SK-BR-3 and B16OVA cells underwent treatment with indicated Azacitidine (AZA) or Decitabine (DAC) in complete medium every 24 h for five days. Cells were then trypsinized, counted, and plated in 96-well plates in technical triplicate. They were subsequently incubated overnight. Adherent

cells were subjected to treatment with BLM or a vehicle control (PBS) for three days. Cell viability and cytotoxicity were determined using the CellTiter-Glo reagent (Promega) as per the manufacturer's instructions. Absorbance was measured using the EnSpire Multilabel Reader (PerkinElmer).

RNAi-mediated gene silencing, CRISPR/Cas9-mediated gene knockdown

In siRNA knockdown experiments, three on-target siRNAs (GenePharma), one negative control siRNA, and one GAPDH positive control were used. Cells were transfected with siRNA using Lipofectamine RNAiMAX (13778150, Thermo Fisher Scientific). Total protein was extracted 48 h post-transfection for Western blot analysis.

For CRISPR/Cas9 knockdown, 293T cells were co-transfected with PGMD2G, psPAX2, and sgRNA-expressing lentiCRISPR v2 plasmids. The supernatant was collected at 48 and 72 h post-transfection and used to infect cell lines with 1 μg/mL polybrene before puromycin selection. Knockdown efficiency of sgRNAs was determined via western blotting after three days of puromycin selection, and resistant cells were harvested for functional assays. Sequences of all siRNAs and sgRNAs are in Supplemental Table 3 and 4.

Immunofluorescence Imaging

The cells were fixed with 4% paraformaldehyde for 15 min, permeabilized with 0.4% Triton X-100 in PBS for 10 min, and blocked with 4% BSA in PBS for

30 min, all at room temperature. γH2AX protein was detected using anti-phospho-histone H2A.X (Ser139) antibody and Alexa Fluor® 488 Goat Anti-Mouse IgG secondary antibody (#715-545-150, Jackson ImmunoResearch). Cells were stained with DAPI Fluoromount-G (#0100-20, SouthernBiotech) for 10 min and imaged using EVOS™ M5000 (Invitrogen by Thermo Fisher Scientific).

Flow Cytometry

For flow cytometry, cells were washed with PBS and resuspended in stain buffer (#554657, BD Pharmingen) along with primary conjugated antibodies at 4°C for 30 min. After washing with Stain buffer, samples were analyzed using flow cytometry (CytoFlex S Flow Cytometer, Beckman, USA) and the data were analyzed using FlowJo™ v10.7 Software (BD Life Sciences, USA).

Melanoma and colon cancer mouse models

B16F10 cells (2×10^5/mouse) or MC38 cells (5×10^5/mouse) were subcutaneously inoculated into C57BL/6 mice (purchased from Shanghai JieSiJie Laboratory Animal Company Limited, 6-8 weeks old). For drug administration, BLM (dissolved in saline, intraperitoneal injection) was administered every alternate day at 3 mg/kg. Anti-PD-L1 monoclonal antibody

((#BE0101, clone 10F.9G2, Bio X Cell; dissolved in PBS, intraperitoneal injection) was administered at 200 μg on days 7 and 12. Mouse weight and tumor volume were measured every two days.

Cell lines

SU-DHL-4, T-47D, BT549, and MB49 cell lines were obtained from the Cell Bank of the Chinese Academy of Sciences. The cell lines were maintained in RPMI 1640 medium (#C11875500BT, Gibco, Thermo Fisher). B16F10 and B16OVA cell lines were acquired from the Cell Bank at the Chinese Academy of Sciences and cultured in Dulbecco's Modified Eagle Medium (#10566016, Gibco, Thermo Fisher). The HEK293T cell line was kindly provided by Dengke K. M's Lab at California University, San Francisco, and was cultured in Dulbecco's Modified Eagle Medium (#C1199500BT, Gibco, Thermo Fisher). The MC38 cell line, generously shared by Jun O. Liu's Lab at Johns Hopkins University, Baltimore, and was maintained in RPMI 1640 medium (ATCC modification) (#A1049101, Gibco, Thermo Fisher). The SK-BR-3 cell line was procured from ATCC and cultured in McCoy's 5A medium (ATCC modification) (#16600082, Gibco, Thermo Fisher). The MDA-MB-231 cell line was obtained from the Cell Bank of the Chinese Academy of Sciences and cultured in Leibovitz's L-15 (#11415064, Gibco, Thermo Fisher). All culture media were supplemented with 10% fetal bovine serum (FBS) and 1% penicillin/streptomycin. The cells were maintained in 95% humidified air with

5% CO_2 at 37°C.

Tumor-infiltrating cell analysis

At the conclusion of the mouse experiment, tumor samples were collected and digested with collagenase I (C0130, Sigma). The digested tumor cells were then filtered through a 70-μm cell strainer to obtain single-cell suspensions. These cells were subsequently lysed with Red Blood Cell Lysis Buffer (#B541001, Sangon Biotech, Shanghai) to remove erythrocytes. Tumor cells were washed with staining buffer and subjected to flow cytometric staining.

Reagents and antibodies

The chemicals used in this study were bleomycin sulfate (#T6116, Topscience), BAY11-7082 (#HY-13453, MedChemExpress), KU60019 (#HY-12061, MedChemExpress), AZD6738 (#HY-19323, MedChemExpress), and NU7441 (#S2638, Selleckchem). For Mouse T cell activation, OVA Peptide (257-264) TFA were procured from MedChemExpress, and Mouse Recombinant IL-2 was purchased from STEMCELL Technologies. For human T cell activation, anti-human CD3ε (clone UCHT-1) and anti-human CD28 (clone CD28.2) antibodies were obtained from BioLegend. Human IL-2 Recombinant Protein was acquired from Thermo Fisher Scientific (200-02-1MG, PeproTech®). The antibodies used in this study were listed in Supplemental Table 5 and 6.

CCLE Analysis

We retrieved RNA-Seq data from the DepMap Portal, which contains information on 1,406 cancer cell lines. This dataset provides processed gene expression data quantified in accordance with the Genotype-Tissue Expression (GTEx) pipelines.

TCGA Data Analysis

Transcriptomic profiles of tumor data across all TCGA datasets (33 cancer types) were obtained using UCSCXenaTools. Gene expression levels were quantified as logarithmic Transcripts Per Kilobase Million (TPM). The IOBR package facilitated the selection of specific pathways, with correlations between the BLM signature score and the indicated pathway scores assessed using Spearman correlations (1).

Bulk RNA-seq Analysis

For comparative analysis of transcription profiles between control and BLM-treated SU-DHL-4 cells, SU-DHL-4 cells were exposed to either vehicle control or 10 μM BLM for 24 h. Total RNA was isolated according to the manufacturer's protocol, and samples from each experiment were submitted to Novogene, Inc. for sequencing. Illumina HiSeq 2000 was employed for paired-end 150 bp sequencing, with subsequent mapping of sequencing reads

to the hg38 genome using hisat2. Differentially expressed genes were statistically analyzed using DESeq2.

Single-cell RNA-sequencing

Single-cell RNA sample collection and sequencing B16F10 cells (2 × 10^5/mouse) were subcutaneously inoculated into C57BL/6 mice (purchased from Shanghai JieSiJie Laboratory Animal Company Limited, 6-8 weeks old). For drug administration, BLM (dissolved in saline, intraperitoneal injection) was administered three times every other day at 3 mg/kg. Fresh melanoma tumor samples were collected from the tumor-bearing mice. Each group contained three mice, and the single cells were mixed and regarded as one sample for further experiments. The scRNA-seq libraries were constructed using the GEXSCOPE® Single-Cell RNA Library Kit (Singleron Biotechnologies) and Singleron Matrix® Automated single-cell processing system (Singleron Biotechnologies), according to the manufacturer's protocol. Libraries were sequenced on an Illumina Novaseq 6000 with 150 bp paired-end reads.

Preprocessing, filtering, and normalization. Subsequent analyses were performed using "Seurat v4" (2), Single-cell gene expression data of all samples were merged, and transcriptomes were filtered for cells with 500-55,000 genes detected, 1000-20,000 UMIs counted, fraction of mitochondrial reads < 30%, and fraction of hemoglobin reads < 5%. After

filtering, UMI counts were variance-stabilized using scTransform (3) with 3000 variable features, while regressing out the number of UMIs and the fraction of mitochondrial reads.

Functional analysis. InferCNV (v1.16.0) was used for copy number assignment. Differentiation states were predicted using the R package "CytoTRACE" (v 0.3.3) (4). GO function enrichment analysis for melanoma subclusters was performed using the R package "clusterProfiler" (v 4.2.2) (5). Cell-cell communication analysis was performed using the R package "CellChat" (v 1.6.1) (6). Single-cell pseudotime trajectories were computed using R package "monocle3" (v1.0.0) (7). High dimensional weighted gene co-expression network analysis (hdWGCNA) was used to construct a scale-free network at single cell level by R package "hdWGCNA" (v 0.2.24) (8, 9). Gene set functional analyses of a single module were conducted with the R package "enrichR" (v 3.2) (10-12). Oncogenic signaling pathway activity scores were calculated using the R package "progeny" (v 1.16.0) (13, 14).

2. Supplemental Figures

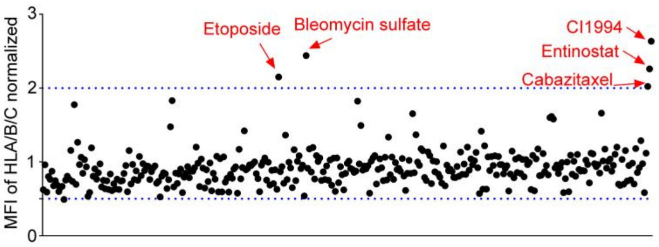

Supplemental Figure 1. HTFCS system was used to identify the candidate drugs for promoting MHC-I expression. SU-DHL-4 were plated in round-bottom 96-well plates. FDA-approved drugs, 500 U/ml IFN-γ, and DMSO were added to the cell plates, respectively. After 48 h, cells were stained with anti-HLA-A/B/C antibody W6/32-APC and analyzed by IntelliCyt iQue Screener PLUS. Dot plot showing the results of HTFCS using an FDA-approved drugs library. Red dots indicate cabazitaxel, etoposide, entinostat, CI994, and Bleomycin sulfate.

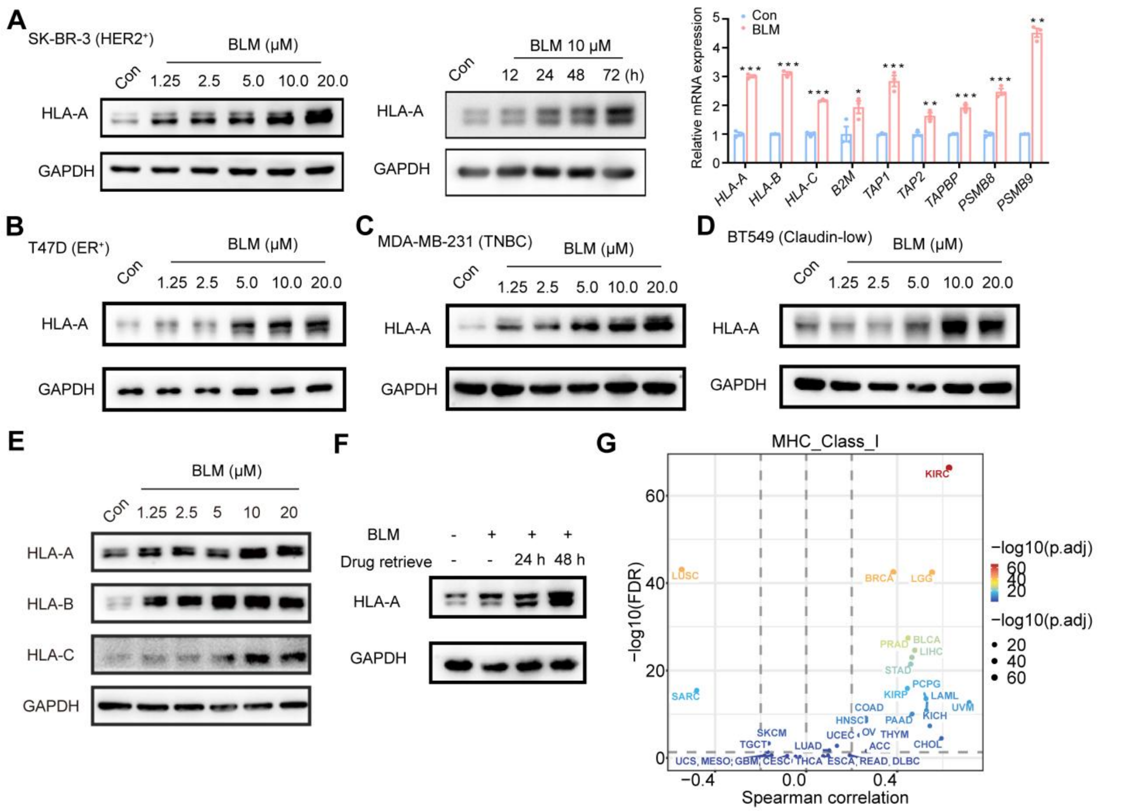

Supplemental Figure 2. BLM promoted MHC-I expression in different types of tumor cells. (**A**) Western blot analysis of HLA-A expression in SK-BR-3 cells after the indicated BLM concentrations or BLM treatment times. qRT-PCR analysis of the antigen presentation gene expression after BLM treatment for 48 h. (**B-D**) Western blot analysis of HLA-A expression in T-47D (**B**), MDA-MB-231 (**C**) and BT549 (**D**) cells after the indicated BLM treatment concentrations. (**E**) Western Blot analysis of the HLA-A, HLA-B, HLA-C expression in SK-BR-3 cells after the indicated BLM concentrations for 48 h. (**F**) SK-BR-3 cells pretreated with BLM (10 μM) for 48 h had their media removed. Cells were then washed with PBS to remove remaining drugs. Following 24 h or 48 h, protein samples were collected. (**G**) TCGA RNA-seq data analysis

reveals the correlation of BLM-treated signature with MHC Class I pathways. Plots display Spearman correlation and estimated statistical significance for the indicated pathway among different human cancer types. Each dot represents a human cancer type in TCGA. Data are shown as mean ± SD. *$P < 0.05$, **$P < 0.01$, and ***$P < 0.001$ compared to the vehicle group by unpaired t test.

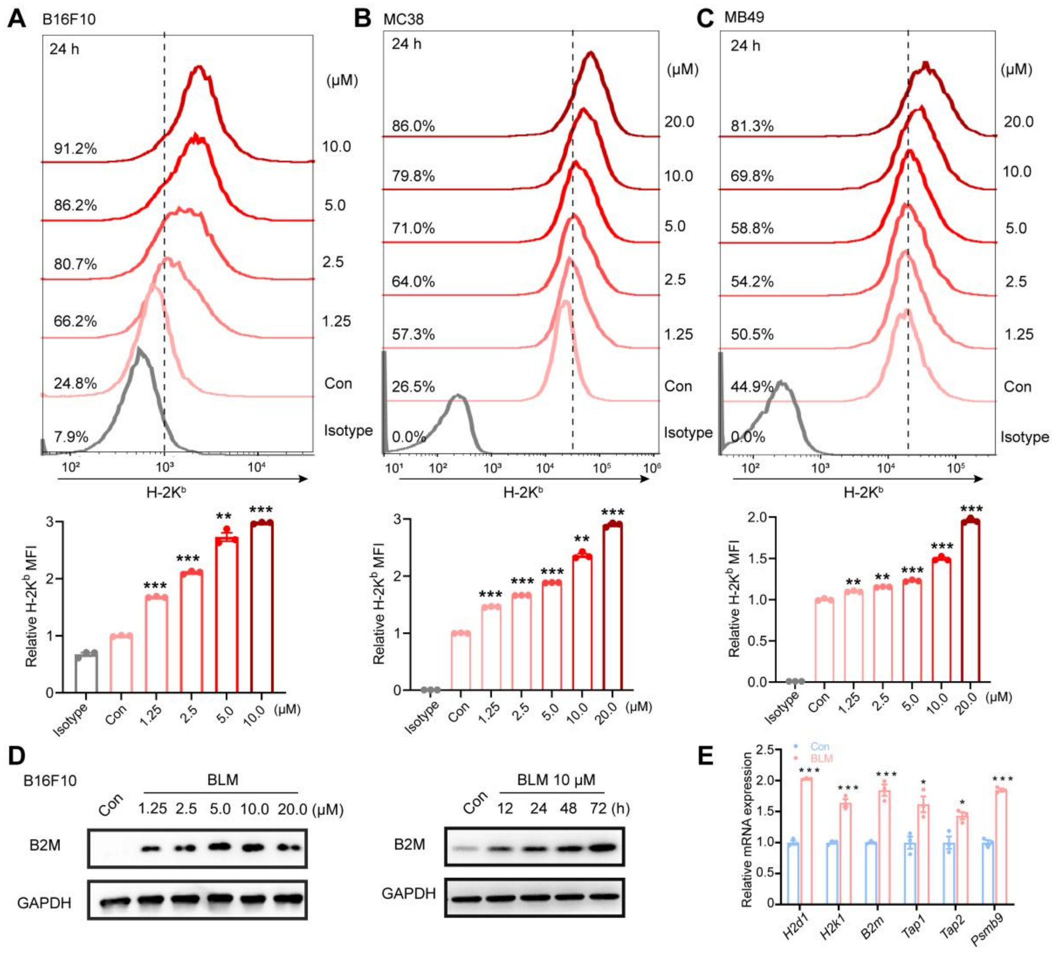

Supplemental Figure 3. Antigen presentation pathway was activated after BLM treatment. (**A**-**C**) Cell surface H-2K^b in B16F10 (**A**), MC38 (**B**), and MB49 (**C**) cell lines incubated with the indicated concentrations of BLM for 24 h (up panel). Quantification of mean fluorescence intensities (MFI) of H-2K^b from (**A-C**), n=3 per group (down panel). (**D**) Western blot analysis of B2M expression in B16F10 cells after the indicated BLM concentrations or BLM treatment times. (**E**) qRT-PCR analysis of the antigen presentation gene expression in B16F10 cells after BLM treatment for 48 h. Data are shown as mean ± SD. **P* < 0.05, ***P* < 0.01, and ****P* < 0.001 compared to the vehicle group by one-way ANOVA (**A**-**C**) and unpaired t test (**E**).

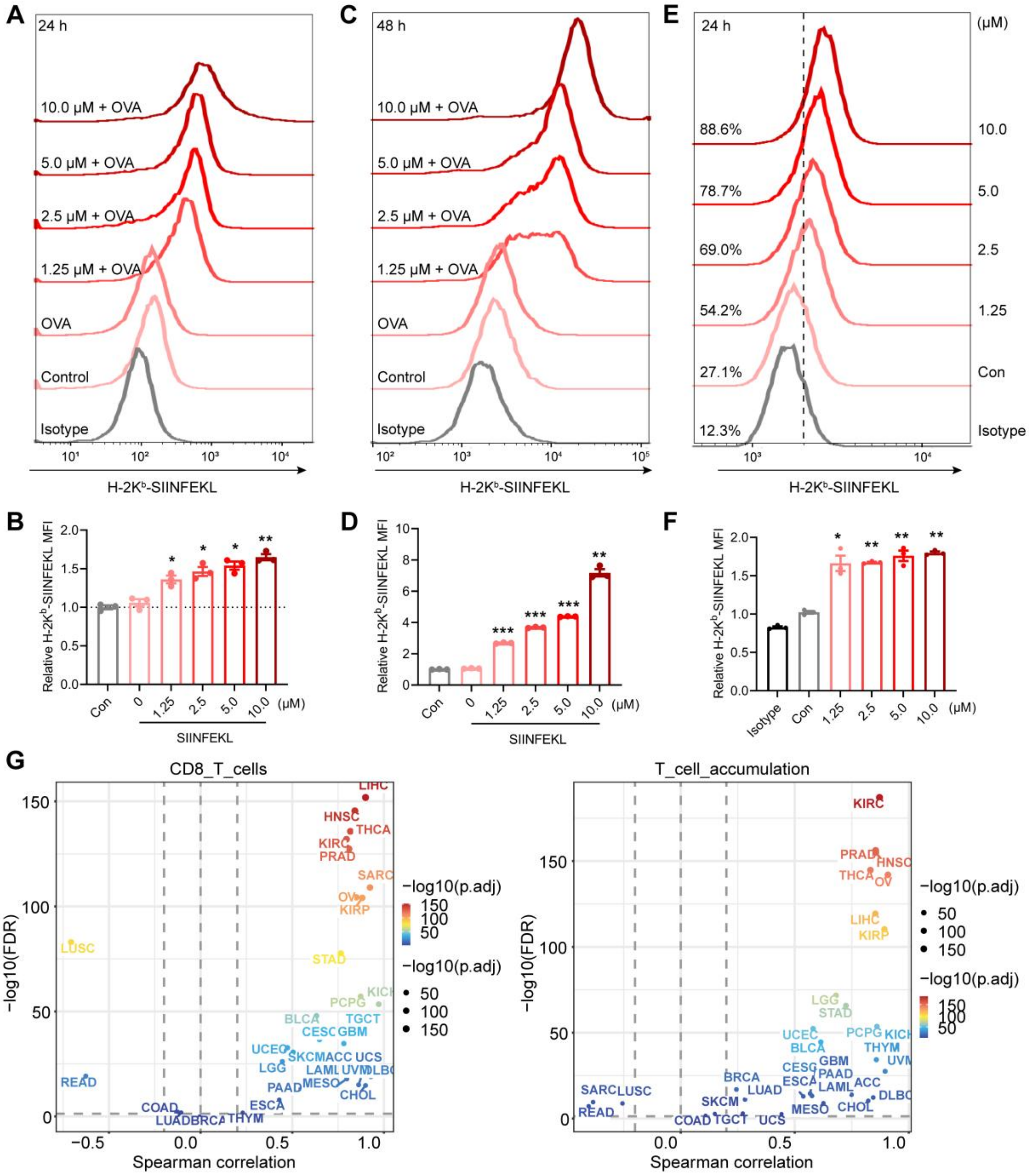

Supplemental Figure 4. BLM treatment promoted peptide-MHC-I complex expression in B16F10 cells. (**A**) Cell surface H-2K^{b}-SIINFEKL in B16F10 cells treated with OVA peptide for two hours following pretreatment with the indicated concentrations of BLM for 24 h. (B) Quantification of mean fluorescence intensities (MFI) of H-2K^{b}-SIINFEKL from (**A**), n=3 per group. (C) Cell surface H-2K^{b}-SIINFEKL in B16F10 cells treated with OVA peptide for two hours following pretreatment with the indicated concentrations of BLM for 48 h.

(D) Quantification of mean fluorescence intensities (MFI) of H-2K^b-SIINFEKL from (C), n=3 per group. (E) Cell surface H-2K^b-SIINFEKL in B16OVA cells treated with the indicated concentrations of BLM for 24 h. (F) Quantification of mean fluorescence intensities (MFI) of H-2K^b-SIINFEKL from (E), n=3 per group. (G) TCGA RNA-seq data analysis revealed the correlation of BLM-treated signature with CD8$^+$ T cell pathway or T cell accumulation pathway. Plots display Spearman correlation and estimated statistical significance for indicated pathways among different human cancer types. Each dot represents a human cancer type in TCGA. Data are shown as mean ± SD. *P < 0.05, **P < 0.01, and ***P < 0.001 compared to the vehicle group by one-way ANOVA.

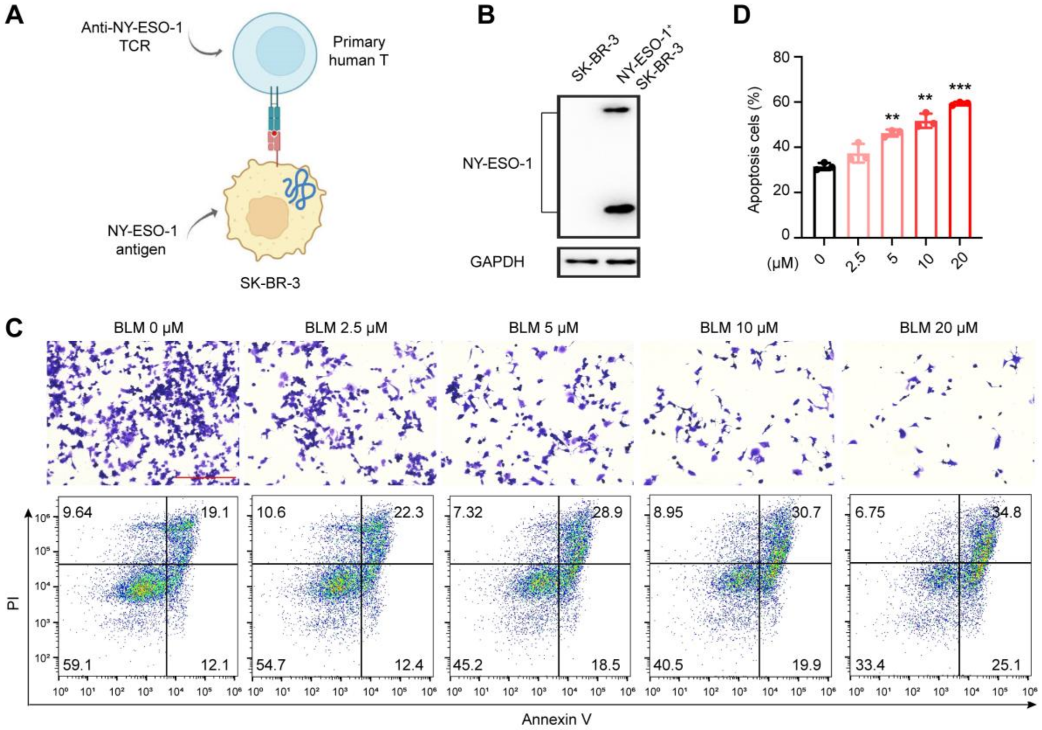

Supplemental Figure 5. BLM enhanced the cytotoxity effect of human CD8⁺ T cells on SK-BR-3 cells. (**A**) Schemics of the co-culture assay of human tumor cells and CD8⁺ T cells. Briefly, human CD8⁺ T cells were genetically modified to express a specific T cell receptor (TCR) that recognized the NY-ESO-1 antigen presented by SK-BR-3 cancer cells in an HLA-A*02-restricted manner, allowing the engineered T cells to target the cancer cells. (B) Western blot analysis of NY-ESO-1 expression in SK-BR-3 cells transfected with NY-ESO-1 antigen. (**C**) Co-culture assay of human cancer cells and primary human T cells for T cell cytotoxicity assay. NY-ESO-1⁺ SK-BR-3 cells were pre-treated with indicated concentrations of BLM for 24 h prior to co-culture with T cells. The first lane displays the crystal violet staining images of remaining cancer cells (Scale bars, 400 μm). The second lane presents the representative images of cancer cells apoptosis after

co-culture with T cells. (**D**) Quantification of the percentages of early and late apoptotic cells among cancer cells from (**C**), n=3 per group. Data are shown as mean ± SD. **P < 0.01 and ***P < 0.001 compared to the vehicle group by one-way ANOVA.

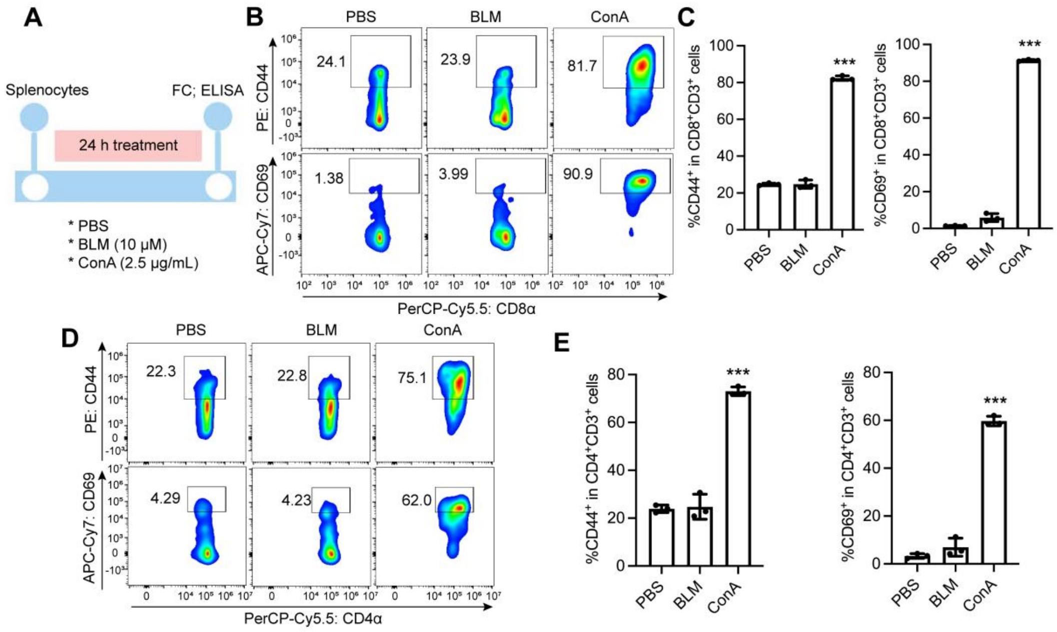

Supplemental Figure 6. BLM enhanced CD8$^+$ T cell activation without antigen-independent activation of T cells. (A) Schematic of an *ex vivo* splenocyte assay. Splenocytes were incubated with BLM or Concanavalin A (Con A) for 24 h. (**B** and **C**) Flow cytometry analysis of the frequencies of CD44$^+$ and CD69$^+$ cells among CD8$^+$ T cells in splenocytes upon indicated treatments; representative images of (**B**) and quantitative data from (**C**) are given, n=3 per group. (**D** and **E**) Flow cytometry analysis of the frequencies of CD44$^+$ and CD69$^+$ cells among CD4$^+$ T cells in splenocytes upon indicated treatments; representative images of (**D**) and quantitative data from (**E**) are given, n=3 per group. Data are shown as mean ± SD. ***$P < 0.001$ compared to the vehicle group by one-way ANOVA.

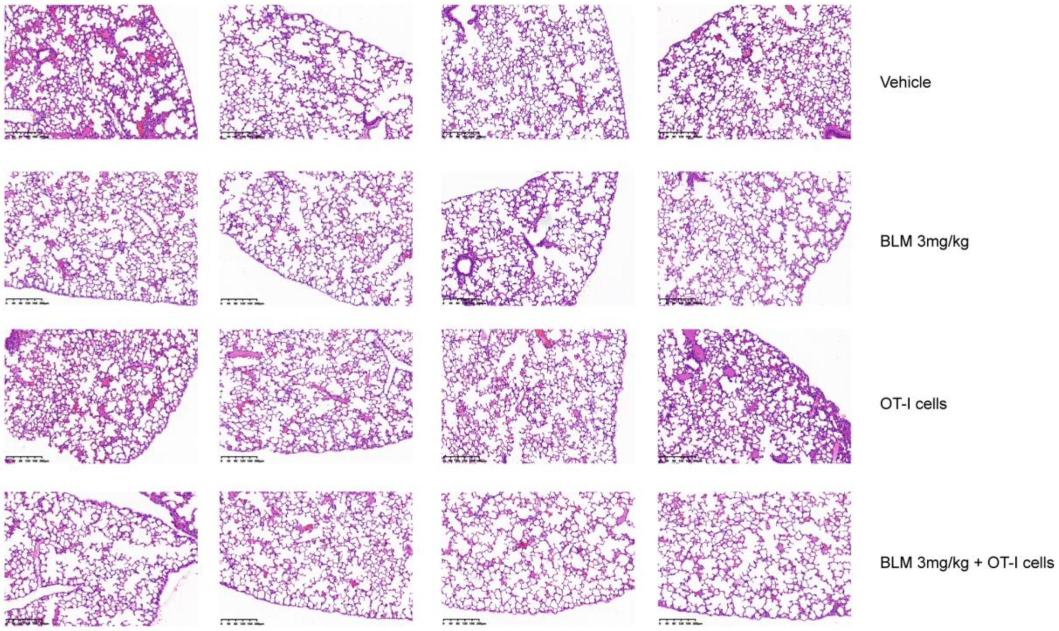

Supplemental Figure 7. BLM treatment exerted no lung toxicity. Representative histology with hematoxylin and eosin (H&E) staining of mouse lungs after treatment with BLM, OT-I cells alone, or their combination. Scale bars: 200 μm.

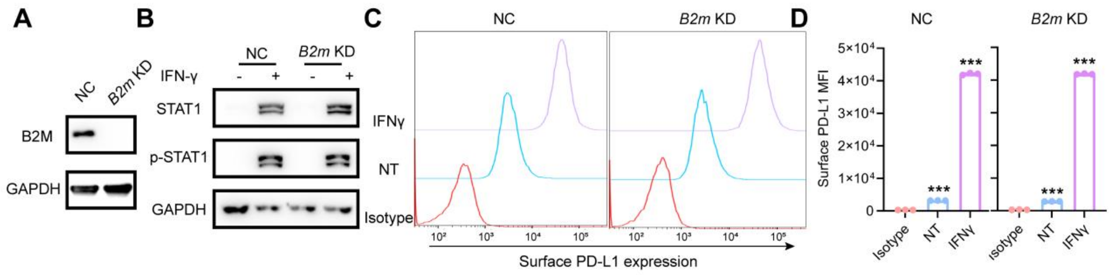

Supplemental Figure 8. Loss of *B2m* in B16OVA cells had no impact on IFN-γ signaling. (A) Western blot analysis of the B2M expression in non-targeting control (NC) or *B2m* knockdown B16OVA cells. (**B**) Western blot analysis of the protein levels of STAT1 and p-STAT1 in non-targeting control (NC) or *B2m* knockdown B16OVA cells incubated with the IFN-γ (10 ng/ml) for 24 h. (**C**) Flow cytometry detected cell surface PD-L1 expression in non-targeting control (NC) or *B2m* knockdown B16OVA cells incubated with the IFN-γ (1 ng/ml) for 24 h. (**D**) Quantification of mean fluorescence intensities (MFI) of PD-L1 from (**C**), n=3 per group. Data are shown as mean ± SD. ***$P <$ 0.001 compared to the vehicle group by one-way ANOVA.

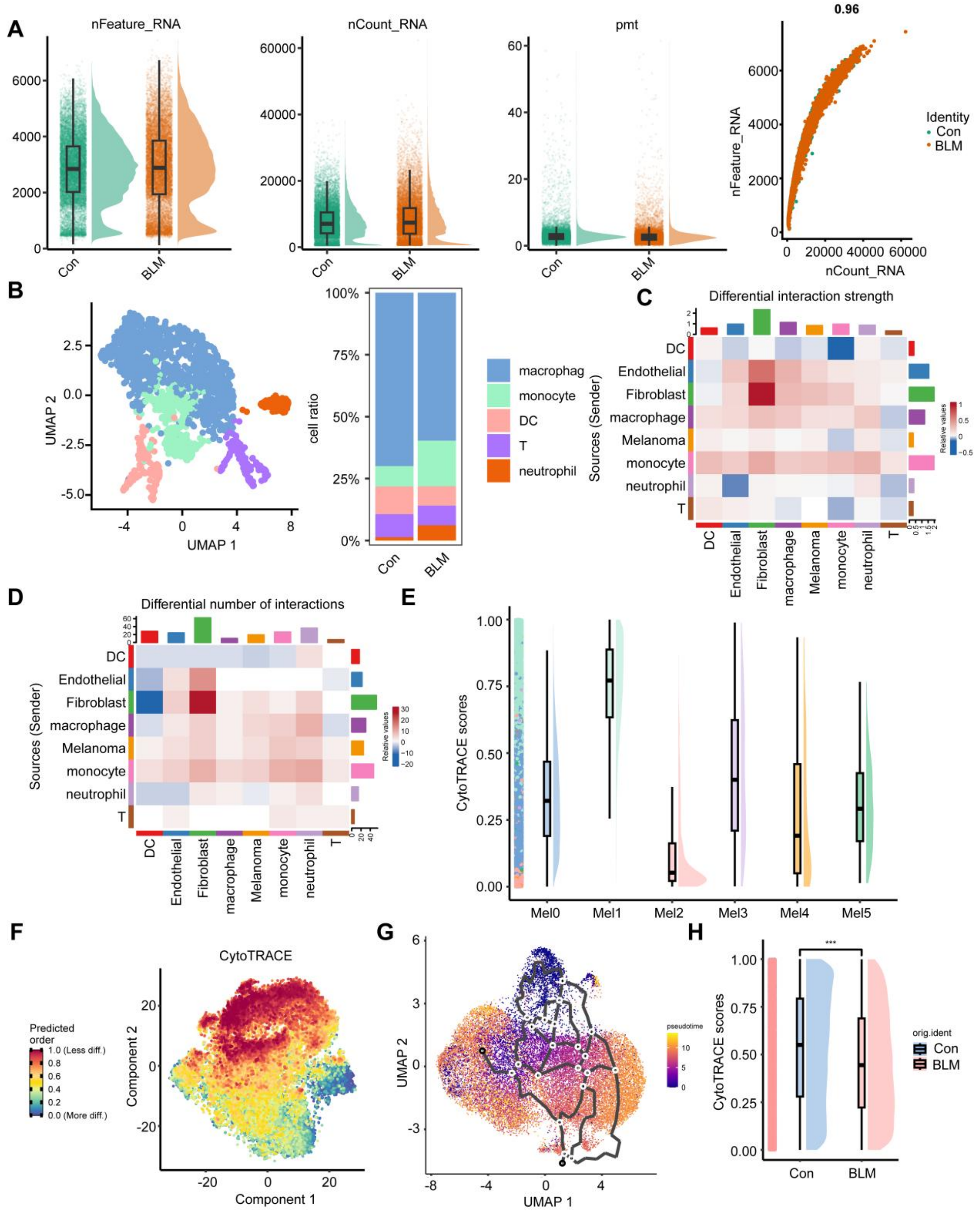

Supplemental Figure 9. Intercellular interactions in melanoma tumors were enhanced after BLM treatment. (**A**) Plots of quality control parameters of all single-cell transcriptomes analyzed in the study. (**B)** UMAP based on the top 20 principal components of all immune single-cell transcriptomes color-coded by cell cluster, and proportion of cell cluster per tumor sample. (**C**

and **D)** Heatmaps of the interaction strength (**C**) and the interaction numbers (**D**) between control and BLM tumor samples. Blue color indicated an increase in the displayed communication in BLM compared to control, whereas the red color indicated a decrease. (**E**) The CytoTRACE scores were compared among melanoma subclusters (Mel0-Mel5). (**F**) The differentiation state of melanoma cells by CytoTRACE analysis. The color showed the state of differentiation; from less differentiated (red) to more differentiated (blue) states. (**G**) Pseudotime trajectory analysis among melanoma subclusters (Mel0-Mel5) was computed by monocle3 package. (**H**) The CytoTRACE scores were compared between control and BLM group. Data are shown as mean ± SD. ***$P < 0.001$ compared to the vehicle group by unpaired t test.

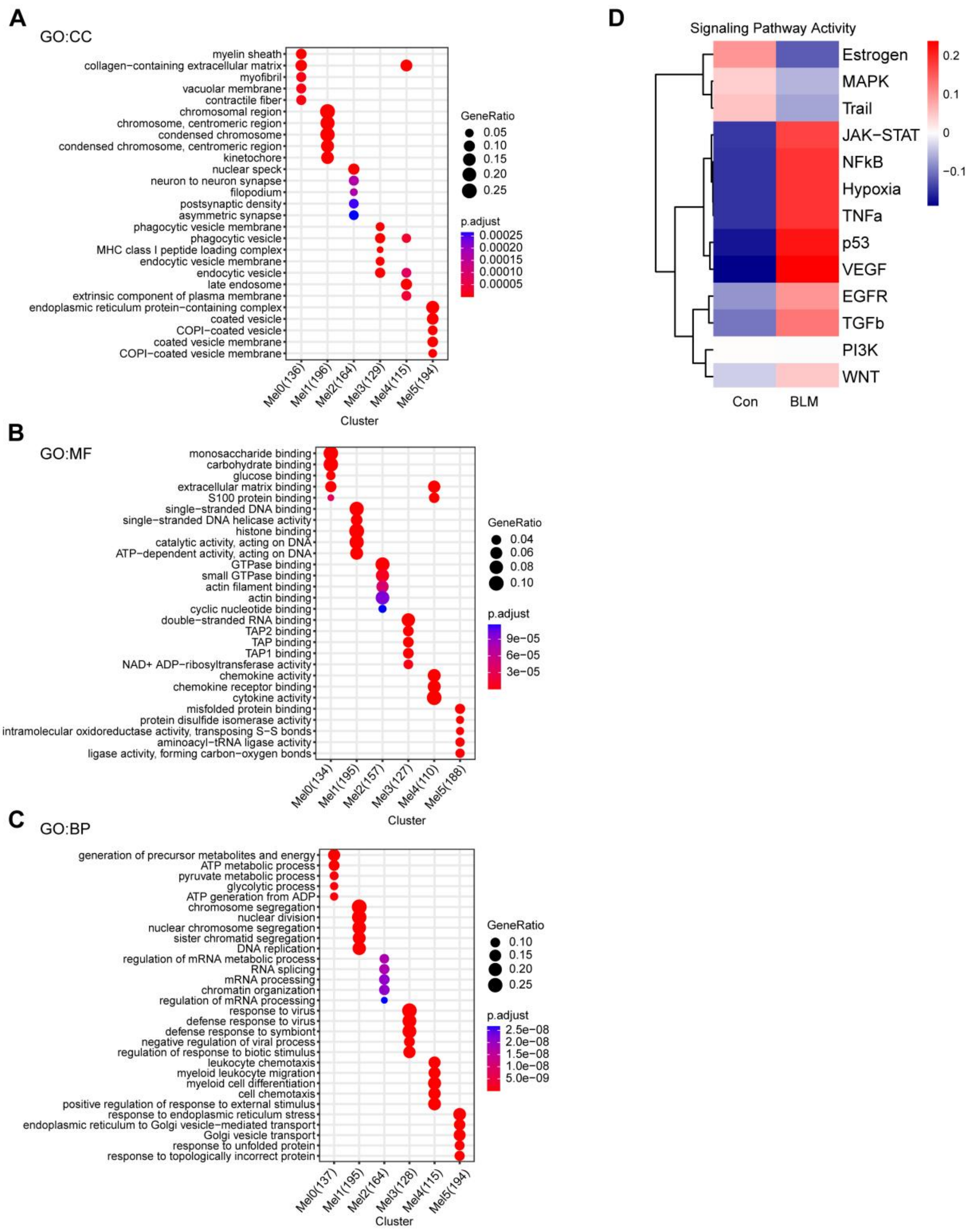

Supplemental Figure 10. Intertumoral heterogeneity of melanoma cells. (**A-C**) Functional enrichment of the melanoma subclusters by gene ontology (GO), including Cellular Component (CC) (**A**), Molecular Function (MF) (**B**) and Biological Process (BP) (**C**). (**D**) Mean pathway activity scores of melanoma tumor cells among different groups.

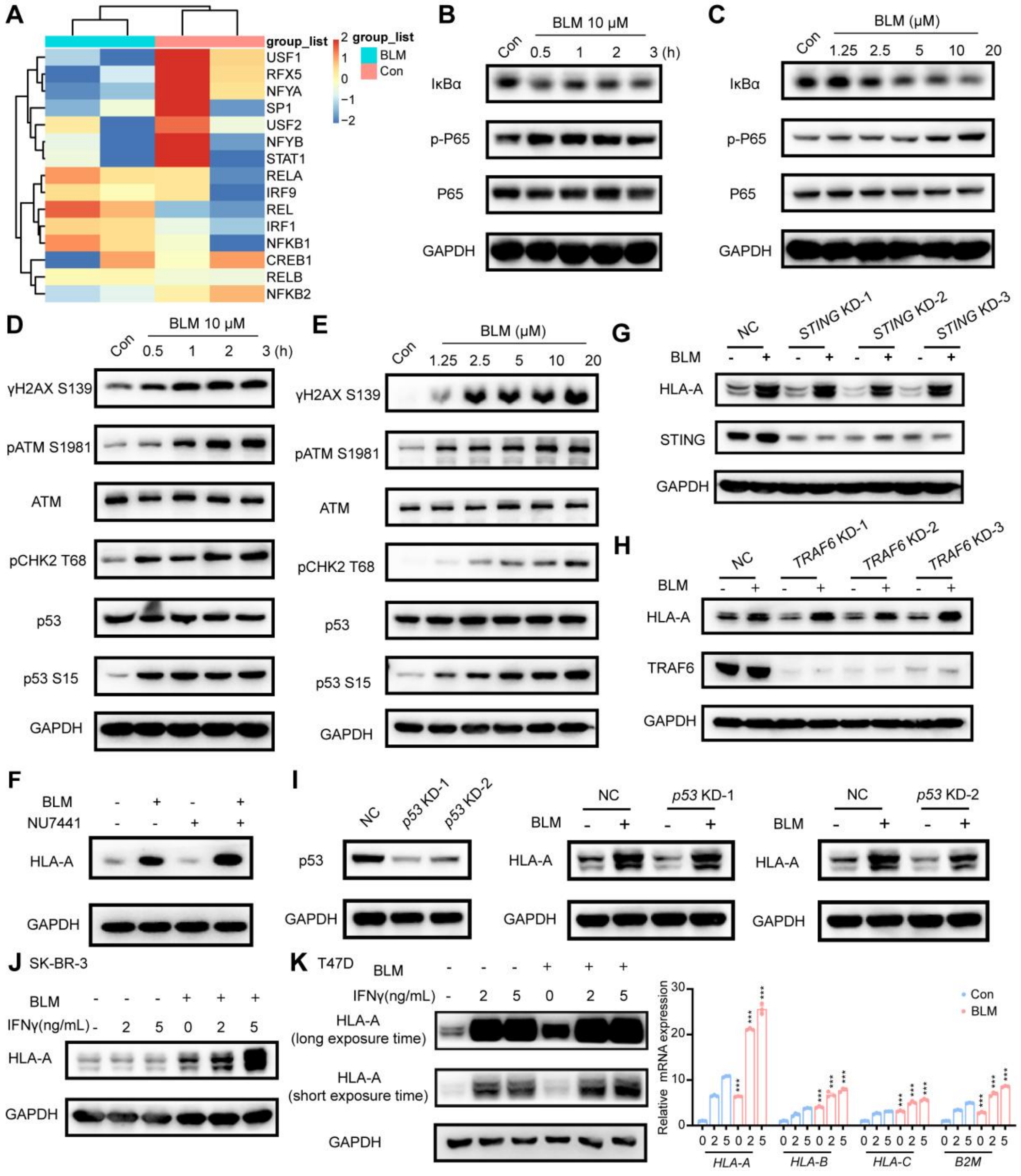

Supplemental Figure 11. STING/p53 pathway was dispensable for MHC-I upregulation. (**A**) Heatmap showing the activity of transcriptional regulators of MHC-I using DoRothEA in SU-DHL-4 cells treated with 10 μM BLM compared with vehicle group for 24 h. (**B**) Western blot analysis of the protein levels of IκBα, p-P65, and P65 in SK-BR-3 cells after the indicated BLM treatment times. (**C**) Western blot analysis of the protein levels of IκBα, p-P65, and P65 in SK-BR-3 cells after the indicated BLM concentrations. (**D**) Western blot

analysis of levels of γH2AX S139, pATM S1981, ATM, pChk2 T68, p53, and p53 S15 in SK-BR-3 cells. SK-BR-3 cells were harvested after treatment with BLM for indicated times. (**E)** Western blot analysis of levels of γH2AX S139, pATM S1981, ATM, pChk2 T68, p53, and p53 S15 in SK-BR-3 cells. SK-BR-3 cells were harvested after treatment with BLM for indicated concentrations. (**F)** Western blot analysis of the HLA-A expression in SK-BR-3 cells. Cells were pre-treated with 10 μM NU7441 for 6 h, then followed with 10 μM BLM for 12 h. (**G**) Western blot analysis of the expression of HLA-A and STING proteins in *STING* depleted SK-BR-3 cells after incubation with BLM for 48 h. (**H**) Western blot analysis of the expression of HLA-A and TRAF6 proteins in TRAF6 depleted SK-BR-3 cells after BLM treatment for 48 h. (**I**) Western blot analysis of the expression of HLA-A and p53 proteins in *TP53* knockdown SK-BR-3 cells after incubation with BLM for 48 h. The knockdown efficiency is shown in the left panel. **(J** and **K)** Western blot and qRT-PCR analysis of MHC-I expression levels in, SK-BR-3 (**J**), and T47D (**K**) cells after BLM treatment in the presence of 2 or 5 ng/ml IFN-γ. Data are shown as mean ± SD. ***$P <$ 0.001 compared to the vehicle group by one-way ANOVA.

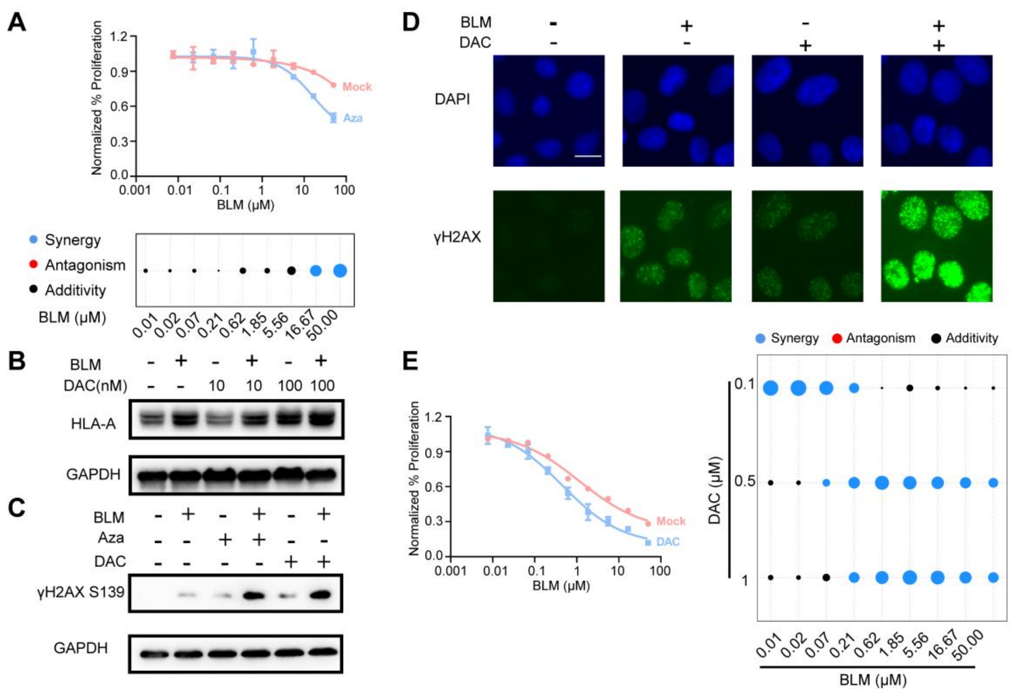

Supplemental Figure 12. DNMTi synergized with BLM for reducing cell proliferation. (**A**) Top panel: Growth inhibition as detected by cell viability of SK-BR-3 cells treated with mock/500 nM Aza for five days and BLM for three days. Bottom panel: Combination index (CI) plots. (**B**) Western blot analysis of the HLA-A expression in SK-BR-3 cells treated with the indicated concentrations of DAC for five days and BLM for two days. (C) Western blot analysis of the γH2AX S139 expression in SK-BR-3 cells treated with Aza (500 nM) or DAC (100 nM) for five days and BLM for one hour. (**D**) Immunofluorescence analysis of dsDNA damage by γH2AX S139 antibody staining after incubation with BLM for one hour prior to exposure to DAC (100 nM) for five days. Scale bars: 25 μm. (**E**) Left panel: Growth inhibition as detected by cell viability of B16F10 cells treated with mock/100 nM DAC for five days and BLM for two days. Right panel: Combination index (CI) plots. Data are shown as mean ± SD.

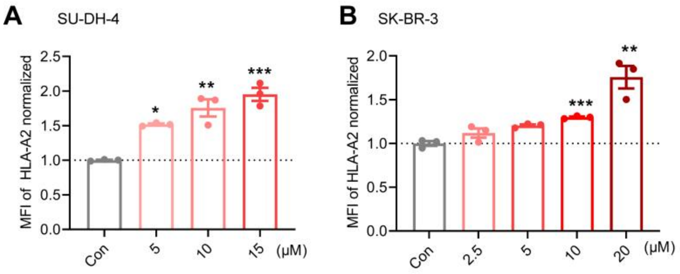

Supplemental Figure 13. BLM promoted HLA-A2 expression. (A and **B)** Cell surface HLA-A2 in SU-DHL-4 (**A**) and SK-BR-3 (**B**) cells incubated with the indicated concentrations of BLM for 48 h. Data are shown as mean ± SD. *P < 0.05, **P < 0.01, and ***P < 0.001 compared to the vehicle group by one-way ANOVA.

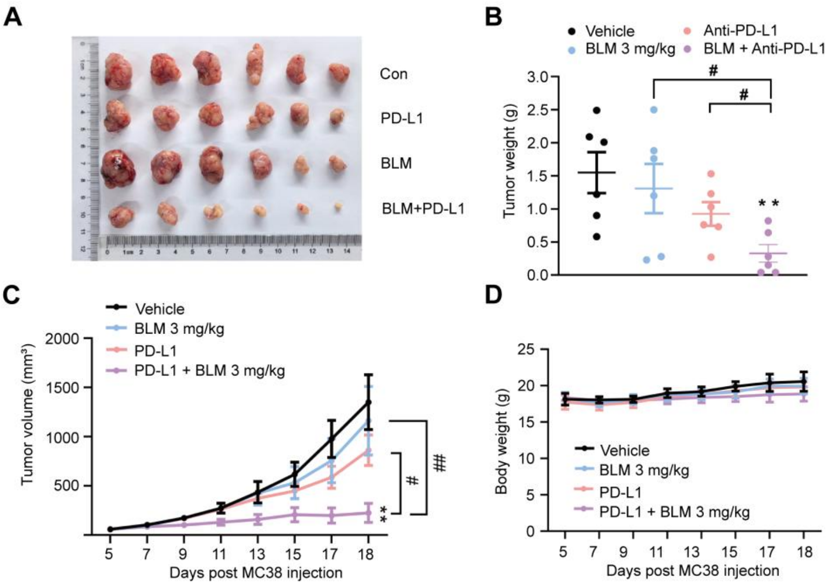

Supplemental Figure 14. BLM enhanced the efficacy of anti-PD-L1 monoclonal antibody treatment in MC38 mouse model. (**A**) The image of tumor tissues at the endpoint. (**B-D**) Treatment of MC38 tumors with BLM or vehicle control in combination with anti-PD-L1 or vehicle control, n=6 per group. Tumor weight (**B**), tumor volume (**C**) and body weight (**D**), n=6 per group. Data are shown as mean ± SD. $^{**}P < 0.01$ compared to the vehicle group by one-way ANOVA; $\#P < 0.05$ and $\#\#P < 0.01$ between the indicated groups by unpaired t test.

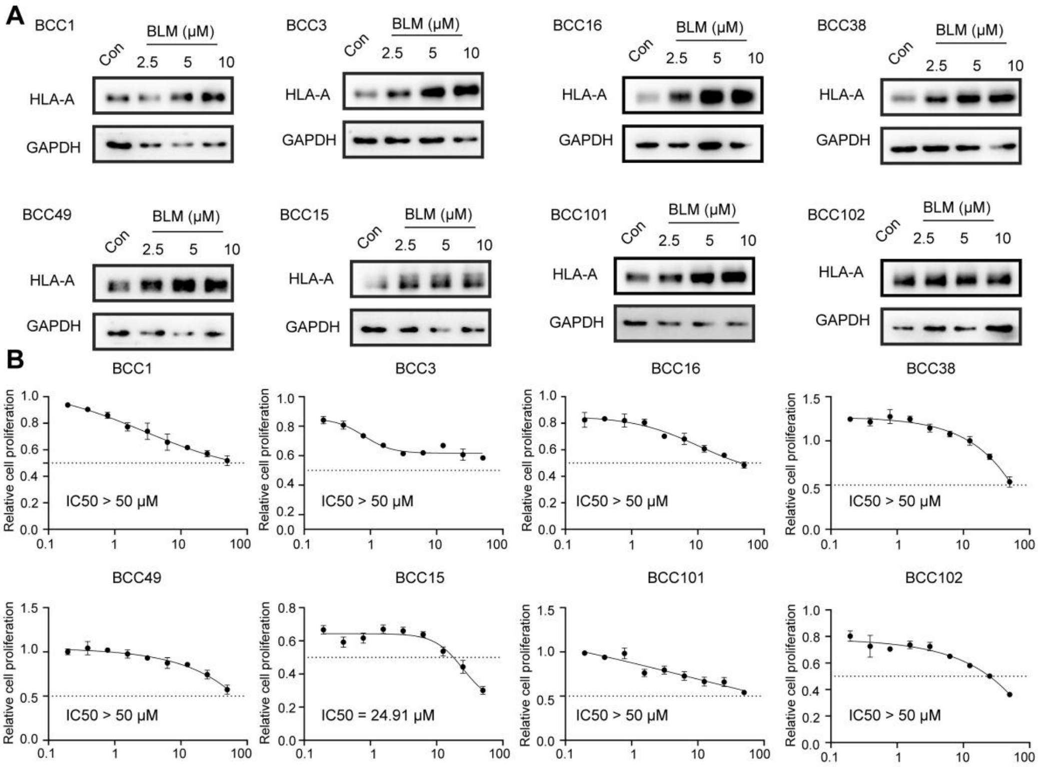

Supplemental Figure 15. The cytotoxicity evaluation of BLM on primary bladder tumor cells. (A) Western blot analysis of the HLA-A expression in urine-derived bladder cancer cells (BCC1, BCC3, BCC16, BCC38, and BCC49) and tumor-derived bladder cancer cells (BCC15, BCC101, and BCC102) treated with indicated concentrations of BLM for 48 h. (B) The cytotoxicity of primary bladder cancer cell lines treated with different concentrations of BLM for 72 h evaluated by half maximal inhibitory concentration (IC50). Data are shown as mean ± SD.

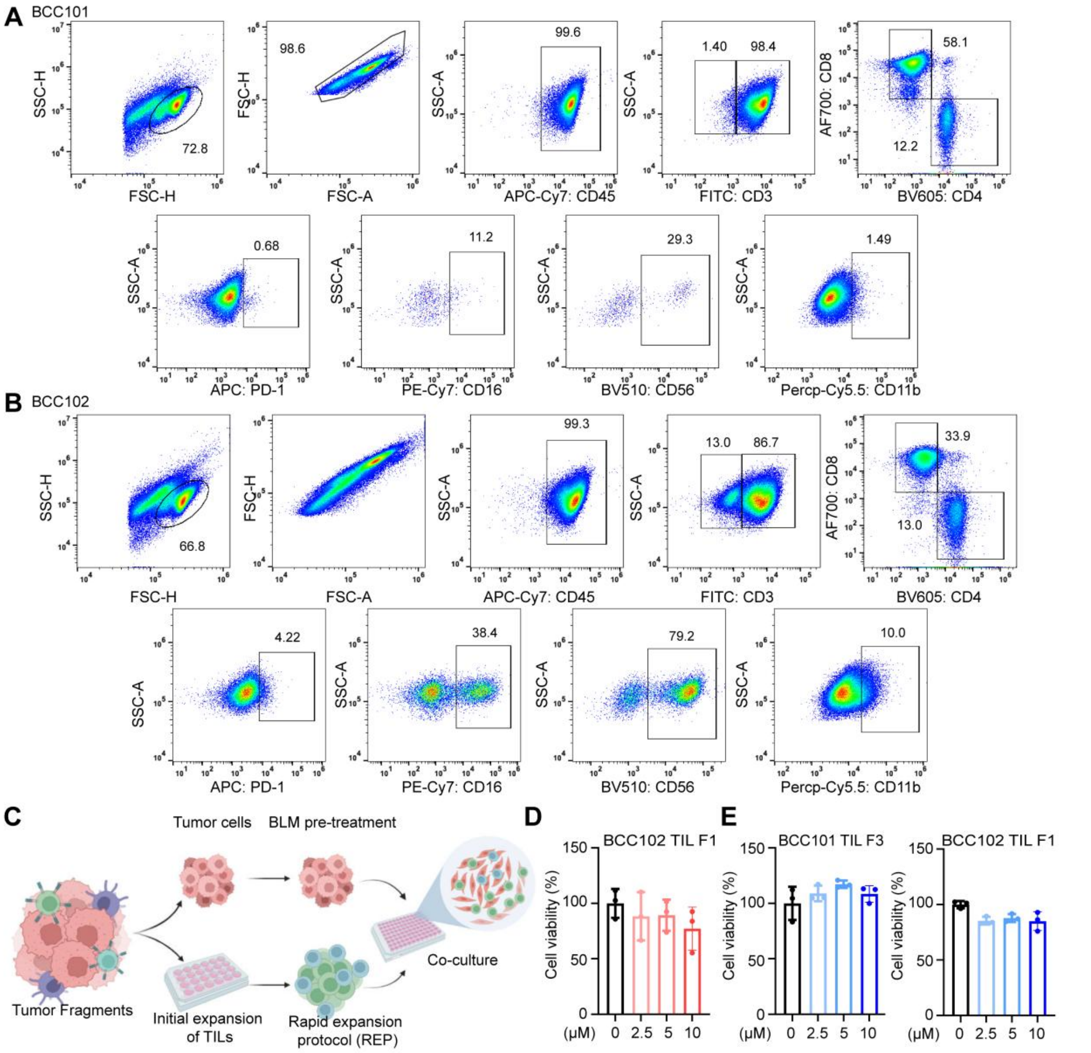

Supplemental Figure 16. Phenotype of TIL expanded from primary bladder tumors. (**A** and **B**) TILs of BCC101 (**A**) and BCC102 (**B**) were collected from each fragment and the percentage of CD45, CD3, CD4, CD8, PD-1, CD16, CD56, and CD11b were measured by flow cytometry four weeks after the initiation of TIL cultures. (**C**) Schematics of the procedure for isolating, amplifying, and co-culturing patient derived tumor cells with autologous TILs. (**D**) Co-culture of primary bladder cancer cells and TILs for T cell cytotoxicity assay. BCC102 were pre-treated with indicated concentrations of BLM for 48 h. Percent remaining live cancer cells following 24 h incubation with autologous

TILs at a 1:5 ratio. (**E**) BCC101 or BCC102 tumor cells were pre-treated with indicated concentrations of BLM for 24 h. Cell viability was measured by the CellTiter-Glo reagent. Data are shown as mean ± SD.

3. Supplemental Tables

Supplemental Table 1. Results of high throughput screening of the FDA-approved drugs. MFI was normalized to IFN-γ-DMSO treated group.

Supplemental Table 2. Primers used in the qRT-PCR study

Gene	Organism	Primer-Forward(5'-3')	Primer-Reverse(5'-3')
HLA-A	human	CTCCAGTGATCACAGCTCCA	CTTCTGGACAGGAGCAGAGA
HLA-B	human	CAGTTCGTGAGGTTCGACAG	CAGCCGTACATGCTCTGGA
HLA-C	human	GGACAAGAGCAGAGATACACG	CAAGGACAGCTAGGACAACC
B2M	human	TGACTTTGTCACAGCCCAAG	AGCAAGCAAGCAGAATTTGG
TAP1	human	CCTGTGGCACAAACTCGGG	ATCTCCCCAAGAGAGGAGAGGA
TAP2	human	TCGACTCACCCTCCTTTCTC	ACTGCATCCTGGATCTCCC
TAPBP	human	CAGCAGGAGCCTGTTCTCAT	AAGCTCAAGTCCAGCAGAGC
PSMB8	human	TCTCCAGAGCTCGCTTT	CACTCCATGCTGGAACT

		ACC	TGA
PSMB9	human	CGTTGTGATGGGTTCTG ATTCC	GACAGCTTGTCAAACAC TCGGTT
GAPDH	human	ACAACTTTGGTATCGTG GAAGG	GCCATCACGCCACAGTT TC
H2k1	mouse	GCTGGTGAAGCAGAGA GACTCAG	GGTGACTTTATCTTCAG GTCTGCT
H2d1	mouse	AGTGGTGCTGCAGAGC ATTACAA	GGTGACTTCACCTTTAG ATCTGGG
B2m	mouse	TGGTGCTTGTCTCACTG ACC	TTCAGTATGTTCGGCTT CCC
Tap1	mouse	AGTCTGGAGCCCACGA TTTCATC	GGGTGATAAGAAGAACC GTCCG
Tap2	mouse	TGCCTGTTCTCTGTTGG GAG	AGCTCCCCTGTCTTGGT CTC
Psmb9	mouse	ACCGTGAGGACTTGTTA GCG	GTAAAGGGCTGTCGAAT TAGCA
Gzmb	mouse	TGTGCTGACTGCTGCTC ACT	TCCTCTTGGCCTTACTC TTC
Prf1	mouse	AACCTCCACTCCACCTT GAC	GTGCGTGCCATAGGAG GAGA
Ifng	mouse	GGATGGTGACATGAAAA	TGCTGATGGCCTGATTG

		TCCTGC	TCTT
Actb	mouse	TTCTTTGCAGCTCCTTCGTT	ATGGAGGGGAATACAGCCC

Supplemental Table 3. Primers used in siRNA study.

Gene	Organism	sense(5'-3')	antisense(5'-3')
si*P65*-1	human	GGAGCACAGAUACCACCAATT	UUGGUGGUAUCUGUGCUCCTT
si*P65*-2	human	CCUUUCUCAUCCCAUCUUUTT	AAAGAUGGGAUGAGAAAGGTT
si*P65*-3	human	GGACAUAUGAGACCUUCAATT	UUGAAGGUCUCAUAUGUCCTT
si*STING*-1	human	CUGGCAUGGUCAUAUUACATT	UGUAAUAUGACCAUGCCAGTT
si*STING*-2	human	GCAUCAAGGAUCGGGUUUATT	UAAACCCGAUCCUUGAUGCTT
si*STING*-3	human	GUGCCUGAUAACCUGAGUATT	UACUCAGGUUAUCAGGCACTT
si*TRAF6*-1	human	GUCCAGUUGACAAUGAAAUTT	AUUUCAUUGUCAACUGGACTT
si*TRAF6*-2	human	GCAGUGCAAUGGAAUU	AUAAAUUCCAUUGCAC

		UAUTT	UGCTT
si*TRAF6*-3	human	GCGCUGUGCAAACUAUAUATT	UAUAUAGUUUGCACAGCGCTT
Negative control	human	UUCUCCGAACGUGUCACGUTT	ACGUGACACGUUCGGAGAATT
GAPDH positive control	human	UGACCUCAACUACAUGGUUTT	AACCAUGUAGUUGAGGUCATT

Supplemental Table 4. Primers used in knockdown study.

Gene	Organism	sense(5'-3')	antisense(5'-3')
B2m	mouse	CACCGCTCGGTGACCCTGGTCTTTC	AAACGAAAGACCAGGGTCACCGAGC
TP53-1	human	CACCGTATCTGAGCAGCGCTCATGG	AAACCCATGAGCGCTGCTCAGATAC
TP53-2	human	CACCGATCTGAGCAGCGCTCATGGT	AAACACCATGAGCGCTGCTCAGATC

Supplemental Table 5. Antibodies used for Western blot analysis.

Antibody Name	Clone Number	Company	Catalog Number

anti-HLA-A	ARC0588	ABclonal	A11406
anti-HLA-B	polyclonal	Proteintech	17260
anti-HLA-C	polyclonal	Proteintech	15777
anti-GAPDH	1E6D9	Proteintech	60004
anti-beta 2 Microglobulin	EP2978Y	Abcam	ab75853
NF-κB p65	L8F6	Cell Signaling Technology	6956
Phospho-NF-κB p65 (Ser536)	93H1	Cell Signaling Technology	3033
anti-IKB alpha	E130	Abcam	ab32518
anti-Phospho-Histone H2A.X (Ser139)	20E3	Cell Signaling Technology	9718
anti-ATM (phosphor S1981)	EP1890Y	Abcam	ab81292
anti-ATM	Y170	Abcam	ab32420
anti-Phospho-Chk2 (Thr68)	polyclonal	Cell Signaling Technology	2661
anti-p53	polyclonal	Cell Signaling Technology	9282
anti-Phospho-p53 (Ser15)	polyclonal	Cell Signaling Technology	9284
anti-STING	D2P2F	Cell Signaling Technology	13647

Secondary antibodies conjugated to horseradish peroxidase (HRP) were procured from Jackson ImmunoResearch.

Supplemental Table 6. Antibodies used for flow cytometric analysis.

Antibody Name	Clone Number	Company	Catalog Number
APC anti-human HLA-A,B,C Antibody	W6/32	BioLegend	311410
Brilliant Violet 421™ anti-mouse CD45 Antibody	30-F11	BioLegend	103133
APC-Cy7 Rat Anti-Mouse CD45	30-F11	BD Pharmingen	557659
BV605 Rat Anti-Mouse CD4	RM4-5	BD Pharmingen	563151
PerCP-Cy5.5 Rat Anti-Mouse CD8a	53-6.7	BD Pharmingen	551162
PE anti-mouse/human CD44 Antibody	IM7	BioLegend	103023
APC/Cyanine7 anti-mouse CD69 Antibody	H1.2F3	BioLegend	104525
APC/Cyanine7 anti-mouse CD45.1 Antibody	A20	BioLegend	110716

APC anti-mouse H-2Kb Antibody	AF6-88.5	BioLegend	116517
PE anti-mouse H-2Kb bound to SIINFEKL Antibody	25-D1.16	BioLegend	141604
FITC Hamster Anti-Mouse CD3e	145-2C11	BD Pharmingen	553061
PE Rat anti-Mouse Foxp3	MF23	BD Pharmingen	560408
Pacific Blue™ anti-human/mouse Granzyme B Antibody	GB11	BioLegend	515408
Brilliant Violet 421™ anti-human CD45 Antibody	2D1	BioLegend	368521
APC BrdU Flow Kit	-	BD Biosciences	559619

Supplemental Table 7. Clinical information of patients.

Patient	Gender	Age	Primary/Recurrence	Pathology	NMIBC/MIBC	Treatment
BCC1	M	80	R	H	MIBC	Atezolizumab
BCC3	F	83	P	H	MIBC	PC+Epirubicin
BCC15	M	48	P	L	NMIBC	TURBT+ Pirarubicin
BCC16	M	75	P	L	NMIBC	RC

BCC38	F	57	P	H	NMIBC	TURBT+ Epirubicin
BCC49	M	88	R	H	MIBC	RC+Paclitaxel+ PD-1
BCC101	M	65	R	L	NMIBC	TURBT
BCC102	M	63	P	L	NMIBC	TURBT+BCG

Gender: M= Male, F= Female; Primary/Recurrence: P= Primary, R= Recurrence; Pathology: H=high grade, L=low grade; MIBC = Muscle-invasive bladder cancer, NMIBC = Non-muscle-invasive bladder cancer; Treatment: TURBT=transurethral resection of bladder tumor, PC=Partial cystectomy, RC=Radical cystectomy.

4. References

1. Zeng D, Ye Z, Shen R, Yu G, Wu J, Xiong Y, et al. IOBR: Multi-Omics Immuno-Oncology Biological Research to Decode Tumor Microenvironment and Signatures. *Frontiers in immunology.* 2021;12:687975.
2. Hao Y, Hao S, Andersen-Nissen E, Mauck WM, 3rd, Zheng S, Butler A, et al. Integrated analysis of multimodal single-cell data. *Cell.* 2021;184(13):3573-87.e29.
3. Hafemeister C, and Satija R. Normalization and variance stabilization of single-cell RNA-seq data using regularized negative binomial regression. *Genome biology.* 2019;20(1):296.
4. Gulati GS, Sikandar SS, Wesche DJ, Manjunath A, Bharadwaj A, Berger MJ, et al. Single-cell transcriptional diversity is a hallmark of developmental potential. *Science (New York, NY).* 2020;367(6476):405-11.
5. Wu T, Hu E, Xu S, Chen M, Guo P, Dai Z, et al. clusterProfiler 4.0: A universal enrichment tool for interpreting omics data. *Innovation (Cambridge (Mass)).* 2021;2(3):100141.
6. Jin S, Guerrero-Juarez CF, Zhang L, Chang I, Ramos R, Kuan CH, et al. Inference and analysis of cell-cell communication using CellChat. *Nature communications.* 2021;12(1):1088.
7. Trapnell C, Cacchiarelli D, Grimsby J, Pokharel P, Li S, Morse M, et al. The dynamics and regulators of cell fate decisions are revealed by pseudotemporal ordering of single cells. *Nature biotechnology.* 2014;32(4):381-6.
8. Morabito S, Reese F, Rahimzadeh N, Miyoshi E, and Swarup V. hdWGCNA identifies co-expression networks in high-dimensional transcriptomics data. *Cell reports methods.* 2023;3(6):100498.
9. Morabito S, Miyoshi E, Michael N, Shahin S, Martini AC, Head E, et al. Single-nucleus chromatin accessibility and transcriptomic characterization of Alzheimer's disease. *Nature genetics.* 2021;53(8):1143-55.
10. Chen EY, Tan CM, Kou Y, Duan Q, Wang Z, Meirelles GV, et al. Enrichr: interactive and collaborative HTML5 gene list enrichment analysis tool. *BMC bioinformatics.* 2013;14:128.
11. Kuleshov MV, Jones MR, Rouillard AD, Fernandez NF, Duan Q, Wang Z, et al. Enrichr: a comprehensive gene set enrichment analysis web server 2016 update. *Nucleic acids research.* 2016;44(W1):W90-7.
12. Xie Z, Bailey A, Kuleshov MV, Clarke DJB, Evangelista JE, Jenkins SL, et al. Gene Set Knowledge Discovery with Enrichr. *Current protocols.* 2021;1(3):e90.
13. Schubert M, Klinger B, Klünemann M, Sieber A, Uhlitz F, Sauer S, et al. Perturbation-response genes reveal signaling footprints in cancer gene expression. *Nature communications.* 2018;9(1):20.
14. Holland CH, Tanevski J, Perales-Patón J, Gleixner J, Kumar MP, Mereu E, et al. Robustness and applicability of transcription factor and pathway analysis tools on single-cell RNA-seq data. *Genome biology.* 2020;21(1):36.
