# Supplementary material for: Pharmacological induction of MHC-I expression in tumor cells revitalizes T cell antitumor immunity
Source: JCI Insight. 2024 Aug 6;9(17):e177788. doi: 10.1172/jci.insight.177788 (PMC11385079; doi:10.1172/jci.insight.177788)

Full unedited gel for Figure 1E

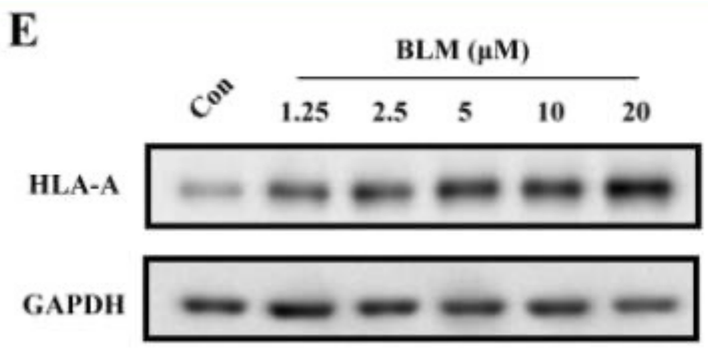

HLA-A

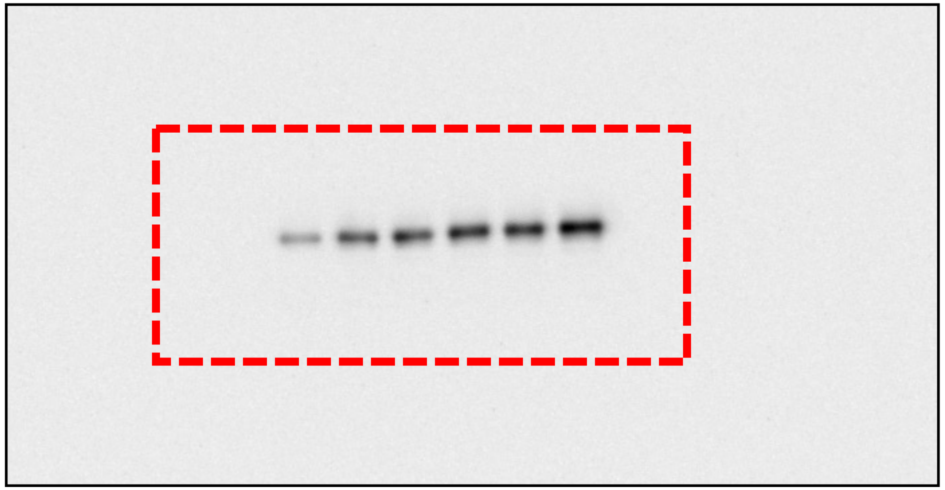

IB:HLA-A

GAPDH

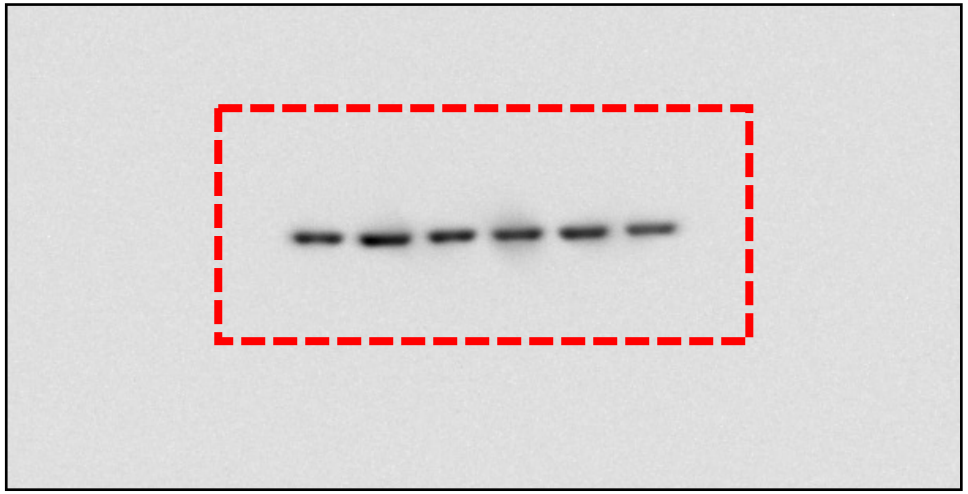

IB:GAPDH

Full unedited gel for Figure 1F

F

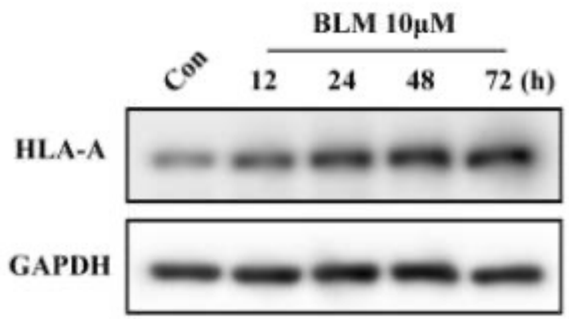

HLA-A

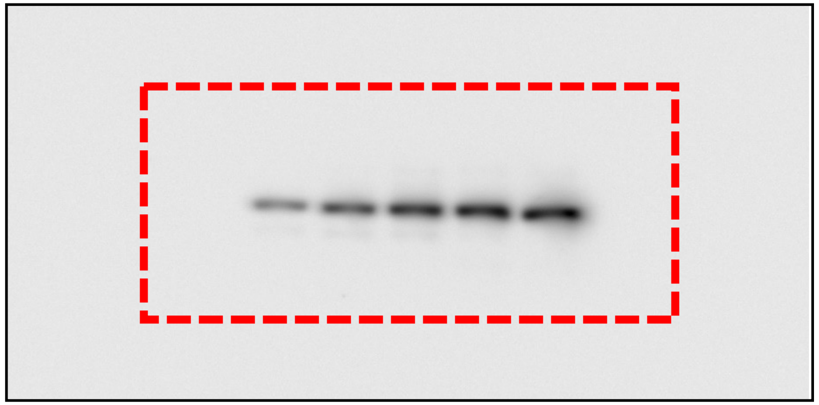

IB:HLA-A

GAPDH

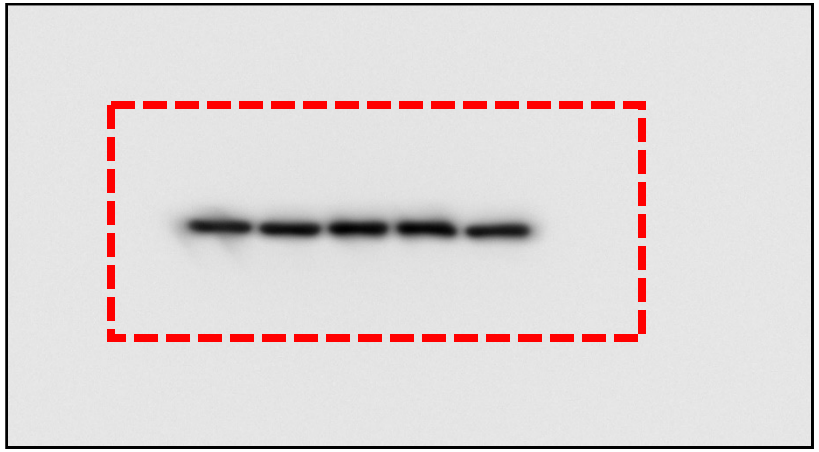

IB:GAPDH

Full unedited gel for Figure 2E

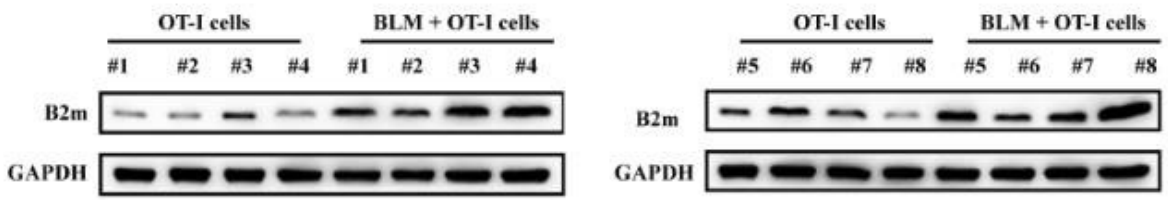

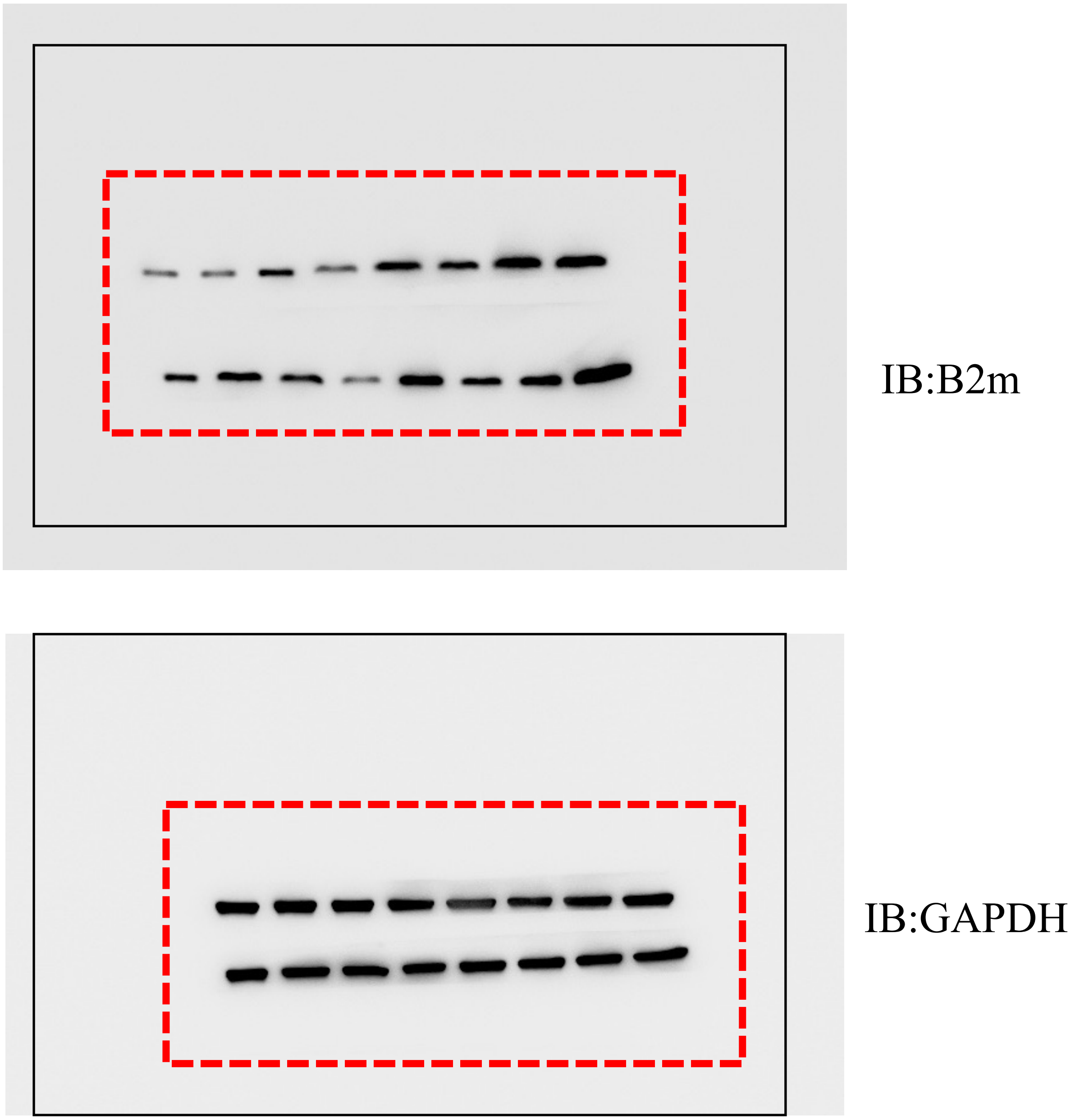

Full unedited gel for Figure 5B

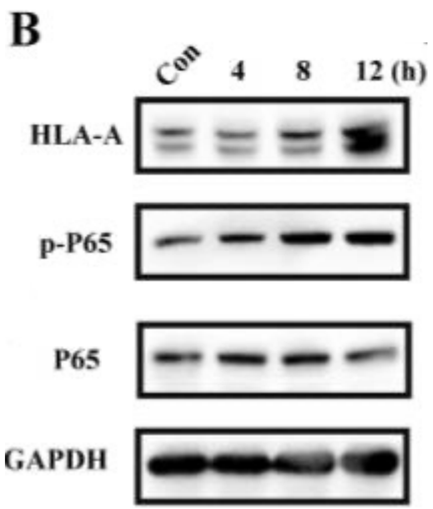

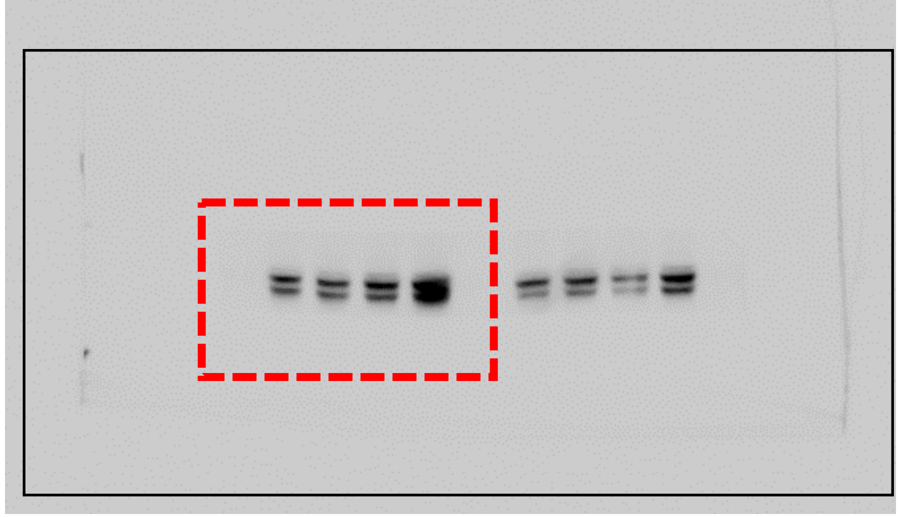

IB:HLA-A

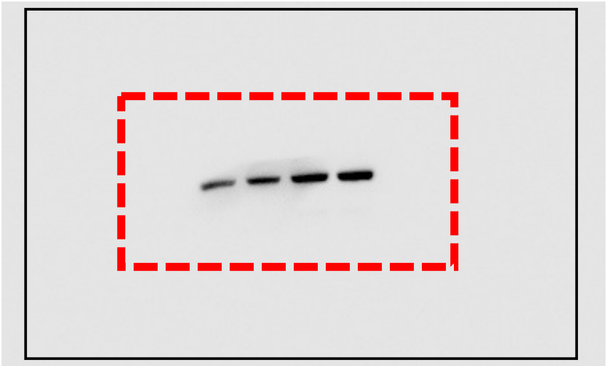

IB:p-P65

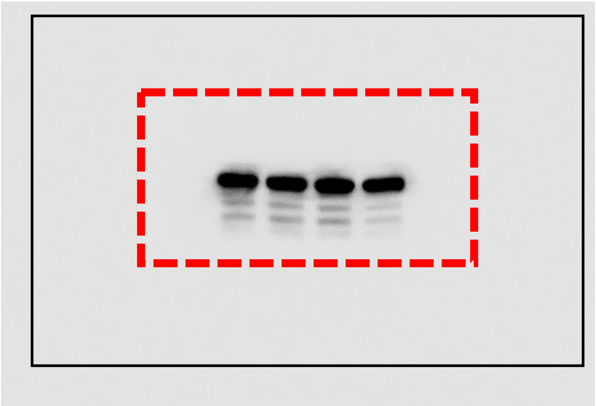

IB:P65

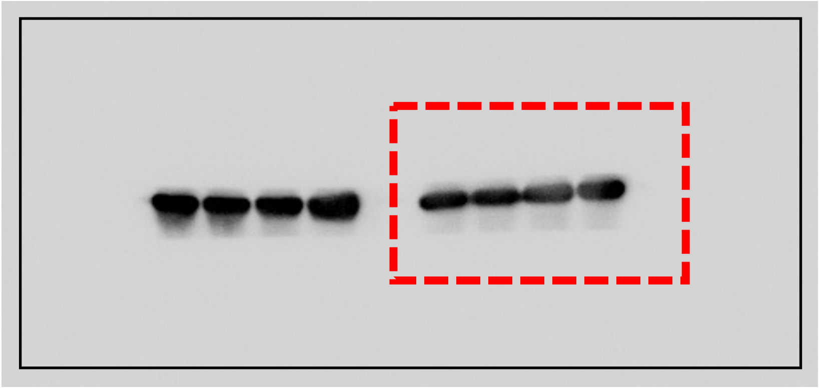

IB:GAPDH

Full unedited gel for Figure 5C

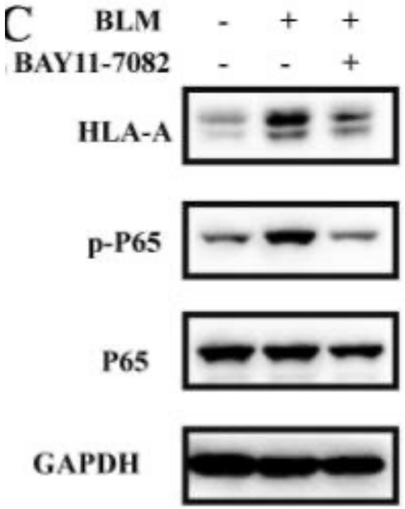

IB:P65

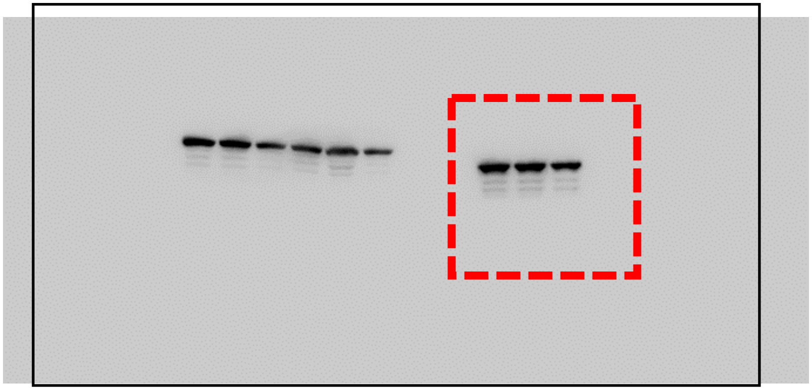

IB:GAPDH

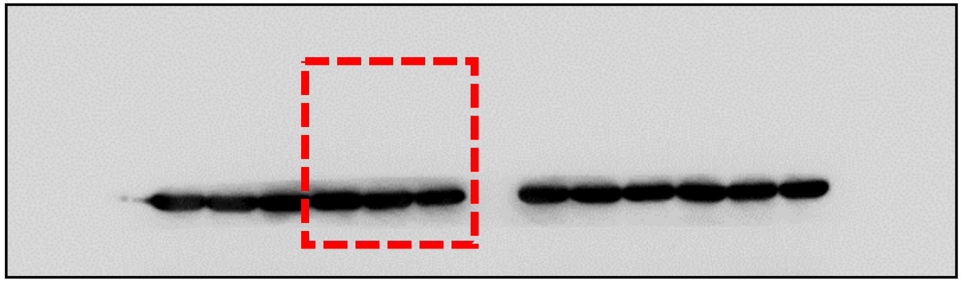

IB:p-P65

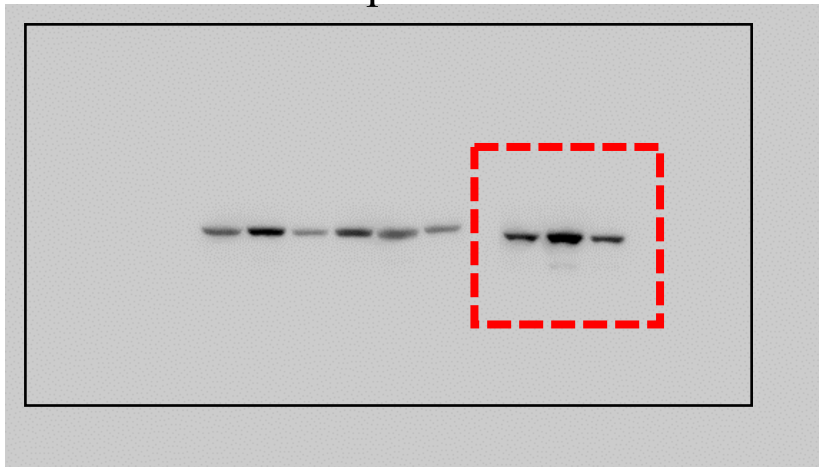

IB:HLA-A

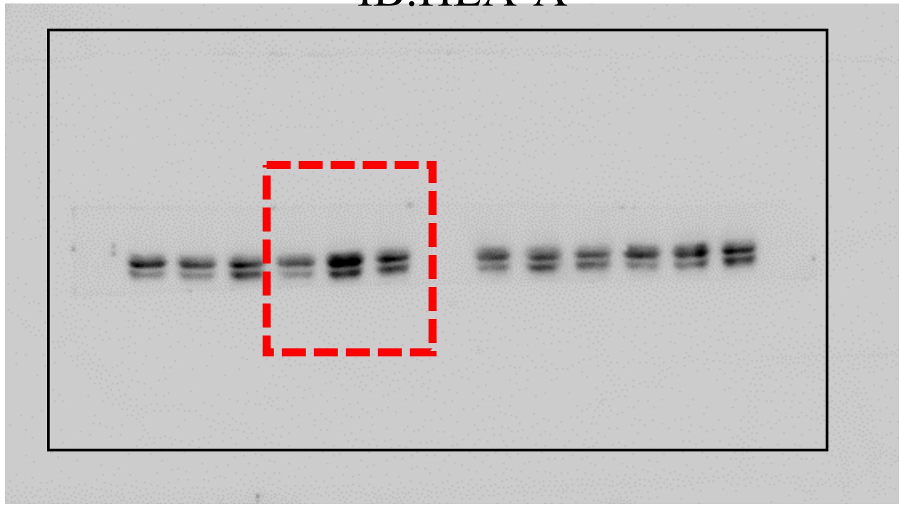

Full unedited gel for Figure 5D

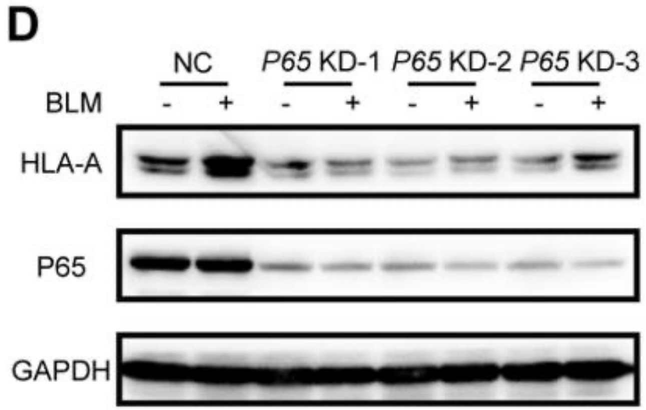

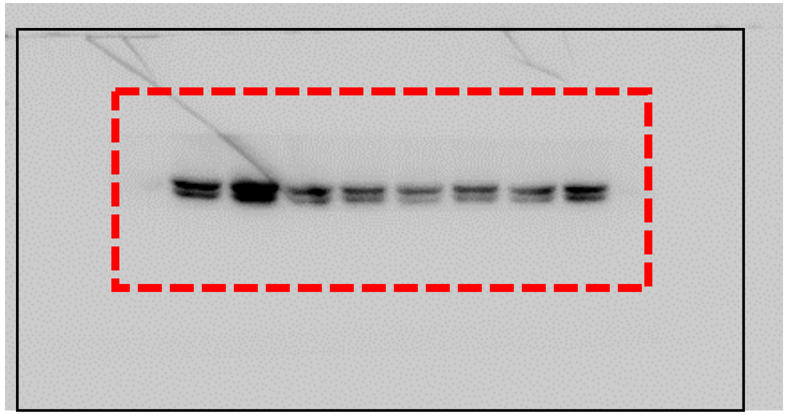

IB:HLA-A

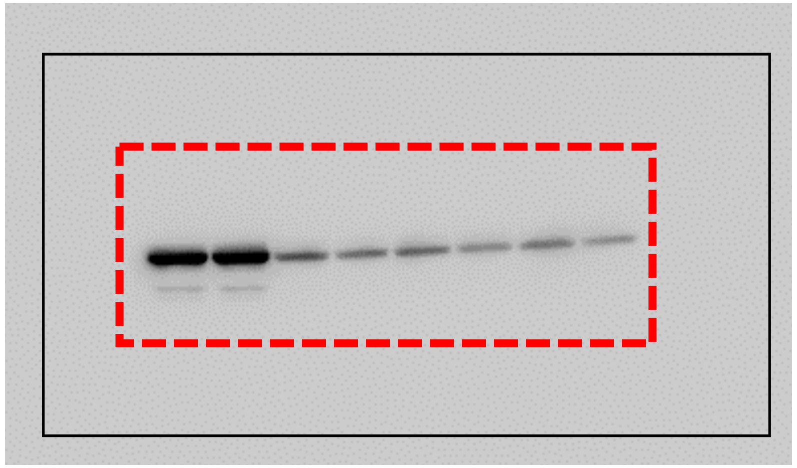

IB:P65

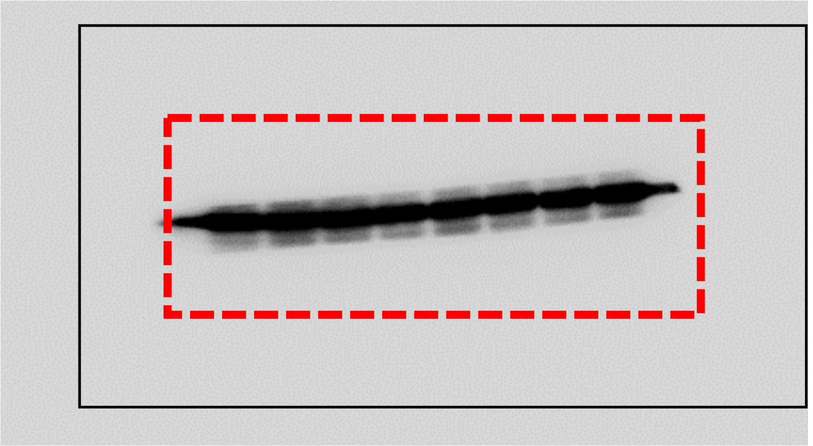

IB:GAPDH

Full unedited gel for Figure 5G

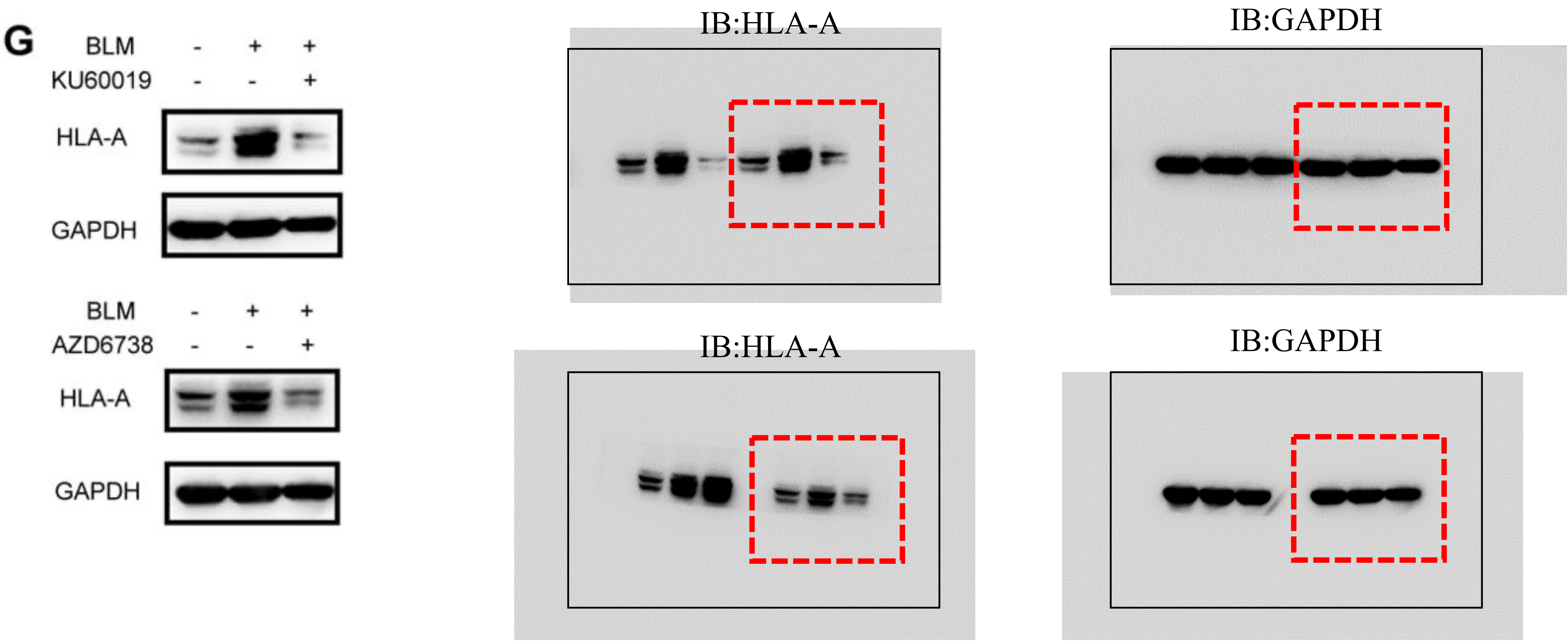

Full unedited gel for Figure 5I

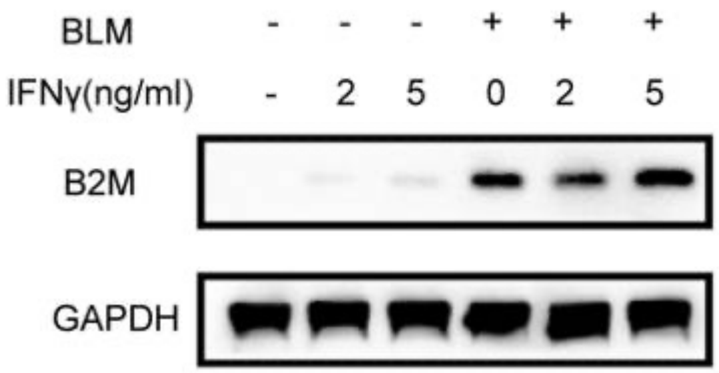

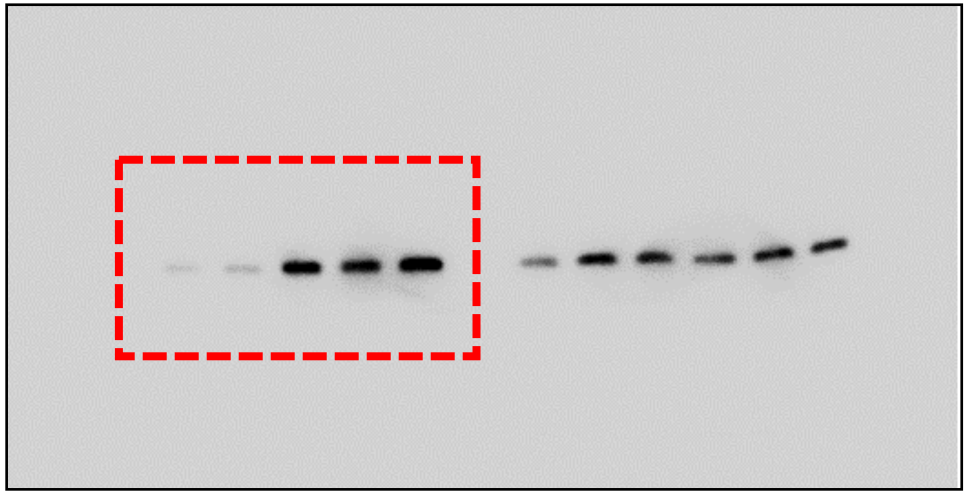

IB:B2M

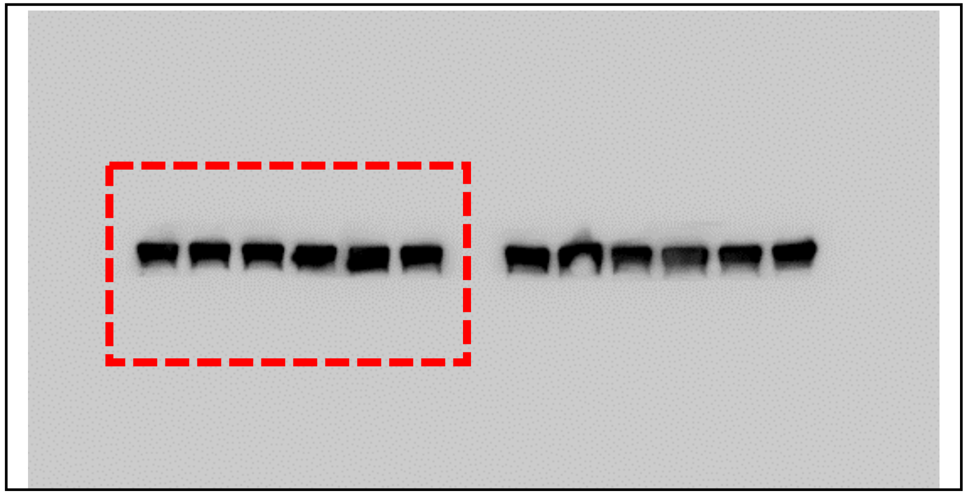

IB:GAPDH

Full unedited gel for Supplemental Figure 2A

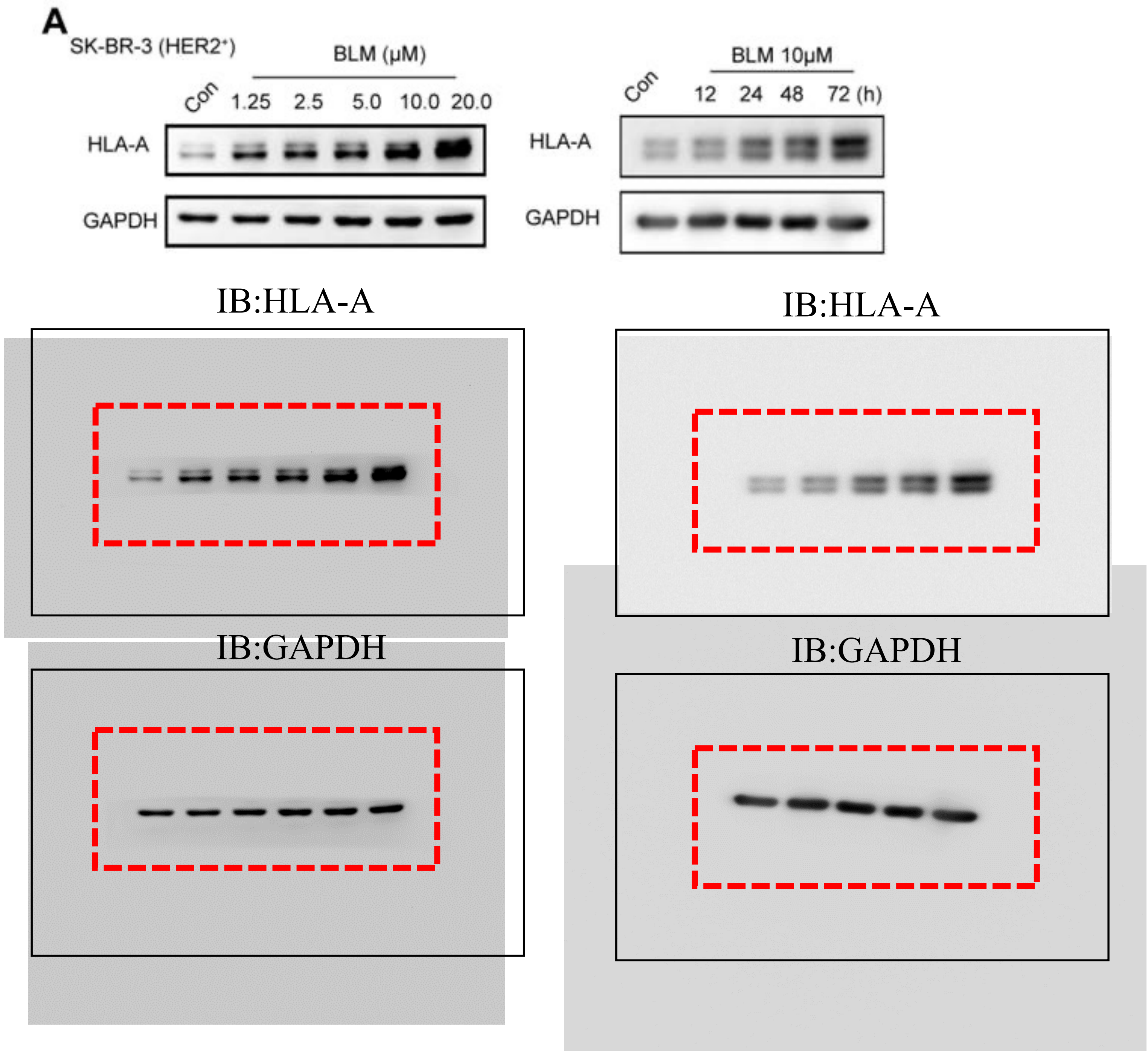

Full unedited gel for Supplemental Figure 2B

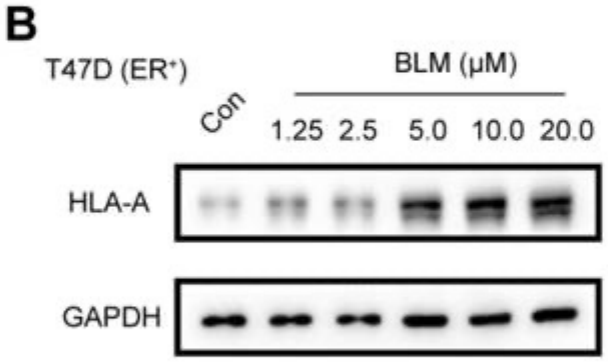

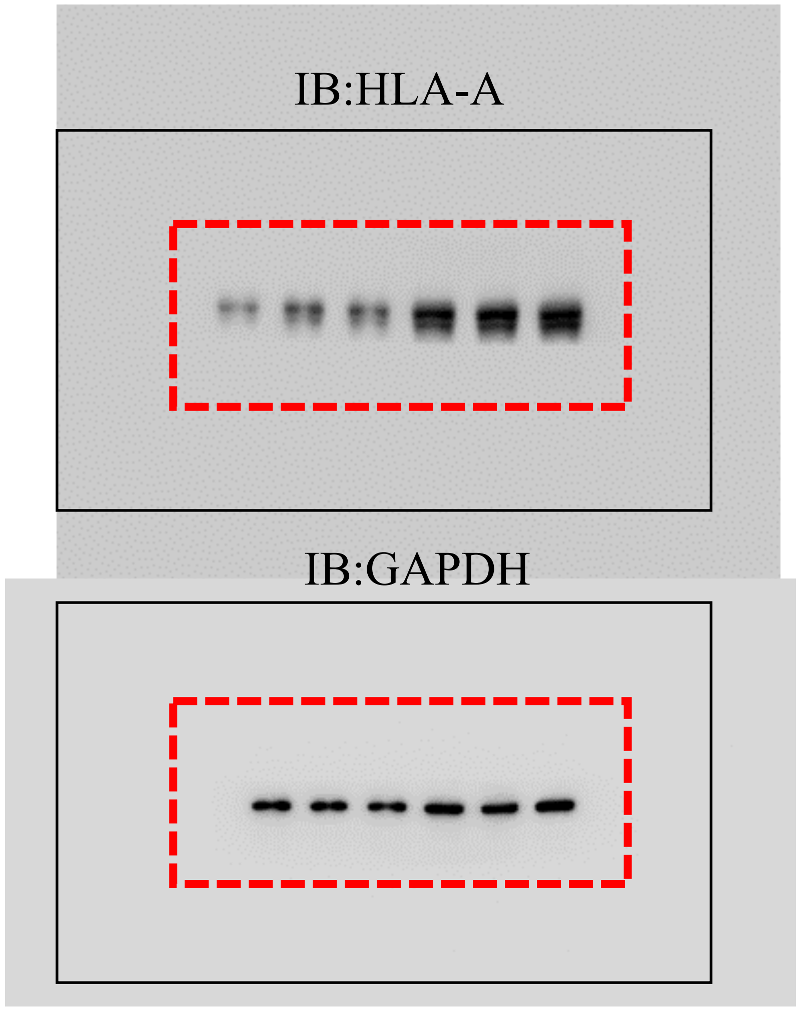

Supplemental Figure 2C

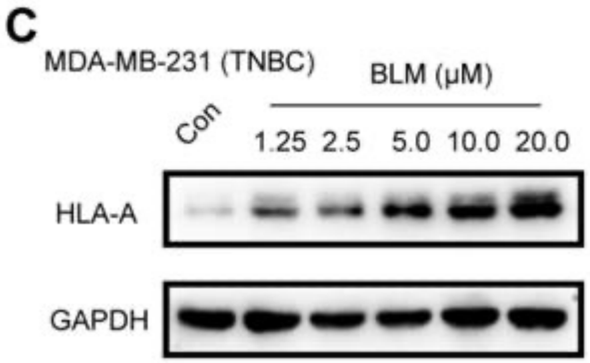

IB:HLA-A

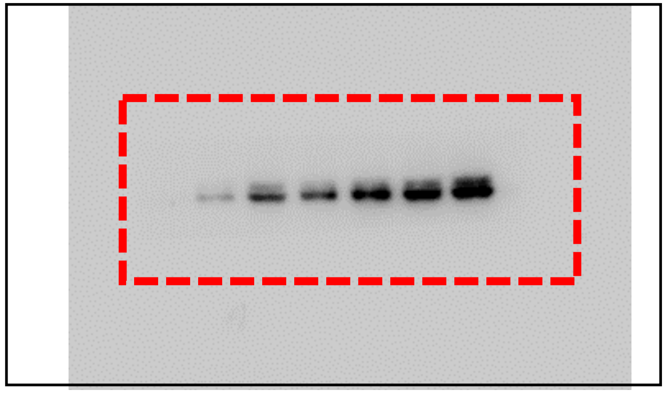

IB:GAPDH

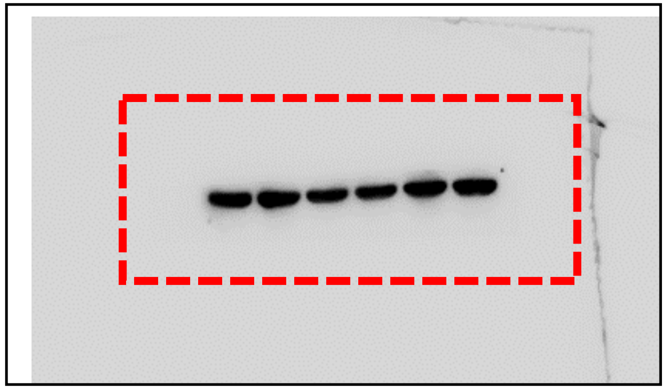

Full unedited gel for Supplemental Figure 2D

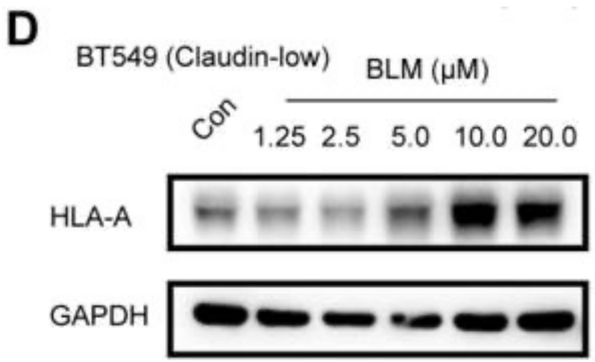

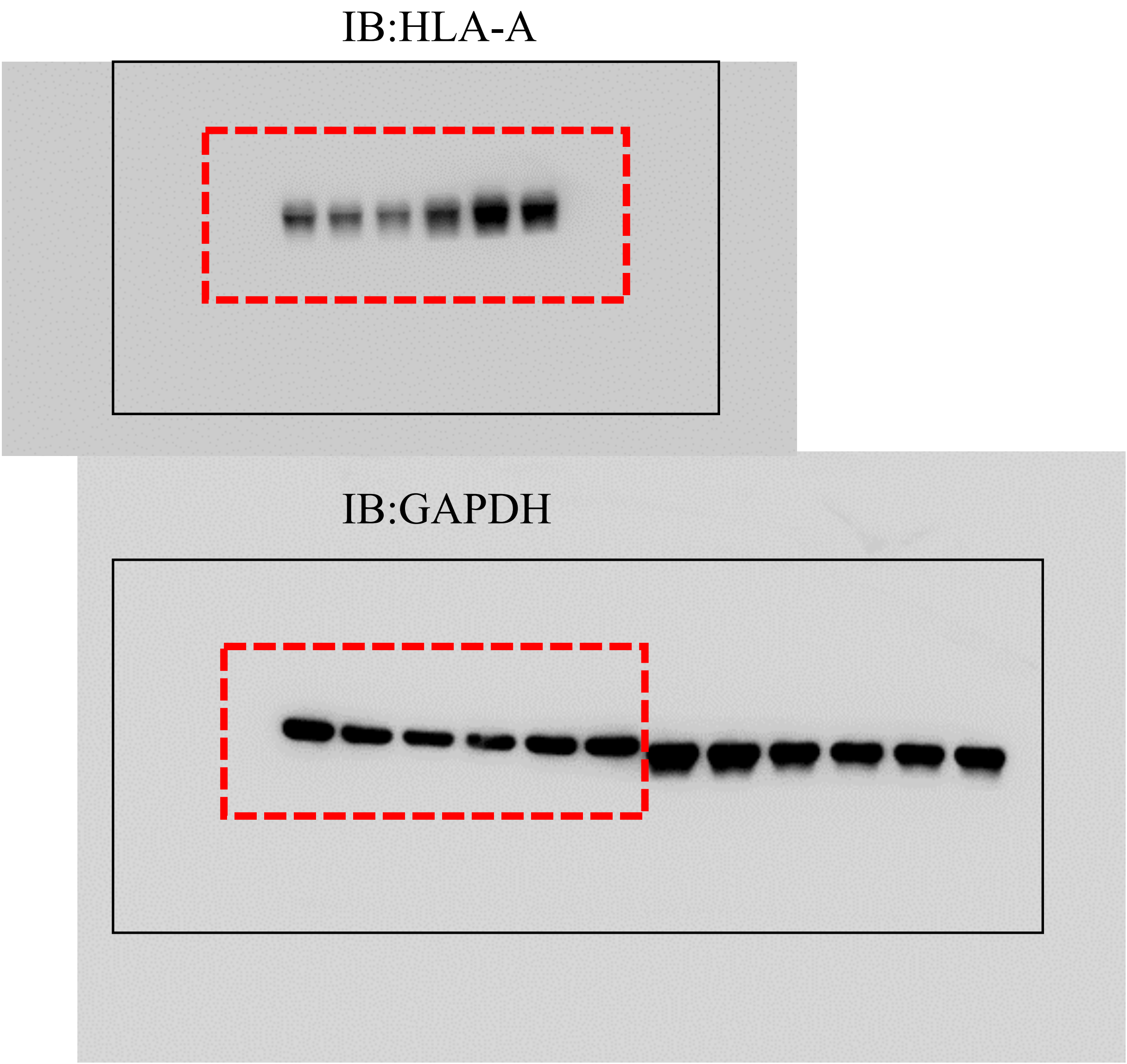

Full unedited gel for Supplemental Figure 2E

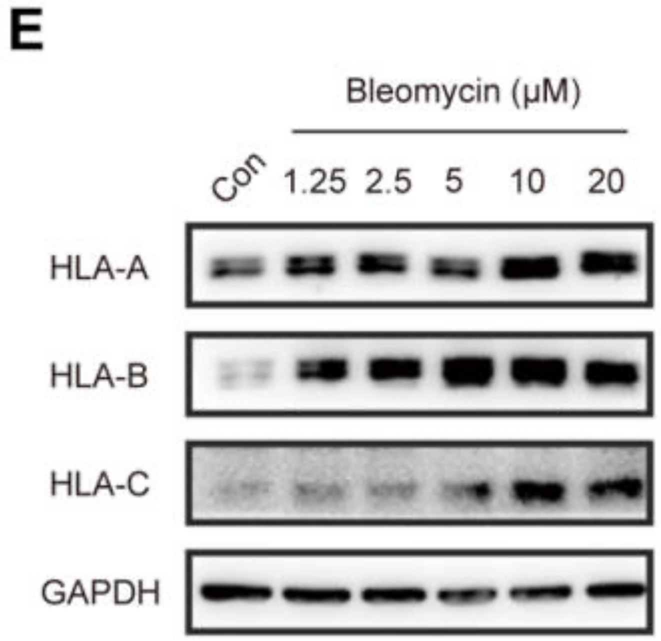

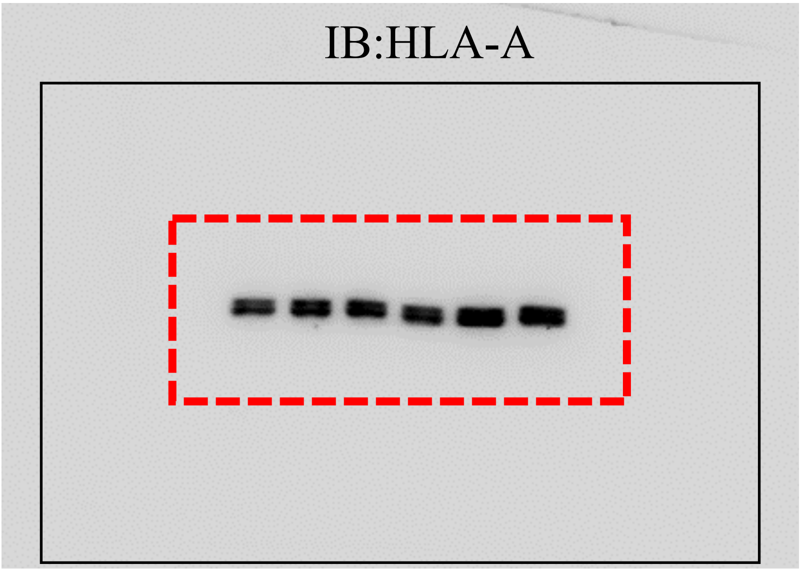

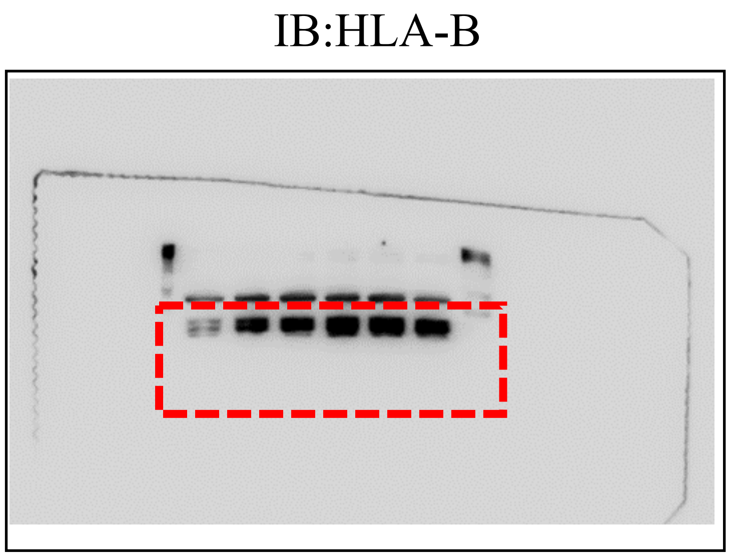

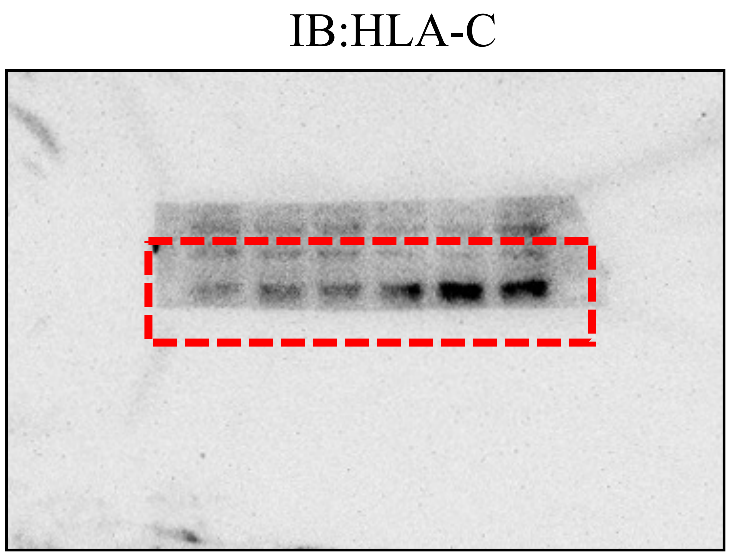

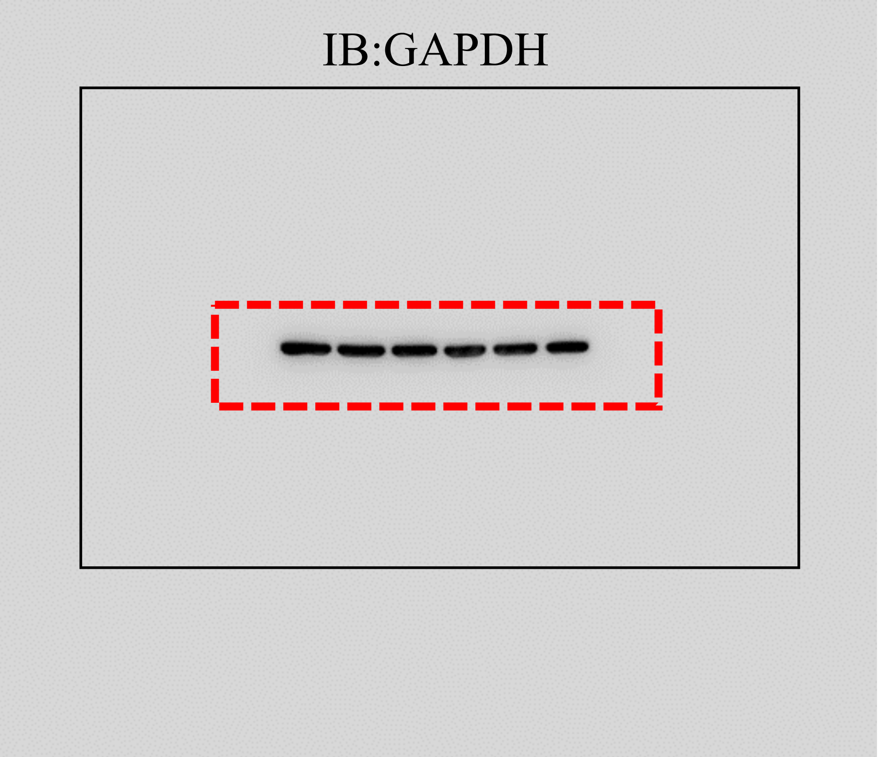

Full unedited gel for Supplemental Figure 2F

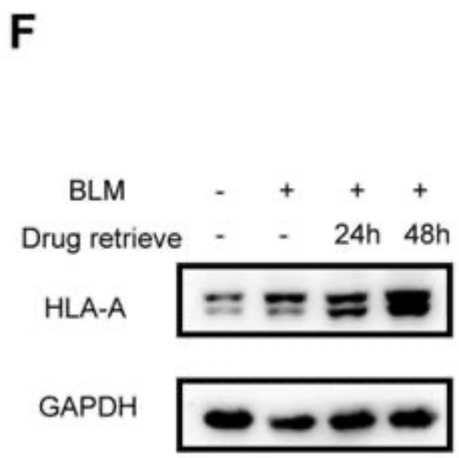

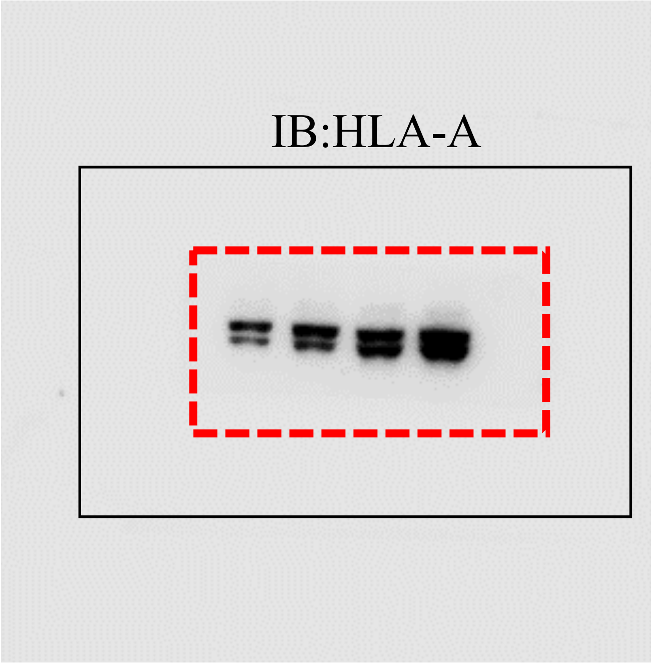

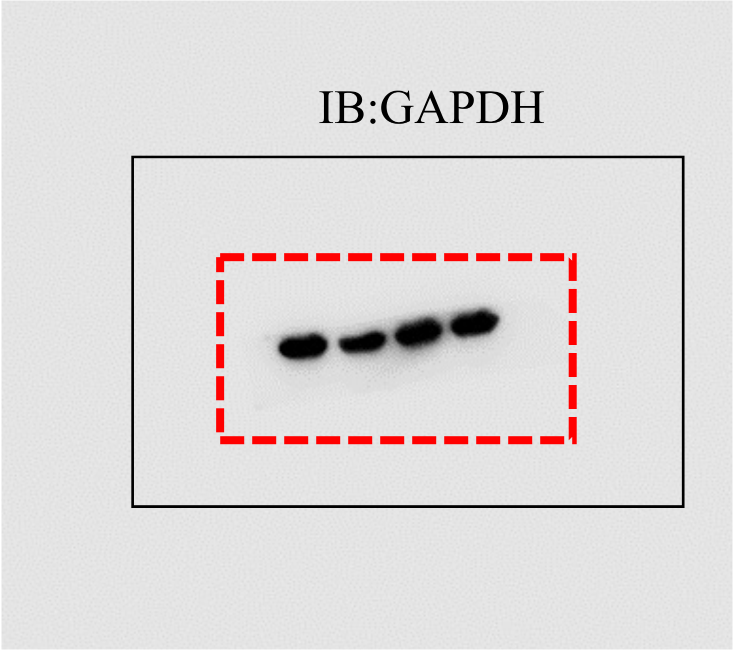

Full unedited gel for Supplemental Figure 3D

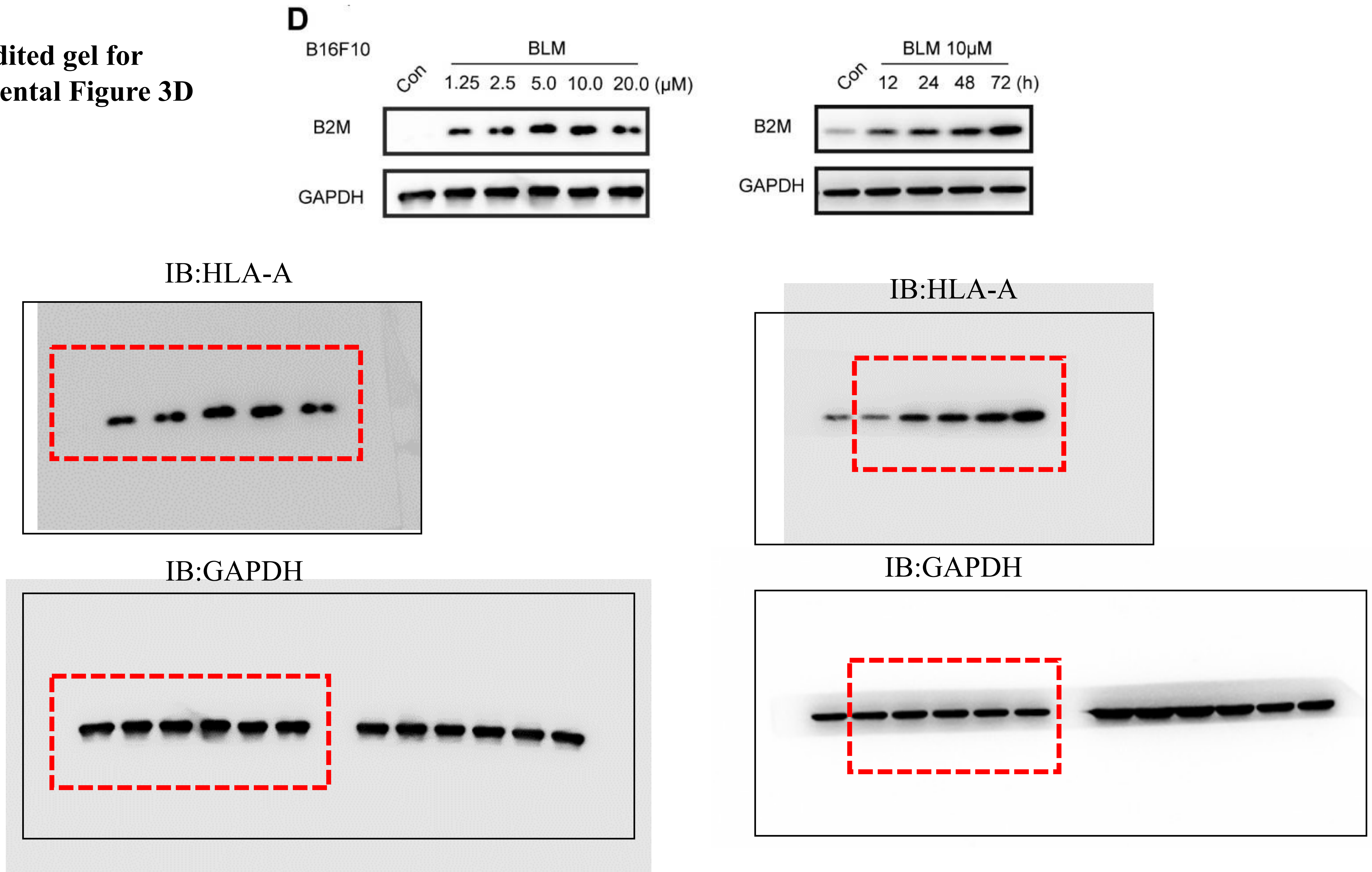

Full unedited gel for
Supplemental Figure 5B

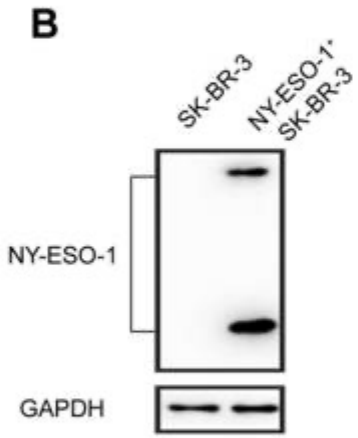

IB:NY-ESO-1

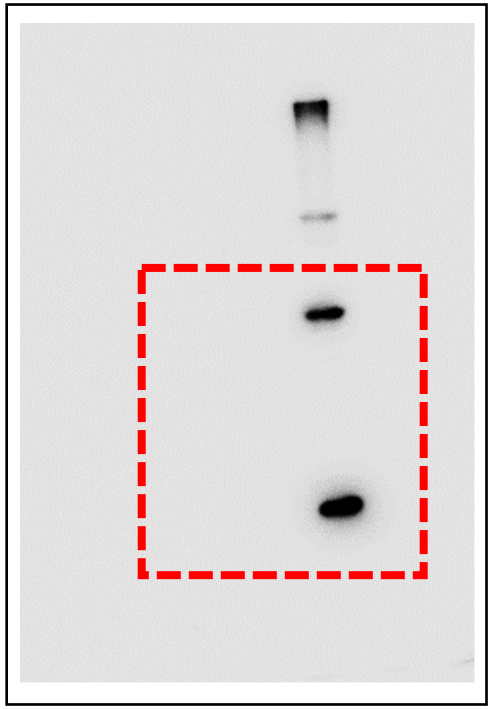

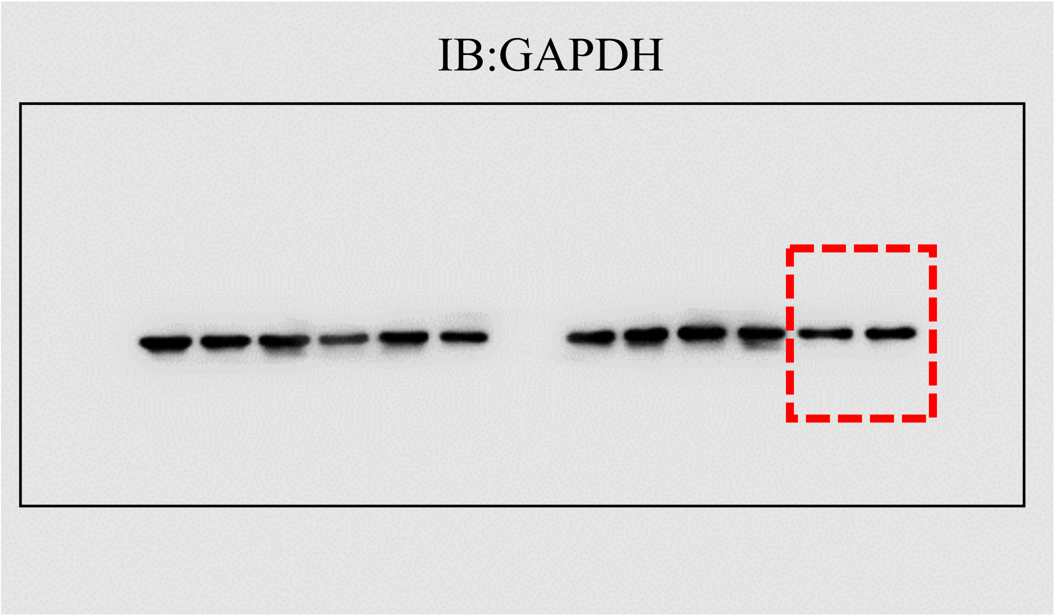

Full unedited gel for Supplemental Figure 8A

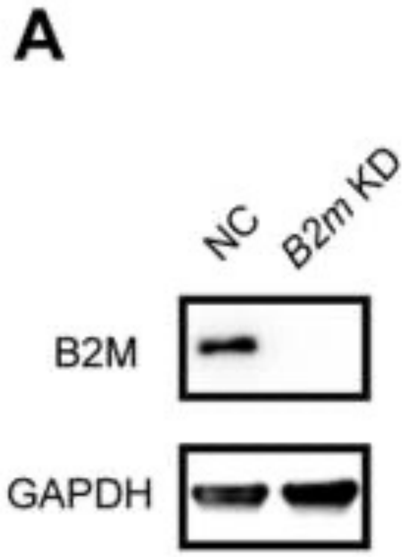

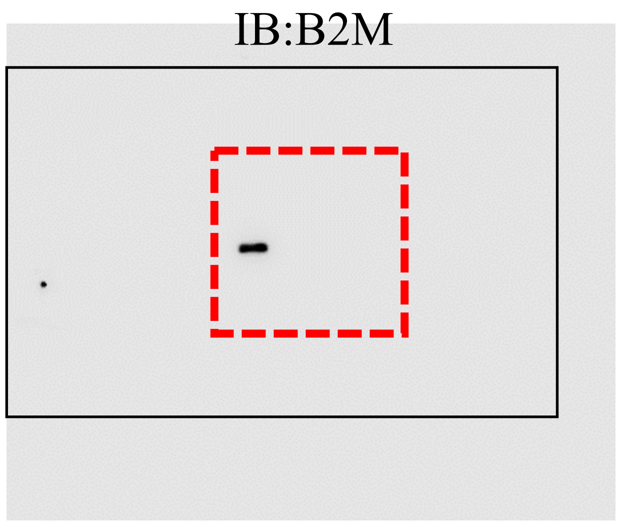

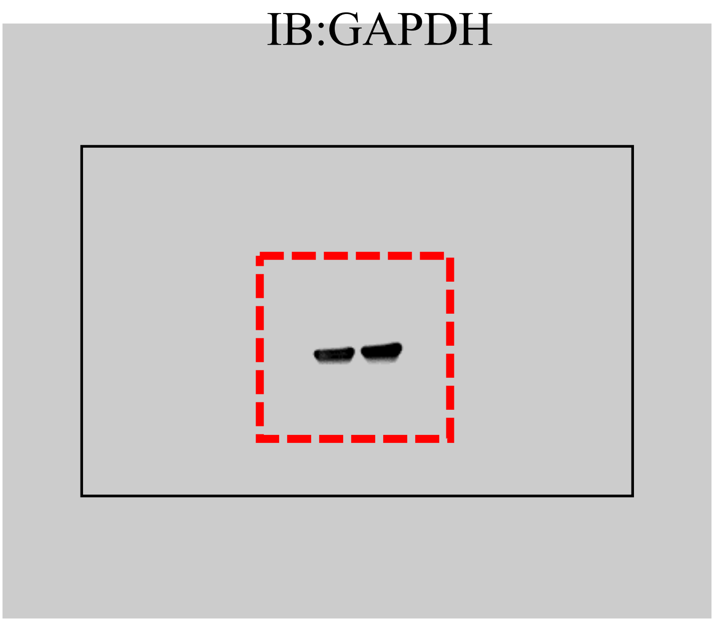

Full unedited gel for Supplemental Figure 8B

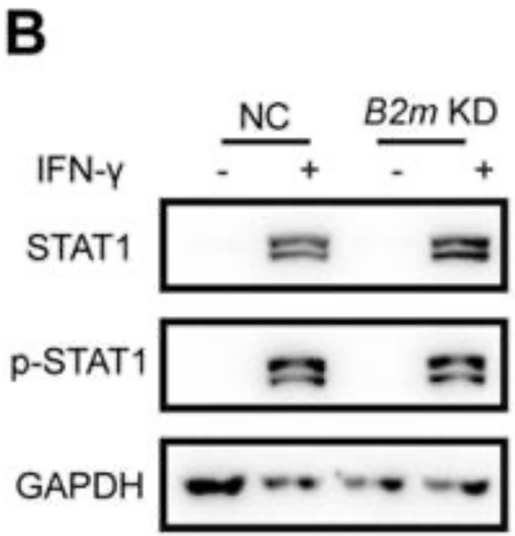

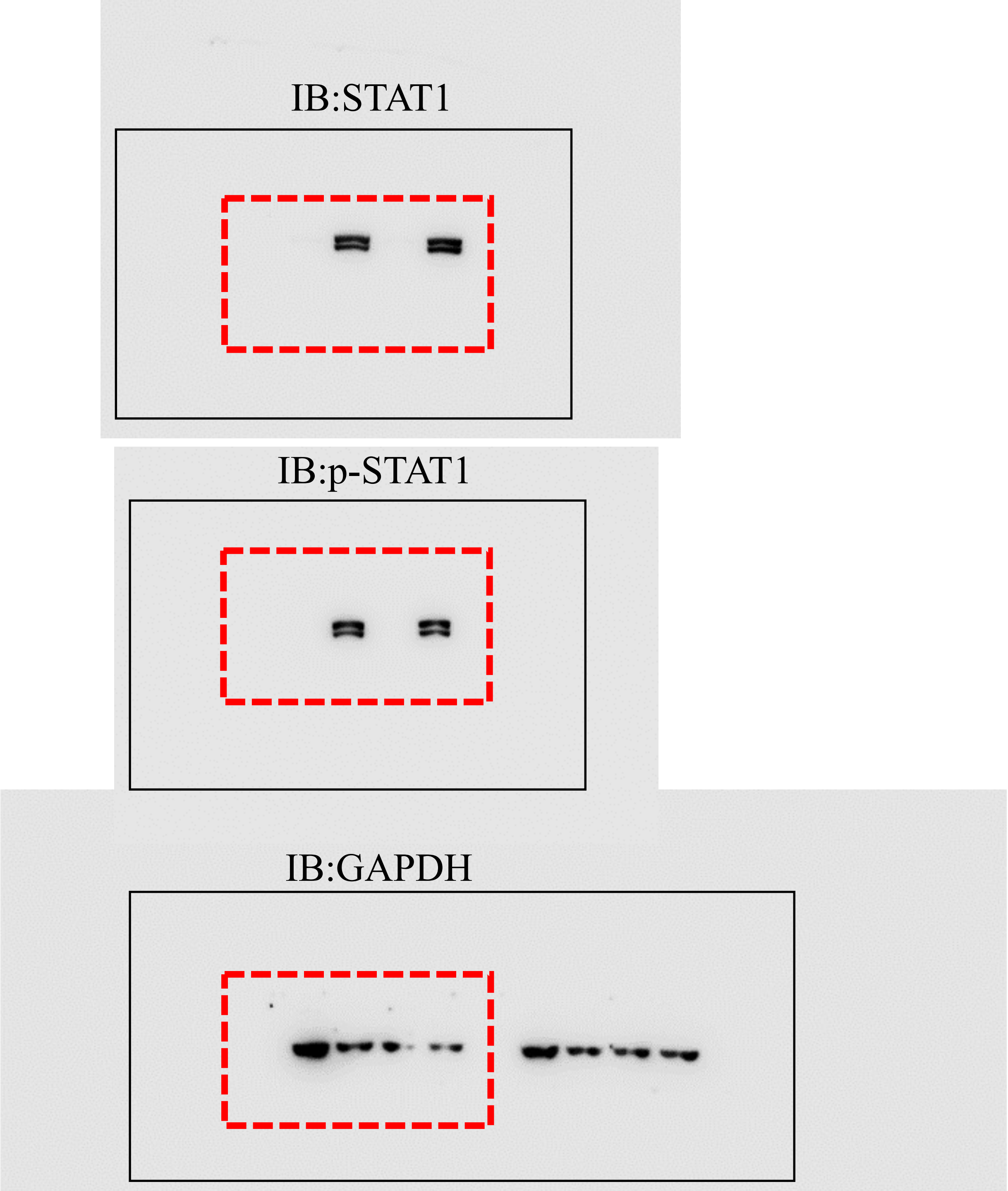

Full unedited gel for Supplemental Figure 11B

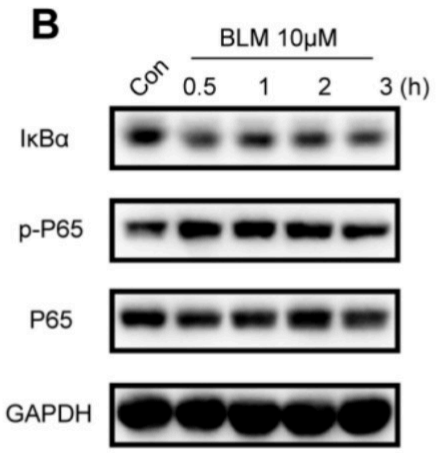

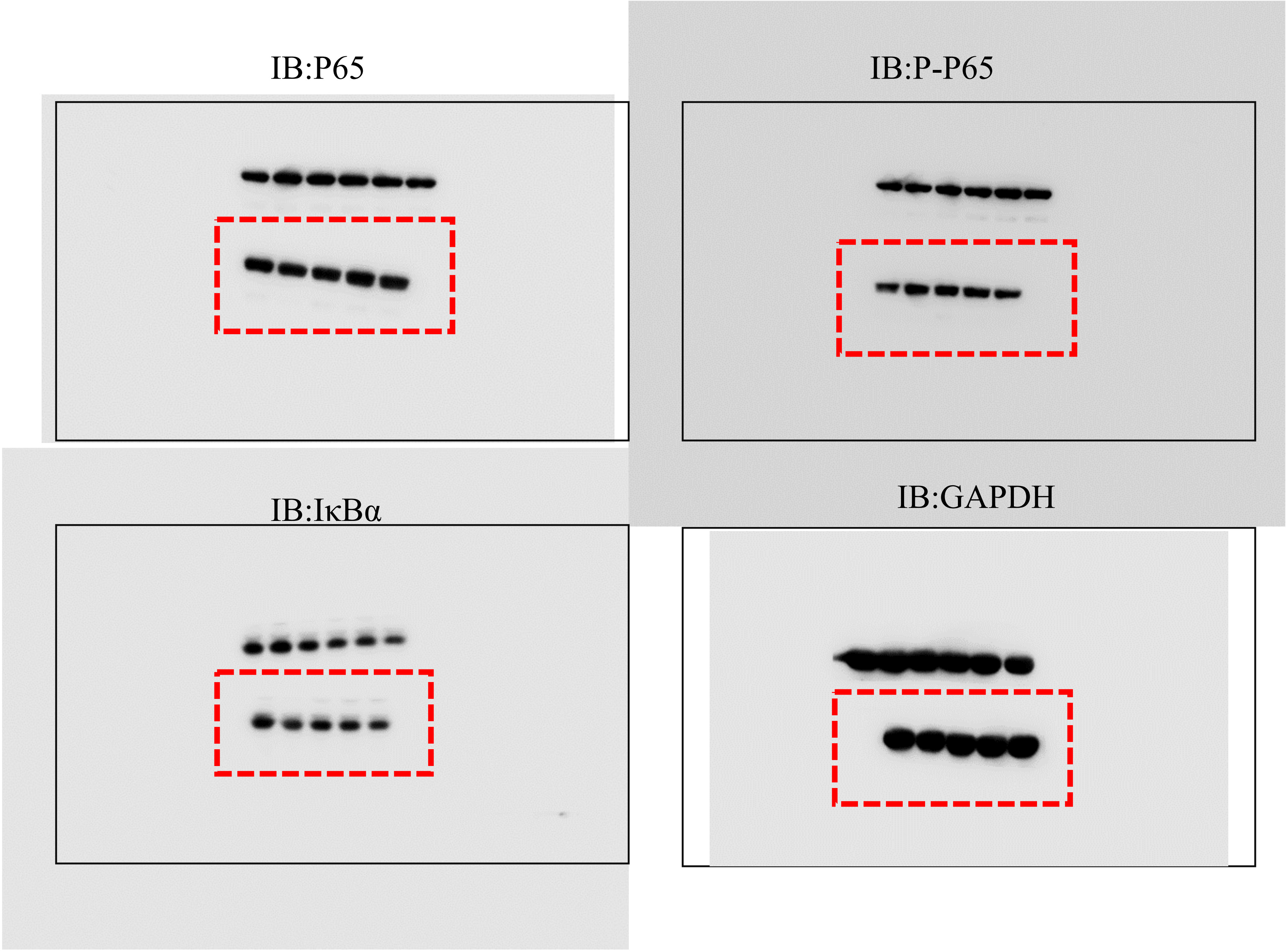

Full unedited gel for Supplemental Figure 11C

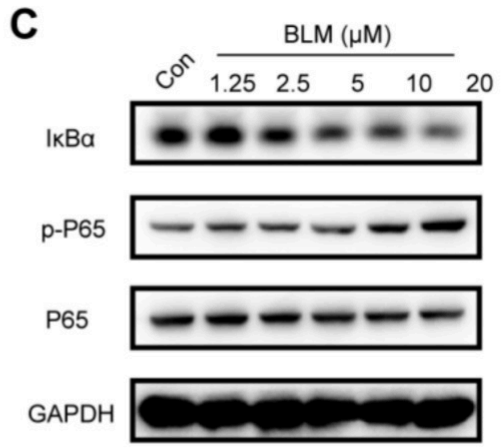

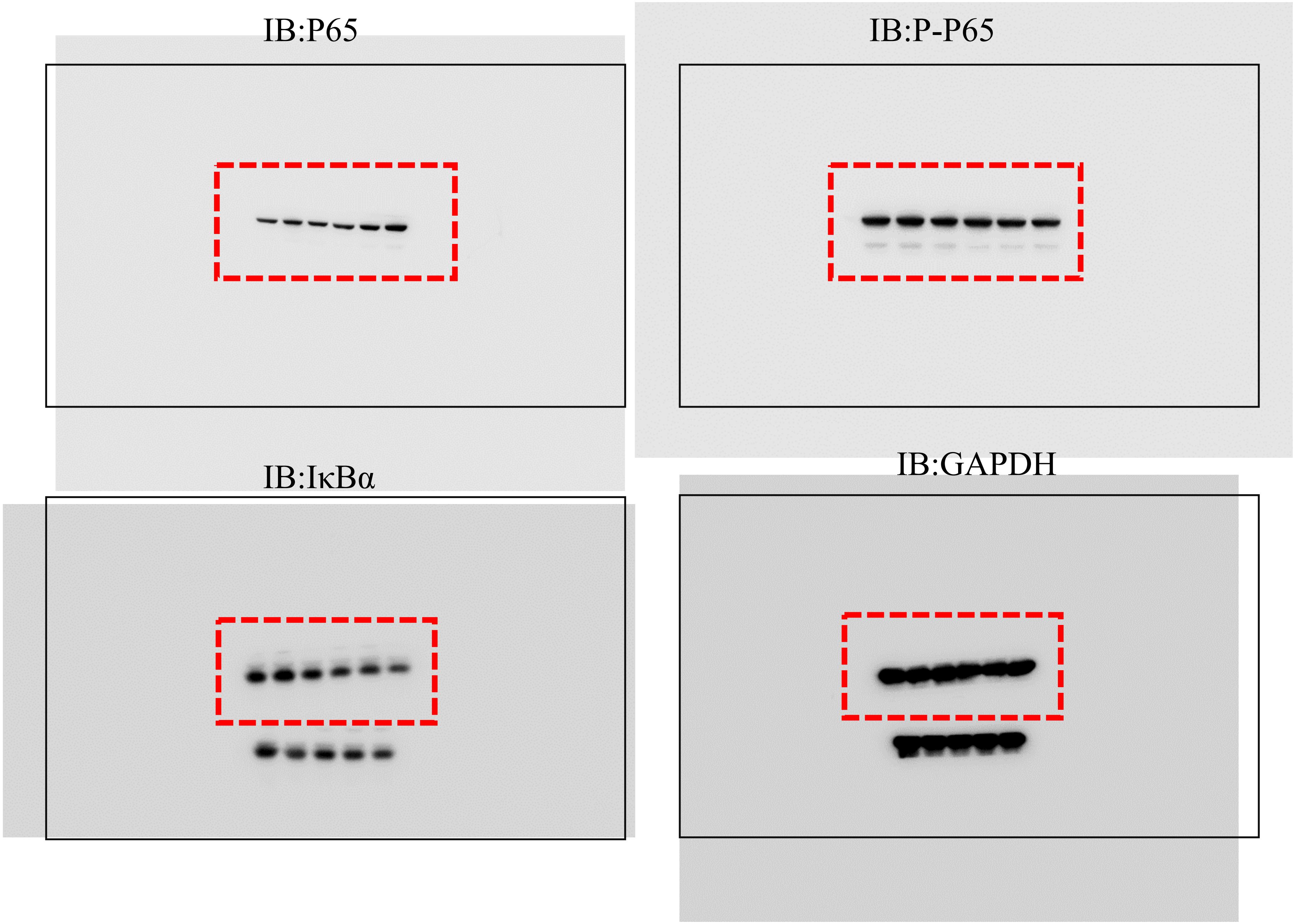

Full unedited gel for Supplemental Figure 11D

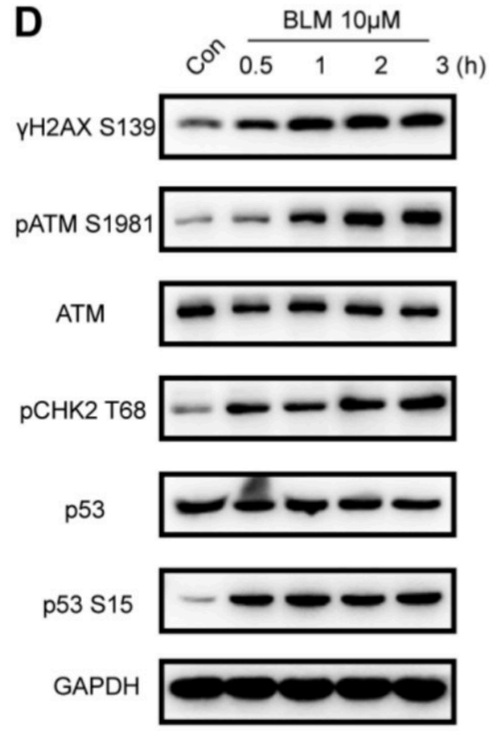

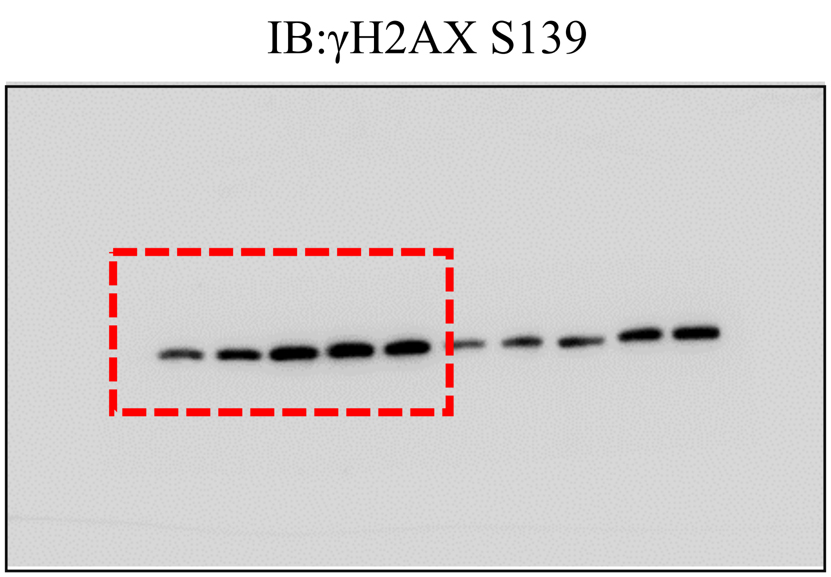

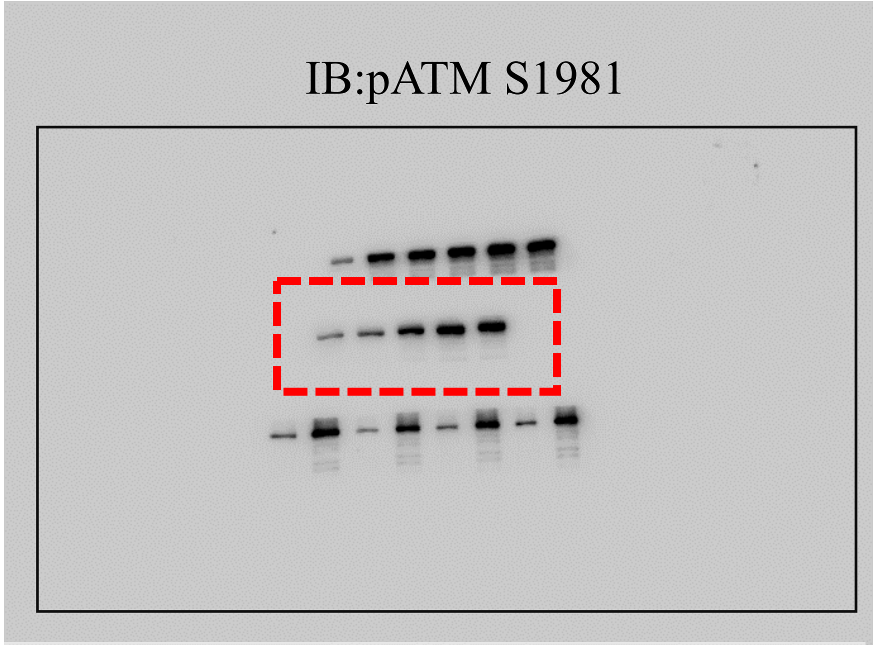

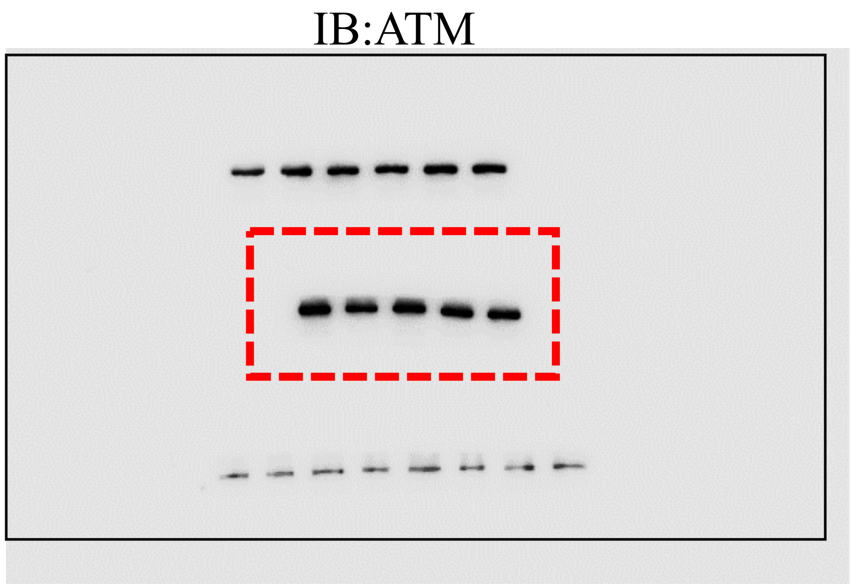

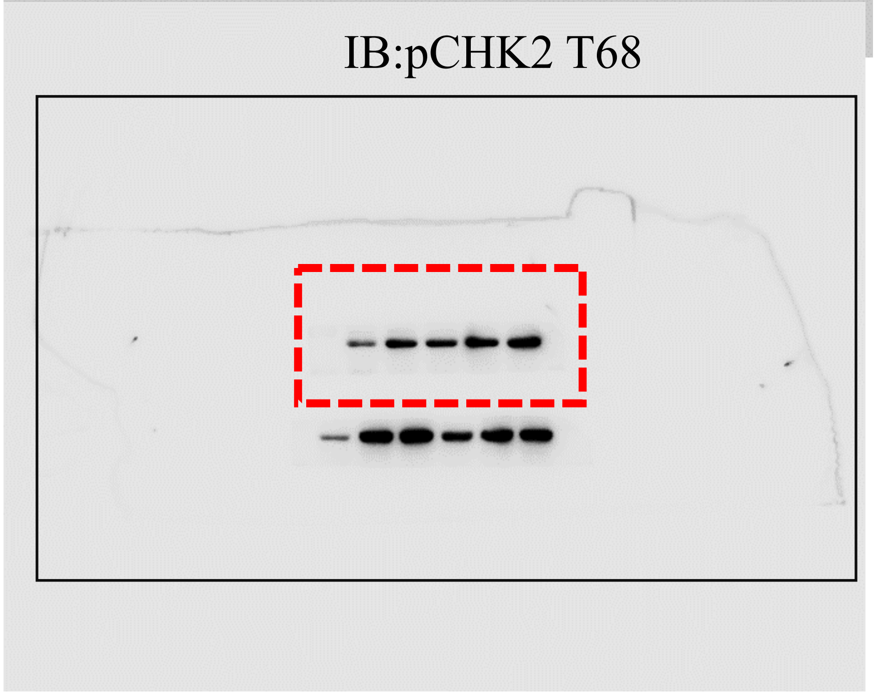

Full unedited gel for Supplemental Figure 11D

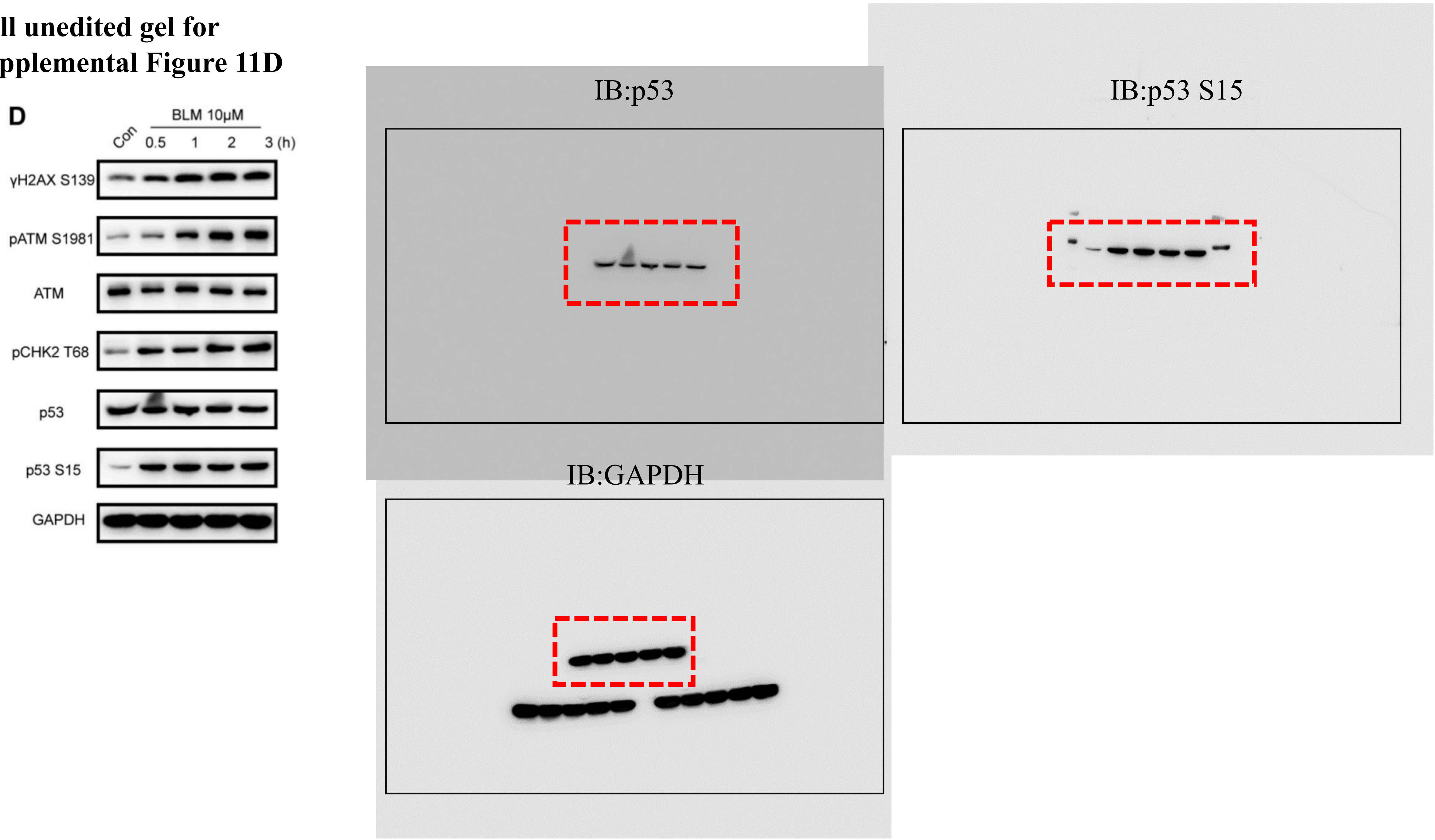

Full unedited gel for Supplemental Figure 11E

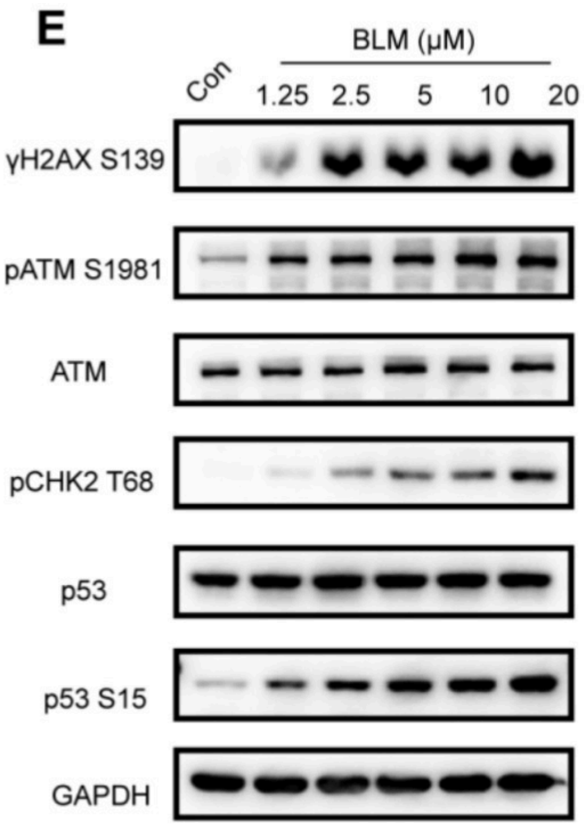

IB:γH2AX S139

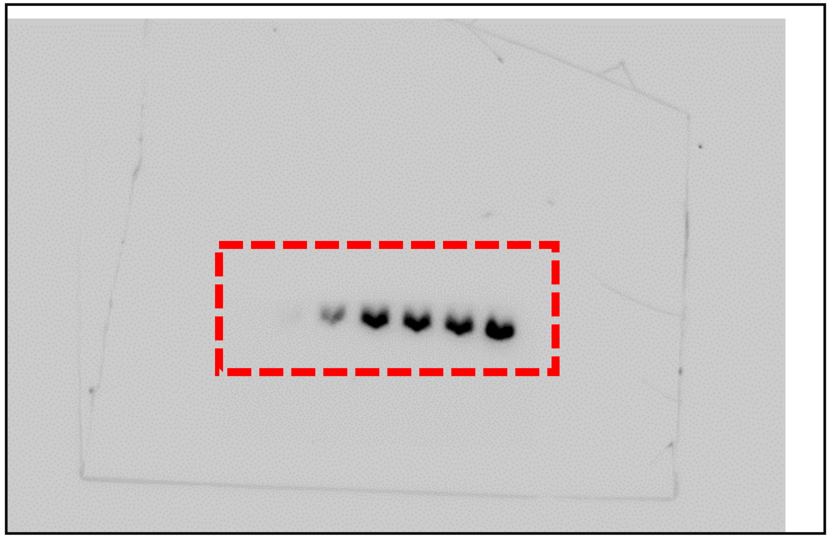

IB:pATM S1981

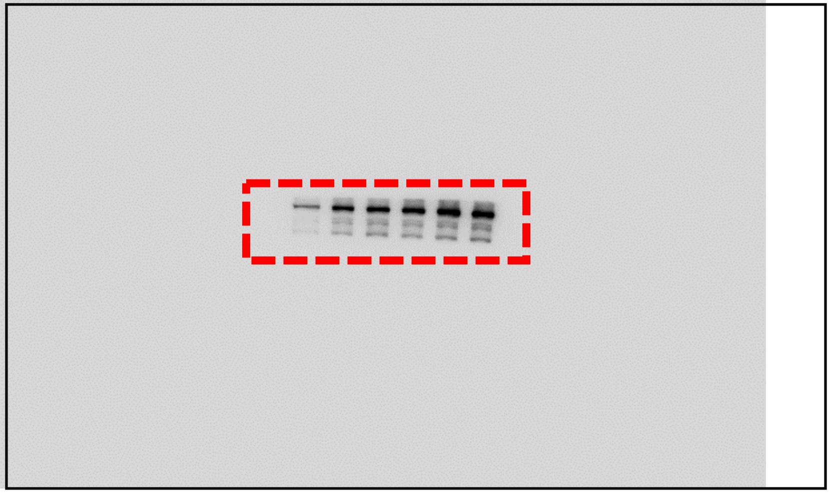

IB:ATM

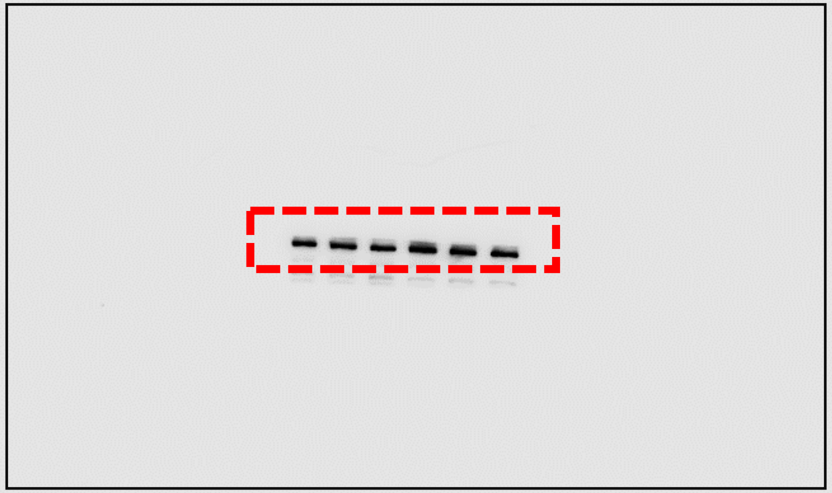

IB:pCHK2 T68

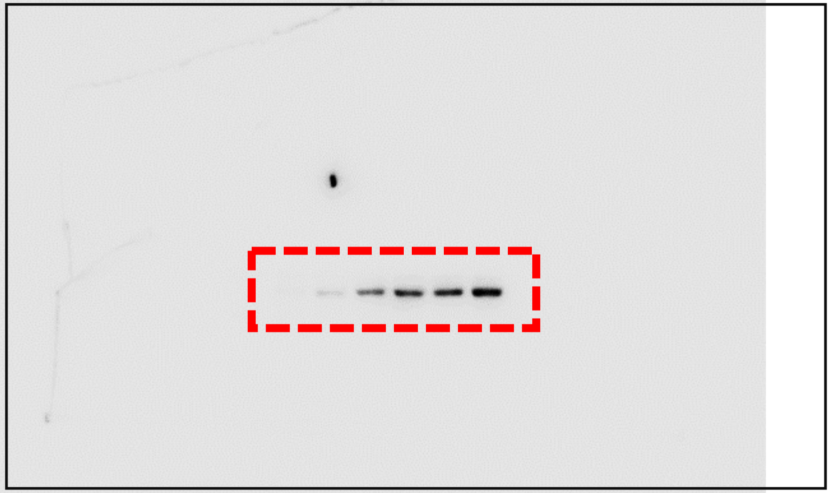

Full unedited gel for Supplemental Figure 11E

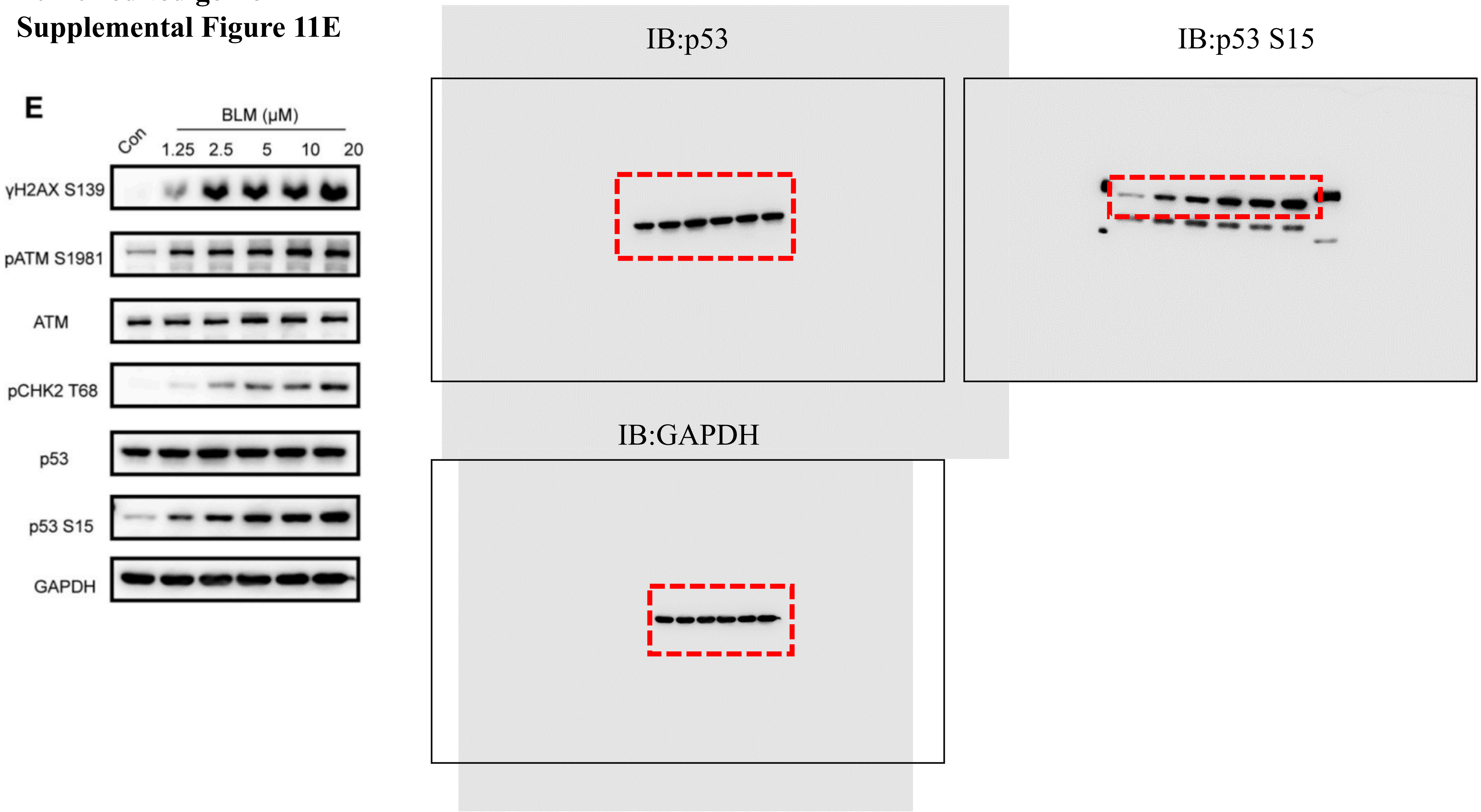

Full unedited gel for
Supplemental Figure 11F

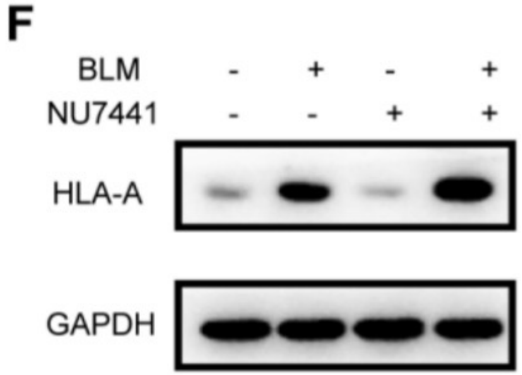

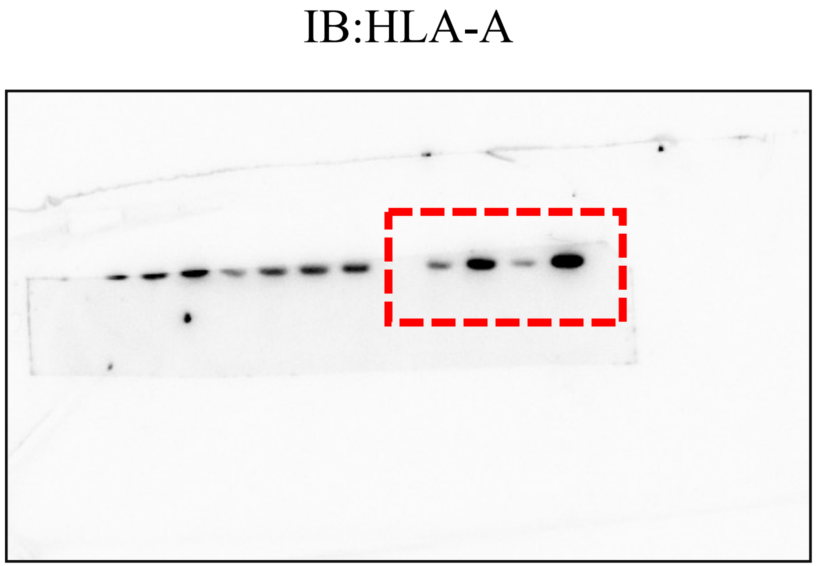

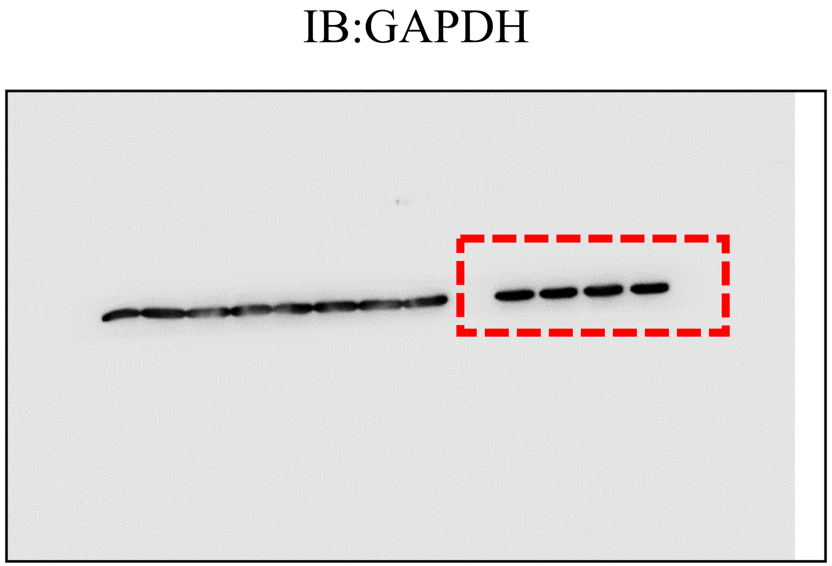

Full unedited gel for Supplemental Figure 11G

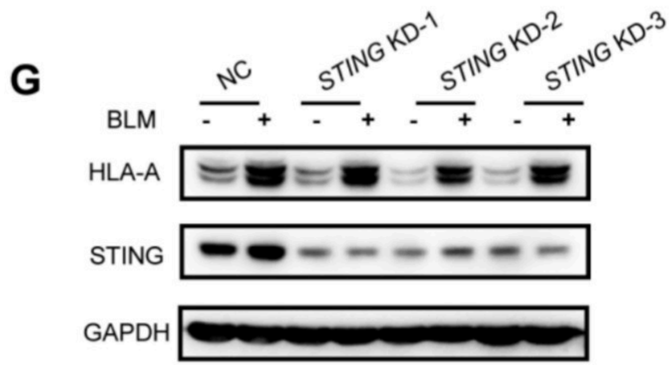

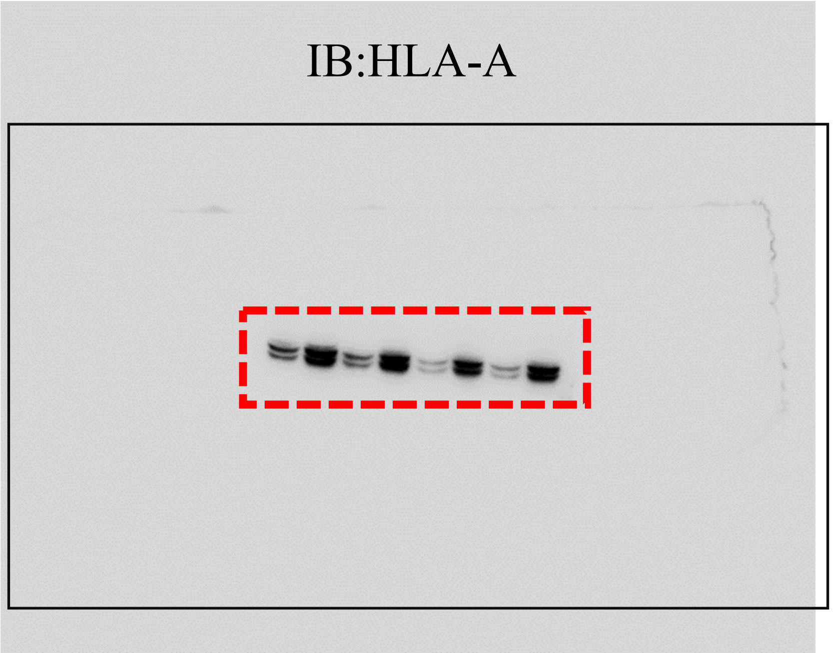

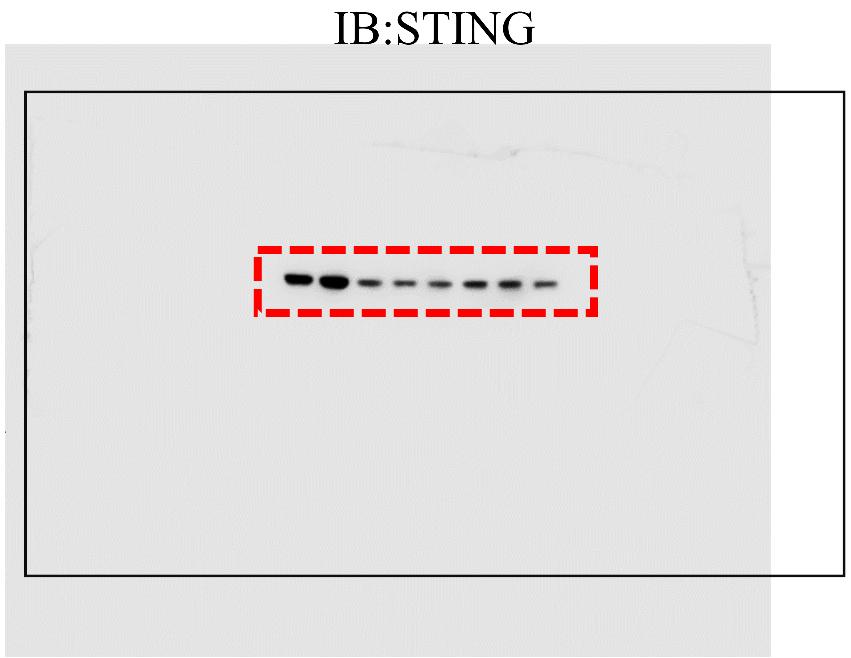

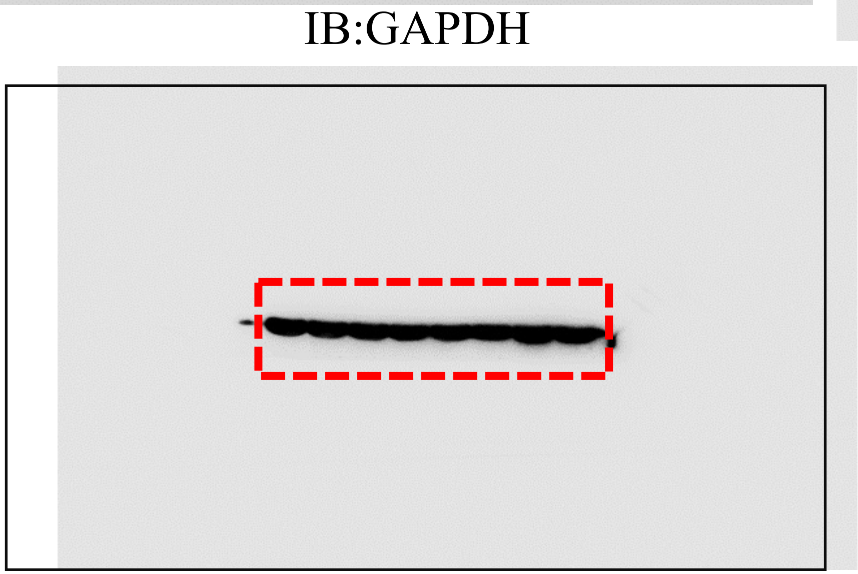

Full unedited gel for Supplemental Figure 11H

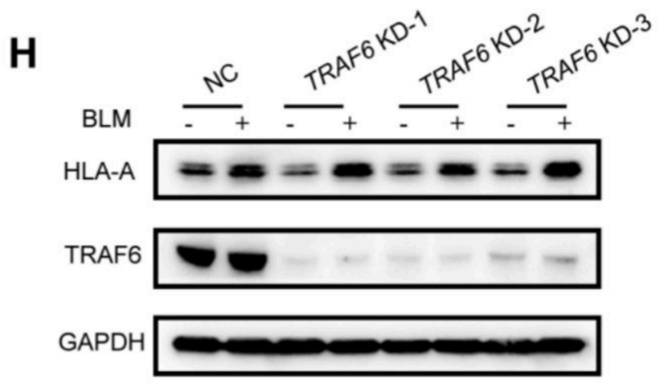

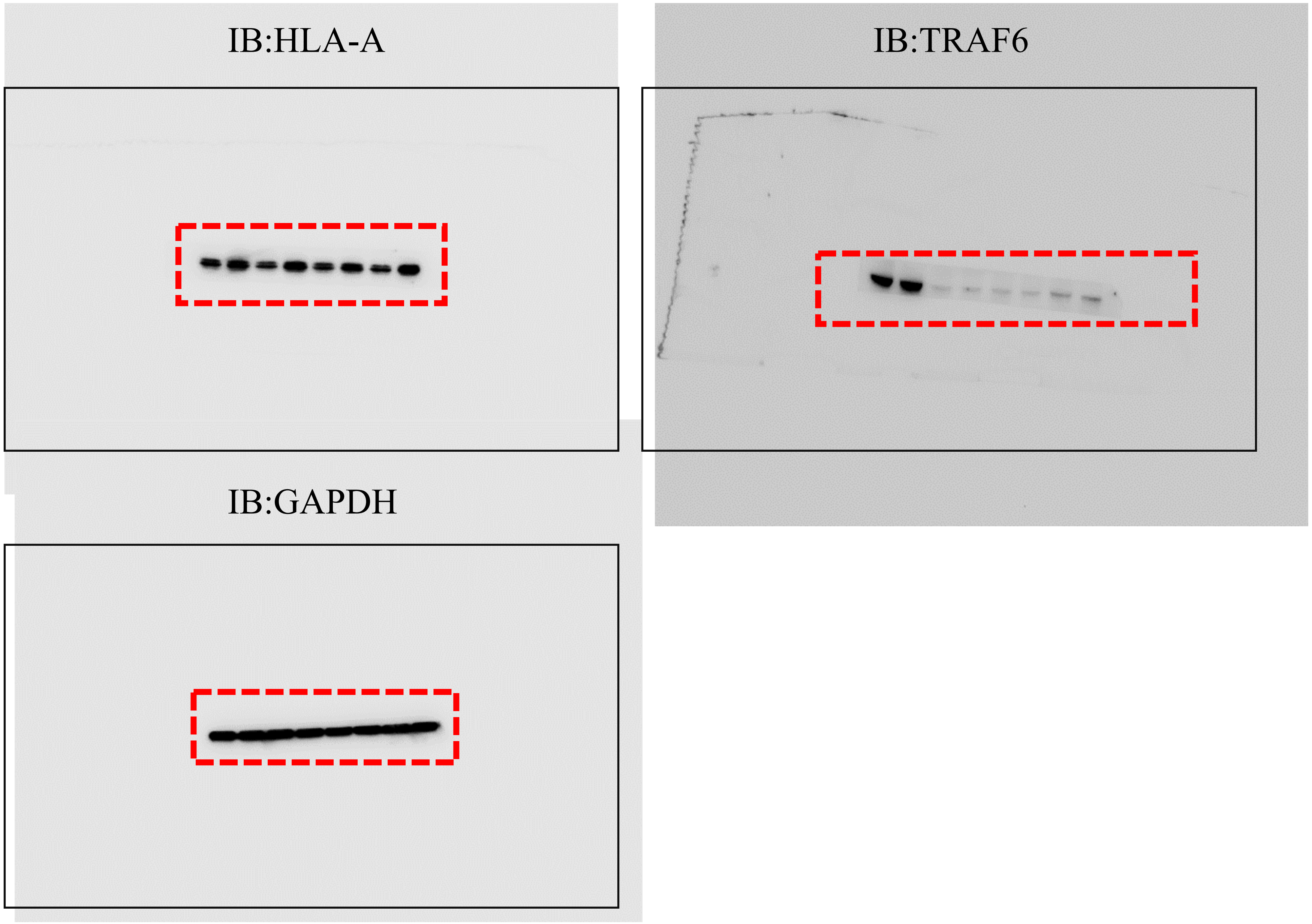

Full unedited gel for Supplemental Figure 11I

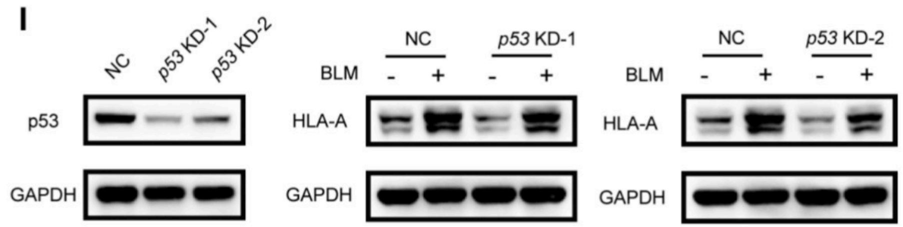

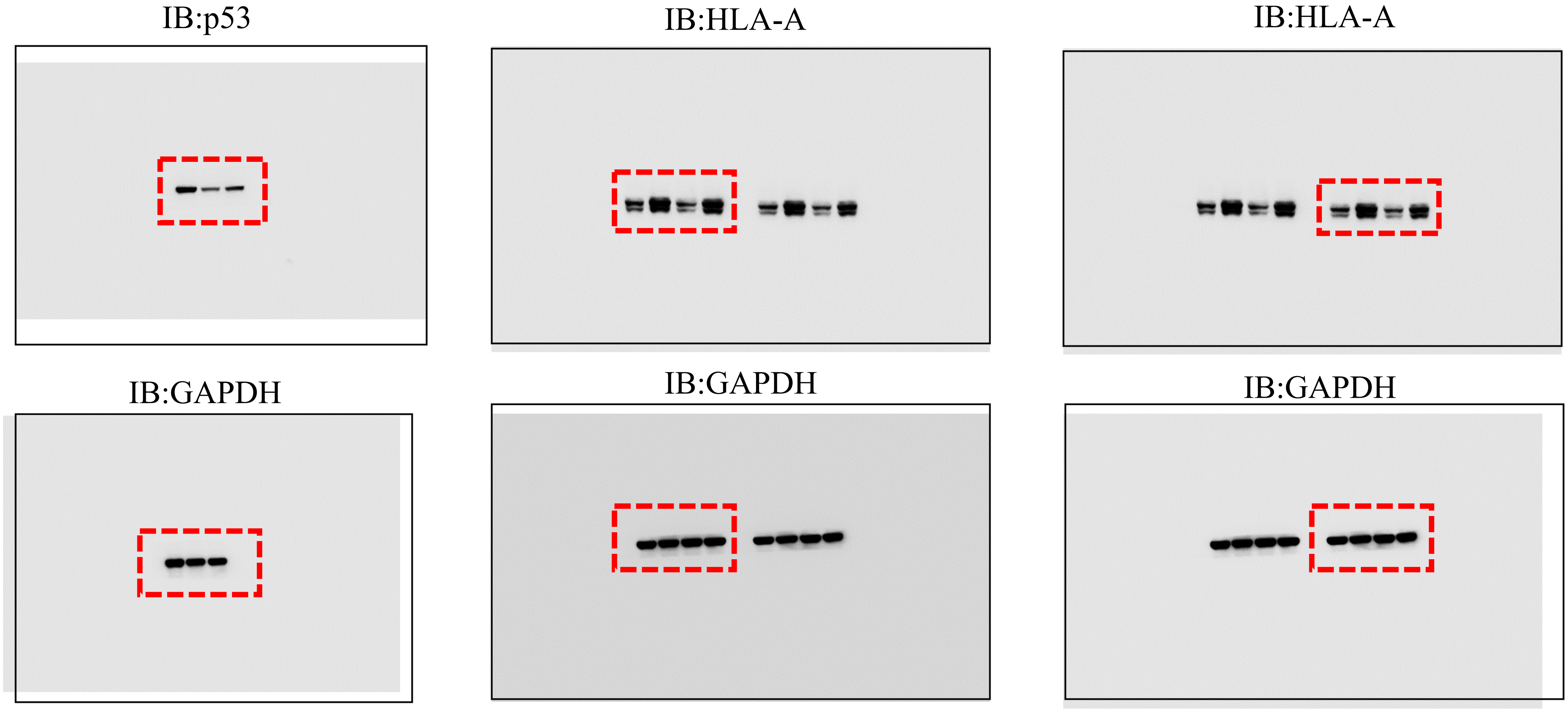

Full unedited gel for Supplemental Figure 11J

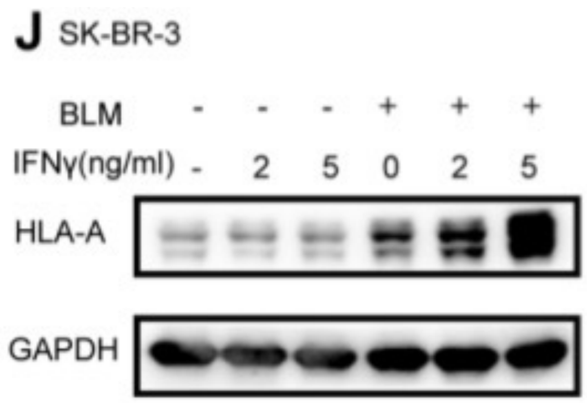

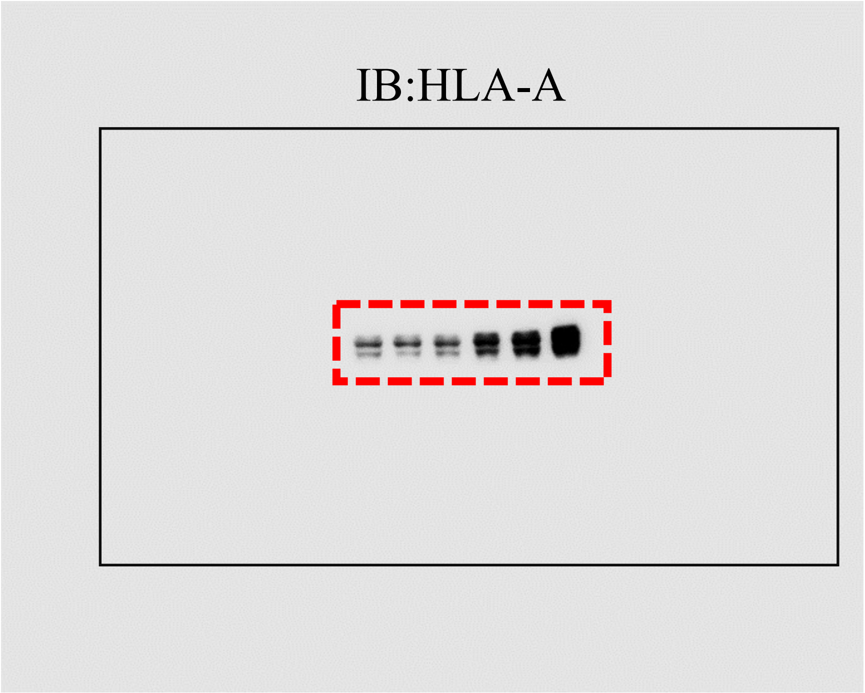

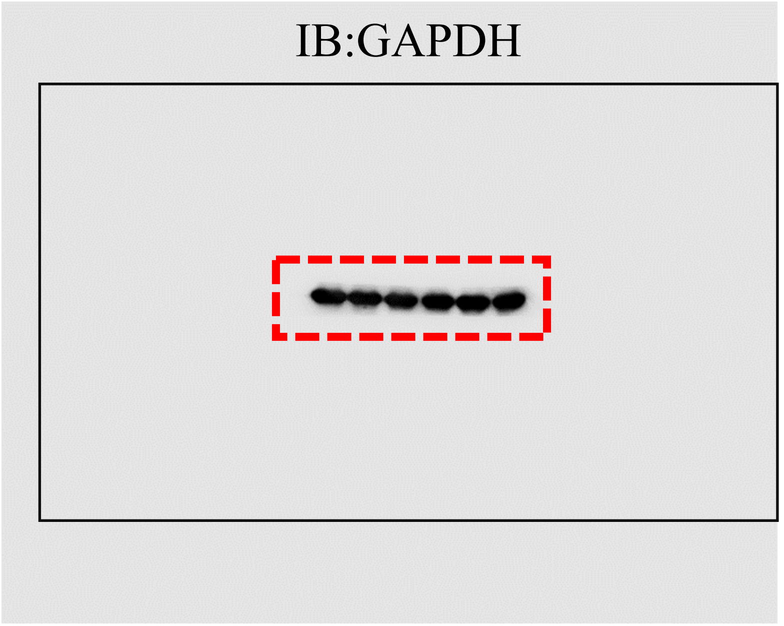

Full unedited gel for Supplemental Figure 11K

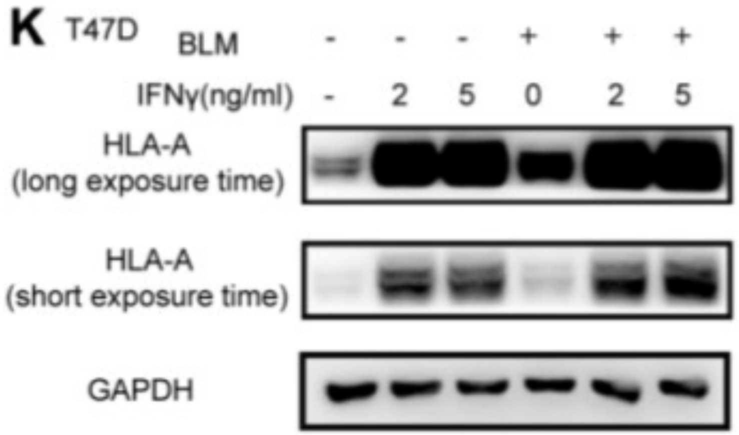

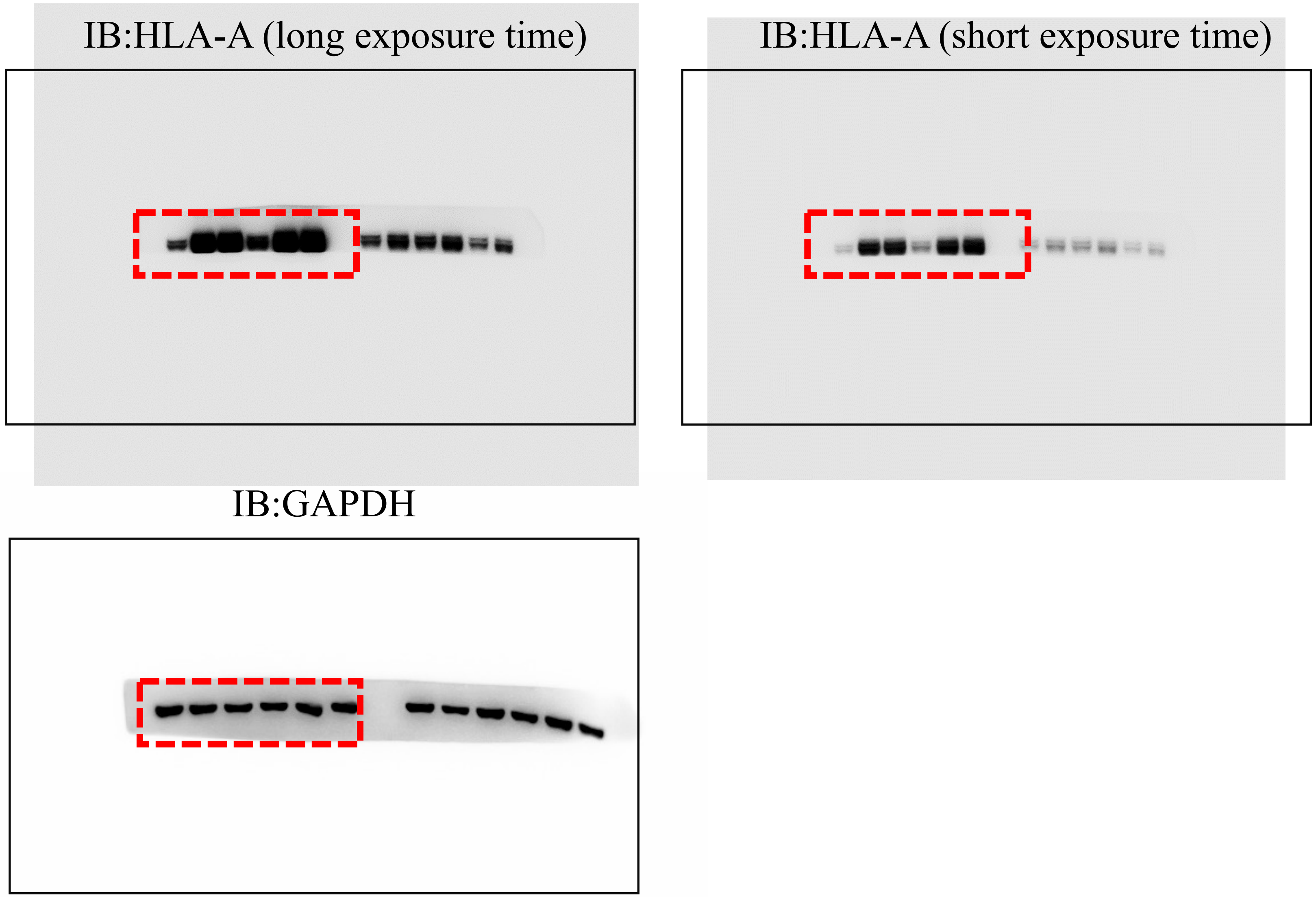

Full unedited gel for Supplemental Figure 15A

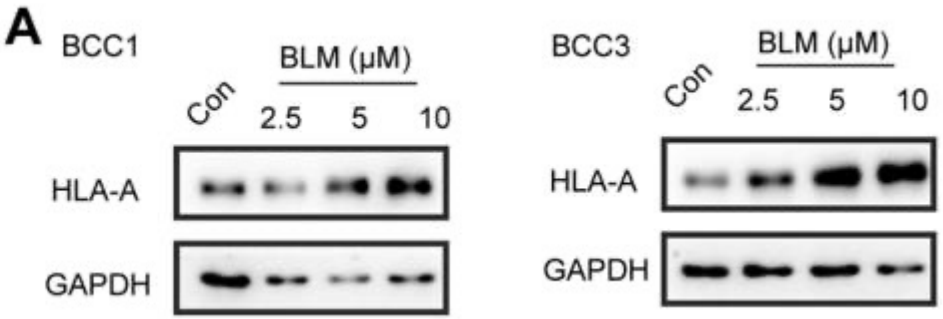

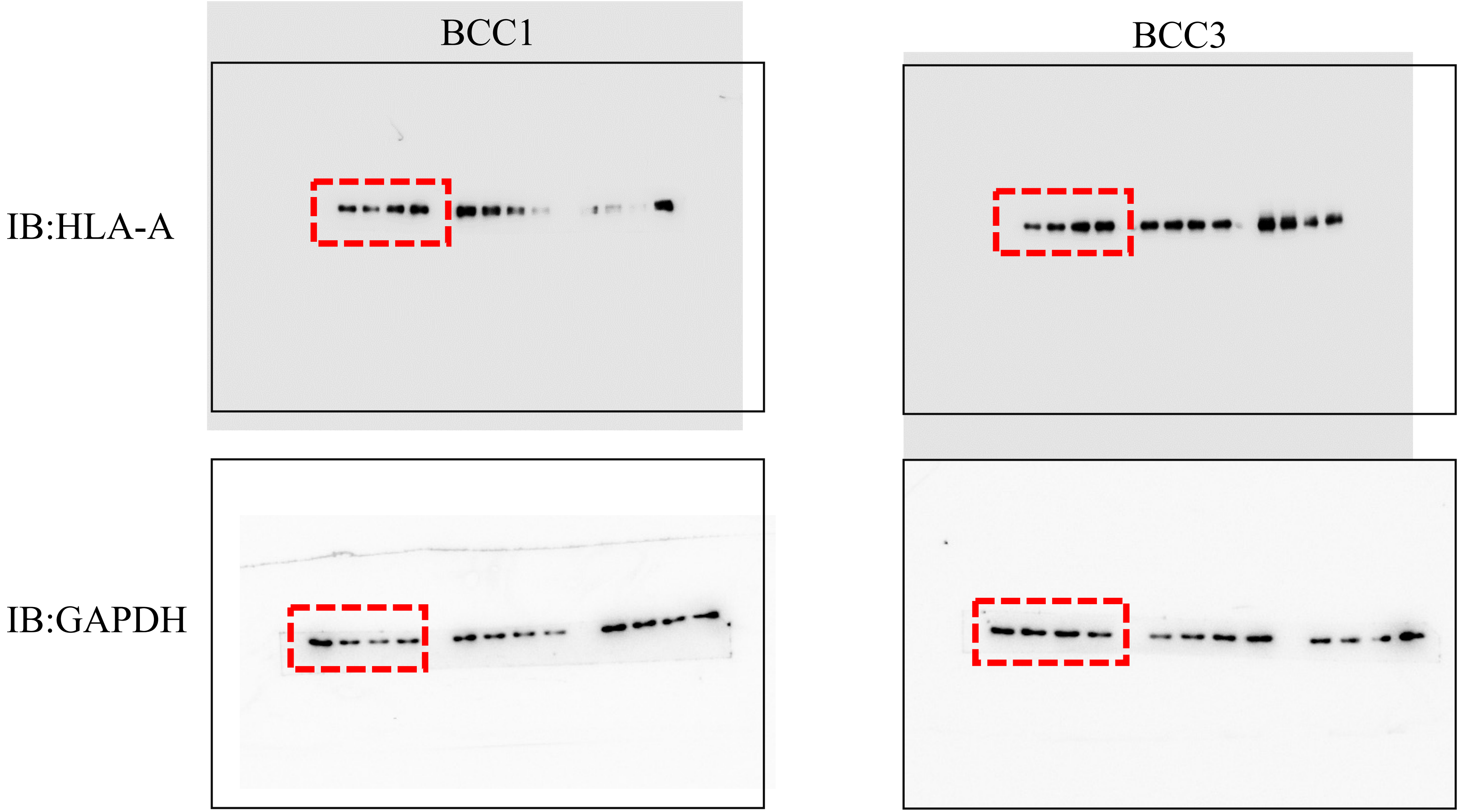

Full unedited gel for Supplemental Figure 15A

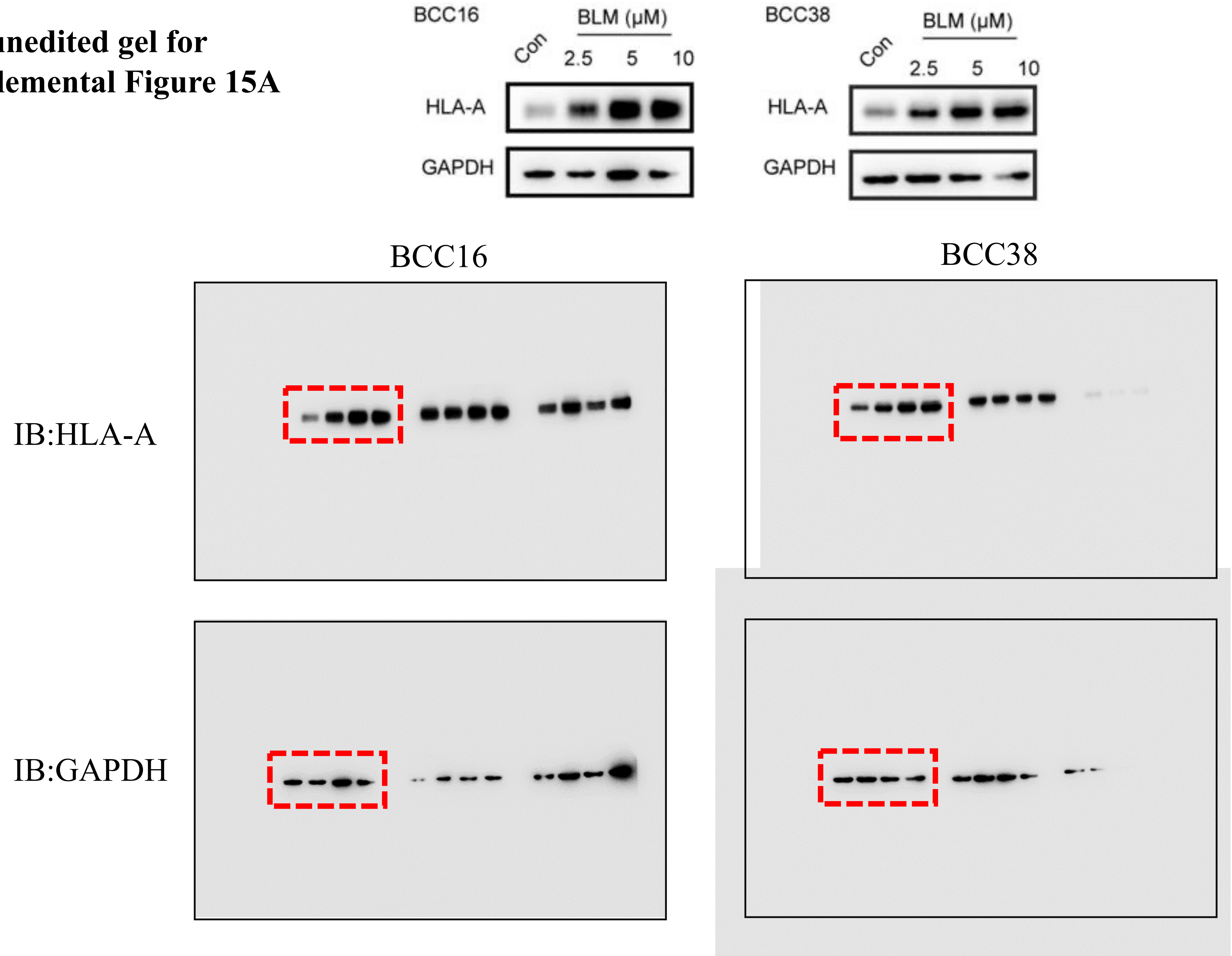

Full unedited gel for Supplemental Figure 15A

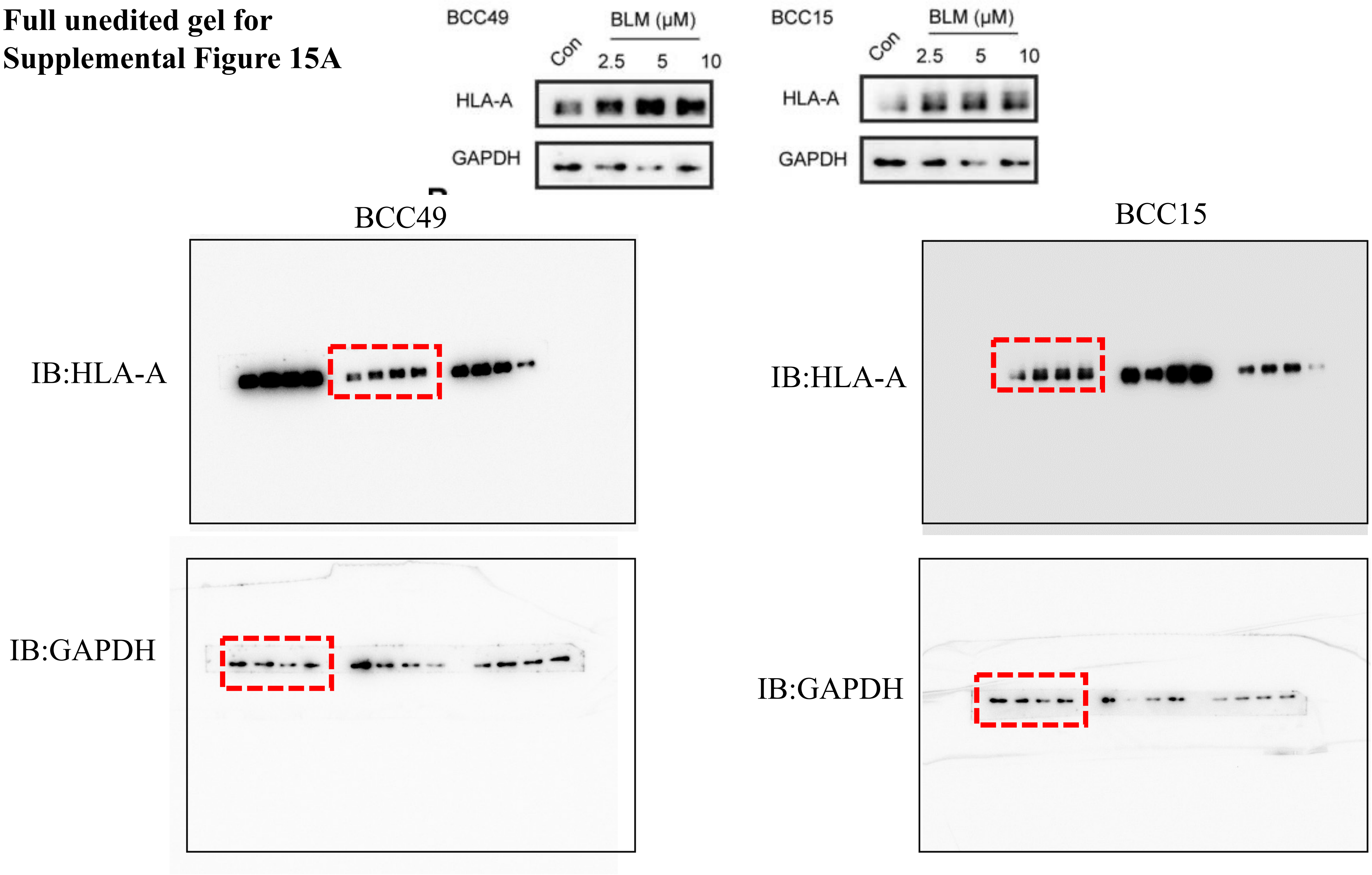

Full unedited gel for Supplemental Figure 15A

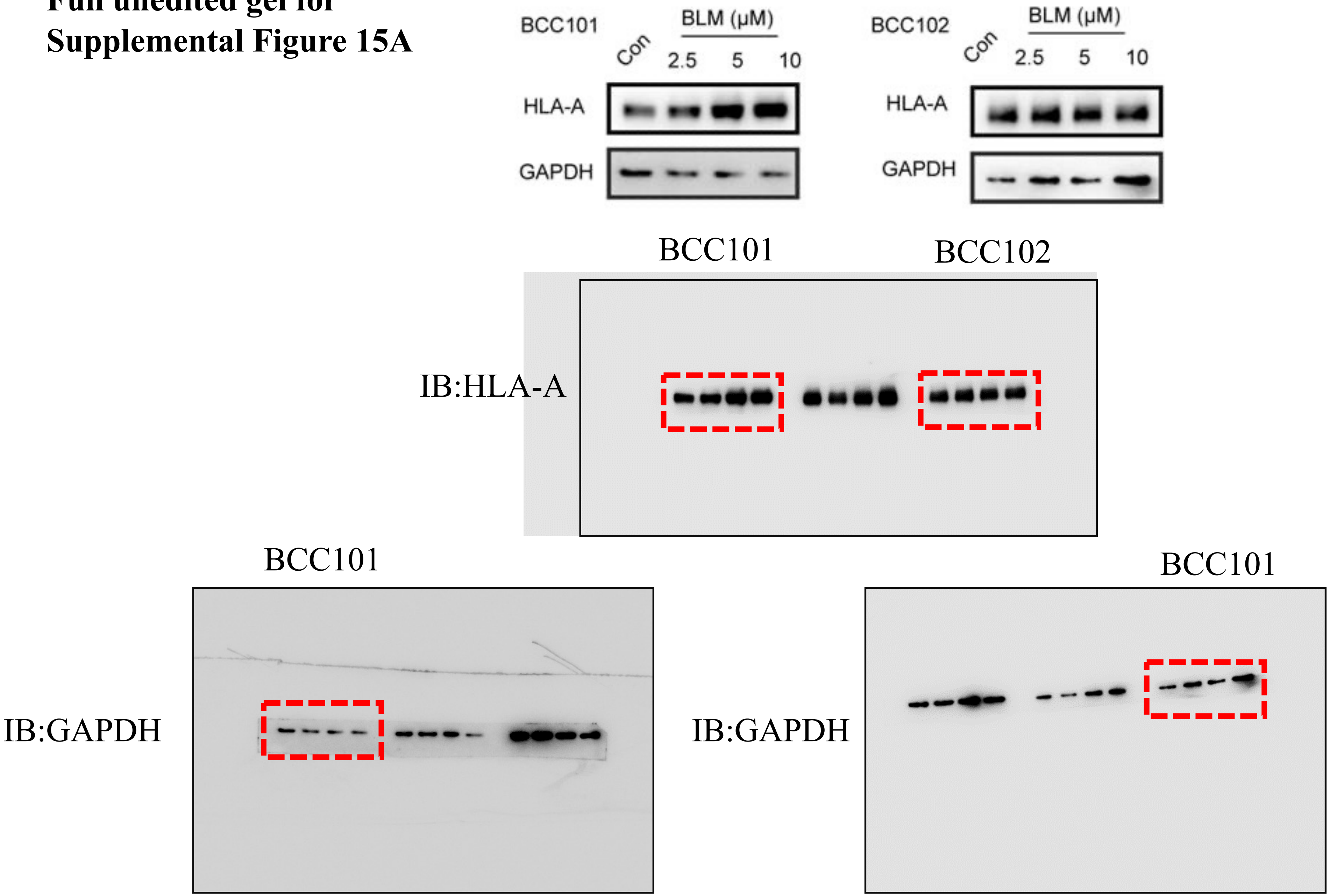

Supplement: Unedited blot and gel images [file jciinsight-9-177788-s238.pdf]
